# Aromatic heterocycle galectin-1 interactions for selective single-digit nM affinity ligands†

**DOI:** 10.1039/c8ra04389b

**Published:** 2018-07-11

**Authors:** Kristoffer Peterson, Patrick M. Collins, Xiaoli Huang, Barbro Kahl-Knutsson, Sofia Essén, Fredrik R. Zetterberg, Stina Oredsson, Hakon Leffler, Helen Blanchard, Ulf J. Nilsson

**Affiliations:** Centre for Analysis and Synthesis, Department of Chemistry, Lund University POB 124 SE-221 00 Lund Sweden ulf.nilsson@chem.lu.se; Institute for Glycomics, Griffith University Gold Coast Campus Queensland 4222 Australia; Department of Biology, Lund University SE-223 62 Lund Sweden; Department of Laboratory Medicine, Section MIG, Lund University BMC-C1228b, Klinikgatan 28 SE-221 84 Lund Sweden; Galecto Biotech AB, Sahlgrenska Science Park Medicinaregatan 8 A SE-413 46 Gothenburg Sweden

## Abstract

A series of 3-triazole-thiogalactosides and 3,3′-triazole-thiodigalactosides substituted with different five-membered heterocycles at the C-4 triazole position were found to have high selectivity for galectin-1. Initial studies on the 3-triazole-thiogalactosides indicated that five membered heterocycles in general gave increased affinity for galectin-1 and improved selectivity over galectin-3. The selectivity profile was similar for thiodigalactosides exemplified by 3,3′ substituted thien-3-yltriazole and thiazol-2-yltriazole, both having single-digit nM galectin-1 affinity and almost 10-fold galectin-1 selectivity. The binding interactions of a thiodigalactoside based galectin-1 inhibitor with two thien-3-yltriazole moieties were studied with X-ray crystallography. One of the thiophene moieties was positioned deeper into the pocket than previously reported phenyltriazoles and formed close contacts with Val31, Ser29, Gly124, and Asp123. The affinity and structural analysis thus revealed that steric and electronic optimization of five-membered aromatic heterocycle binding in a narrow galectin-1 subsite confers high affinity and selectivity.

## Abstract 2

A series of 3-triazole-thiogalactosides and 3,3′-triazole-thiodigalactosides substituted with different five-membered heterocycles at the C-4 triazole position were found to have high selectivity for galectin-1.

## Graphical abstract

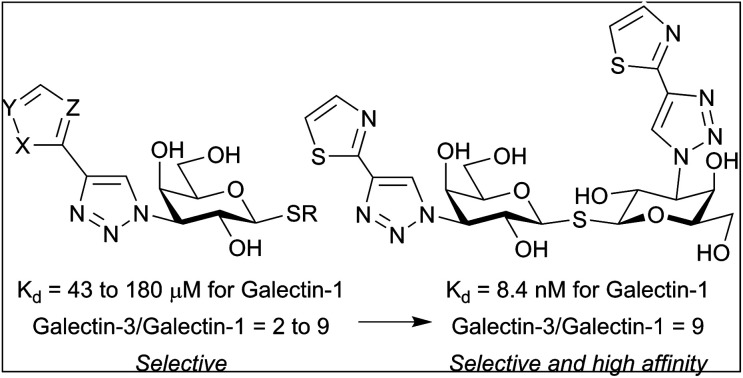

## 1. Introduction

Galectin-1 is a member of the galectin family that consists of 19 mammalian glycan-binding proteins, of which 15 are found in humans, defined by their β-galactoside affinity and the presence of conserved carbohydrate recognition domains (CRDs).^1^ Depending on how many CRDs and how they are arranged, galectins are classified as prototype, tandem-repeat or chimera galectins, with the prototype galectin-1 existing as a monomer that dimerizes at high concentrations. The CRD in galectins is a beta-sandwich where five strands make a long groove that is divided into five subsites (A–E), one per strand. The β-galactoside is located in subsite C with its C3 substituent reaching into subsites A–B and its C1 substituent reaching into subsites D–E.^2^ Galectin-1 is involved in several biological processes, *e.g.* tumor progression, inducing angiogenesis, suppressing anti-tumor immune responses and metastasis, making it an interesting therapeutic target.^3–5^

**Fig. 1:**
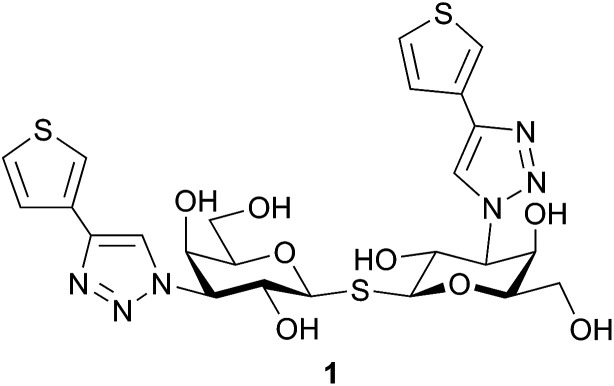
Thien-3-yl thiodigalactoside derivative 1 with high affinity for galectin-1.^14^

Small molecule galectin-1 inhibitors can serve both as potential therapeutic agents and as research tools and their synthesis often involve 1- and 3-substitutions of lactose^6,7^ and galactose^8–10^ or 3,3′-disubstitution of thiodigalactoside.^11–15^ Bis-3-(4-aryl-1,2,3-triazol-1-yl)-thiodigalactosides with high affinity towards galectin-1 and galectin-3 and selectivity over other galectins have previously been reported.^14^ In that study, five-membered heterocycles (*i.e.* thiophene 1, Fig. 1) showed enhanced galectin-1 affinity and selectivity, which motivates further studies on five-membered heterocycles as an affinity-enhancing structural element in galectin-1 inhibitors. Metabolism of thiophene-containing compounds potentially leads to reactive intermediates^16^ as observed for thienilic acid^17,18^ and clopidogrel,^19^ which is why replacement of the thiophene in 1 with another five-membered heterocycle that retains the high affinity for galectin-1 may be important. Herein, we report the synthesis and evaluation of different five-membered heterocycles at the C4-triazole position of thiogalactosides and thiodigalactosides as galectin-1 inhibitors to explore the structure–activity relationship in subsite A of the galectin-1 glycan-binding site. Furthermore, structural analysis of compound 1 in complex with galectin-1 is presented to aid in understanding its high affinity.

## 2. Results and discussion

### 2.1 Synthesis and galectin affinities of 3-(4-aryl-1,2,3-triazol-1-yl)-thiogalactosides

**Scheme 1:**
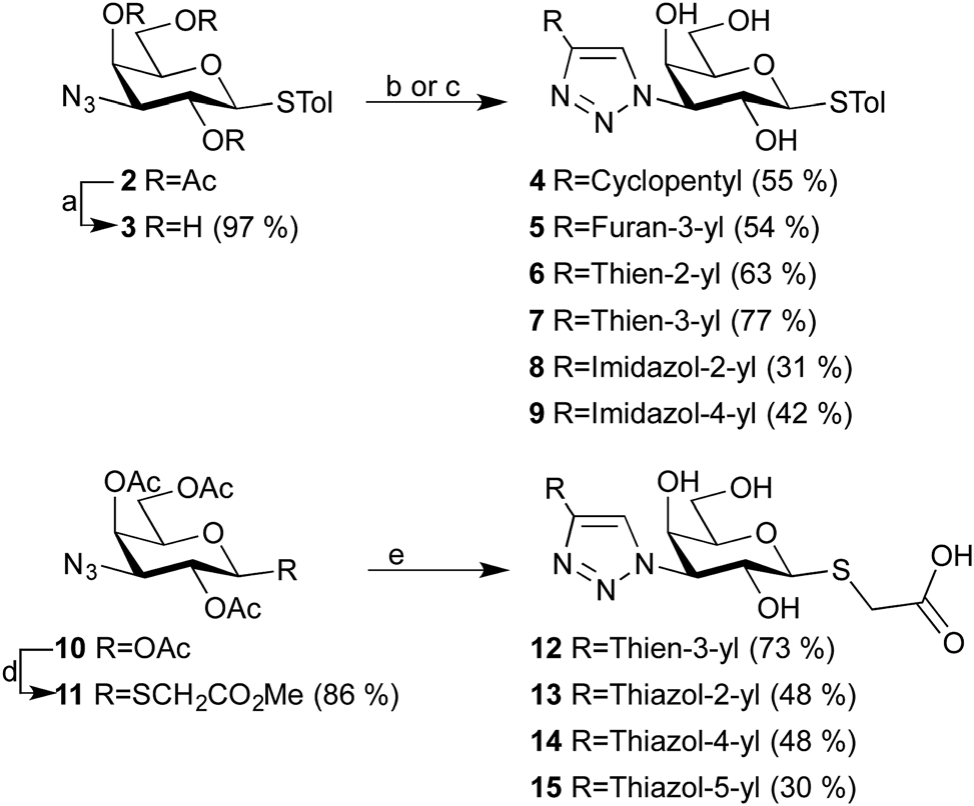
Synthesis of triazoles 4–9 and 12–15. Reagents and conditions: (a) NaOMe, MeOH, rt; (b) alkyne, CuI, DIPEA, MeCN, 50 °C; (c) (i). 1-{[2-(Trimethylsilyl)ethoxy]methyl}-4-[2-(trimethylsilyl)ethynyl]-1*H*-imidazole, CuI, DIPEA, MeCN, 50 °C; (ii). BF_3_OEt_2_, CH_2_Cl_2_, rt; (d) methyl thioglycolate, BF_3_ OEt_2_, CH_2_Cl_2_, rt; (e) (i). Alkyne, CuI, DIPEA, MeCN, 50 °C; (ii). NaOMe, MeOH, rt; (iii). LiOH, THF : H_2_O (9 : 1), rt. Tol = *p*-methylphenyl.

A series of 1,4-disubstituted triazoles 4–9 at the C3-galactose position, with different five-membered rings at the C4-triazole position were synthesized from azide 3 (obtained from deacetylation of azide 2 ^15^) through 1,3-dipolar cycloaddition (Scheme 1). The corresponding 2-thiazole analog could be synthesized, as confirmed by HRMS, but due to poor solubility satisfactory NMR spectra could not be recorded. Instead, we decided to make the thiazole analogs with a different, more polar, substituent at C1-galactose and thus prepared azide 11 by a *S*-glycosylation of azide 10 ^20^ with methyl thioglycolate. The following 1,3-dipolar cycloaddition and subsequent deprotection yielded triazoles 12–15. Triazole 12 was made to allow for a possibly better comparison between triazoles 4–9 and triazoles 13–15.

The galectin-1 and galectin-3 affinities were determined for triazoles 4–9, 12–15, reference compounds *p*-methylphenyl 1-thio-β-d-galactopyranoside 16 ^15^ and carboxymethyl 1-thio-β-d-galactopyranoside 17 ^21^ (Table 1) using a previously reported^22,23^ fluorescence polarization assay. Triazoles 5–8 and 12–15 all bound galectin-1 and galectin-3 equally or better than the corresponding unsubstituted thiogalactoside (16 or 17), while the cyclopentyltriazole 4 bound galectin-3 4-fold weaker underpinning that galectin-3 prefers aromatic triazolyl-substituents. Overall, thiogalactosides 5–9 and 12–15 bound galectin-1 better than galectin-3, clearly demonstrating the galectin-1 selectivity of five-membered heteroaryltriazoles. Thien-3-yltriazole 7 and imidazol-2-yltriazole 8 both showed 47 μM affinity towards galectin-1, while thien-2-yltriazole 6 showed slightly lower affinity. Thien-3-yltriazole 7 bound galectin-1 slightly better than thien-3-yltriazole 12, but this does not correlate with references 16 and 17 where reference 17 bound galectin-1 better than reference 16. This indicates an interplay between substituents when bound to their respective subsites A, B, and D. Thien-3-yltriazole 12 and thiazol-4-yltriazole 14 bound galectin-1 equally well, while thiazol-2-yltriazole 13 showed the highest galectin-1 affinity among triazoles 4–9 and 12–15. Introducing an oxygen (5) or a nitrogen (9 and 15) in the 3-position of the outer heterocycle lowers galectin-1 affinity 2-3-fold compared to their thien-3-yl analog.

**Table 1:** *K*_d_ (μM) values for triazoles 4–9 and 12–15 and references 16–17 determined by competitive fluorescence polarization

	Galectin-1	Galectin-3	Galectin-3/galectin-1
4	370 ± 30	980 ± 60	2.6
5	120 ± 7	250 ± 20	2.1
6	61 ± 4	180 ± 10	3.0
7	47 ± 2	200 ± 10	4.3
8	47 ± 3	220 ± 10	4.7
9	140 ± 7	330 ± 20	2.4
12	58 ± 4	200 ± 7	3.4
13	43 ± 2	280 ± 7	4.5
14	62 ± 2	540 ± 30	8.7
15	180 ± 9	430 ± 20	2.4
16	1100 ^15^	230 ^15^	0.2
17	430 ± 40	1800 ± 100	4.2

### 2.2 Synthesis and galectin affinities for symmetrical and unsymmetrical bis-3-(4-aryl-1,2,3-triazol-1-yl)-thiodigalactosides

**Scheme 2:**
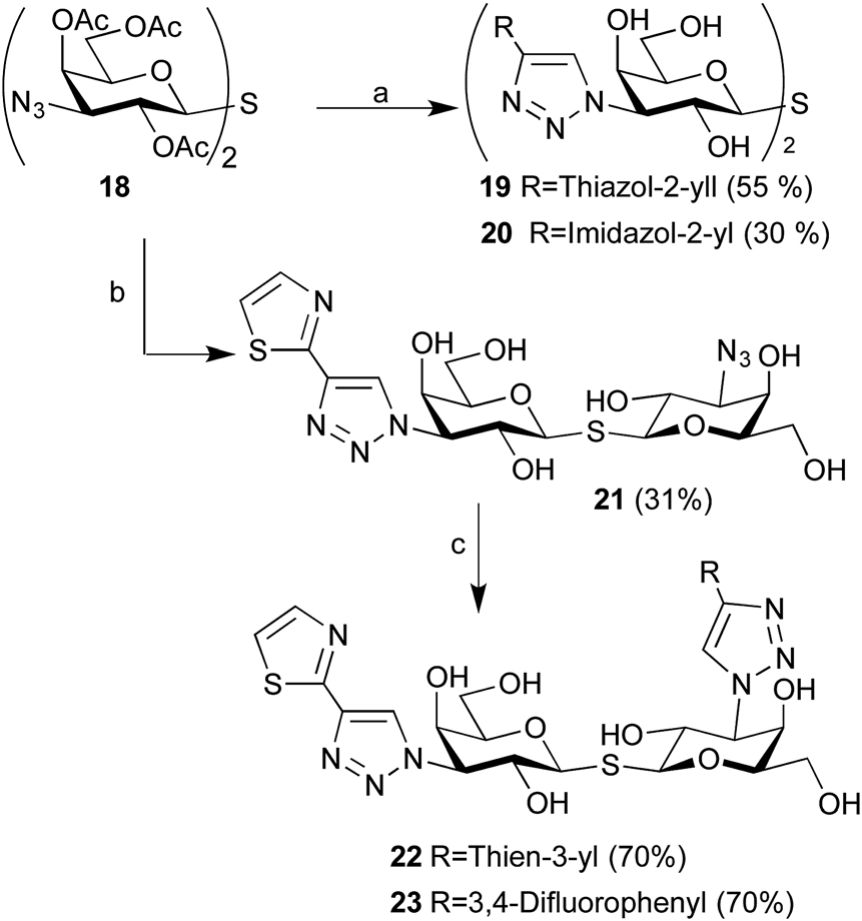
Synthesis of thiodigalactosides 19–20 and 22–23. Reagents and conditions: (a) (i). alkyne (3 equiv.), CuI, DIPEA, DMF, 50 °C; (ii). NaOMe, MeOH, rt; (b) (i). Thiazol-2-ylethynyltrimethylsilane (1.5 equiv.), CuI, DIPEA, DMF, 50 °C; (ii). NaOMe, MeOH, rt; (c) alkyne, CuI, DIPEA, DMF, 50 °C.

Having observed that imidazol-2-yl 8 and thiazol-2-yl 13 were as good or better than thien-3-yls 7 and 12 inhibiting galectin-1, the corresponding symmetrical thiodigalactosides 19–20 were synthesized from azide 18 ^24^ (Scheme 2). Two unsymmetrical bis-3-(4-aryl-1,2,3-triazol-1-yl)-thiodigalactosides having a thiazol-2-yl at one galactoside C3 and either a thien-3-yl 22 or a 3,4-difluorophenyl 23 at the other C3 were synthesized by two sequential 1,3-dipolar cycloadditions from azide 18 with different aryl-alkynes. The 3,4-difluorophenyl moiety was introduced in 23 because it has earlier been shown^15^ to result in good galectin-1 affinity.

**Scheme 3:**
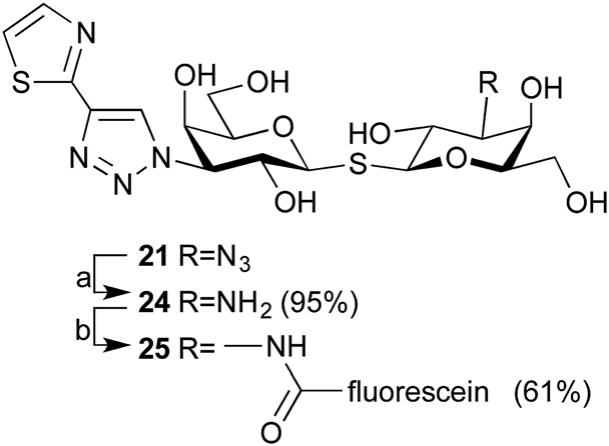
Synthesis of fluorescent probe 25. Reagents and conditions: (a) 1,3-propanedithiol, Et_3_N, MeOH, rt; (b) 5-FAM-NHS, DIPEA, DMSO, rt.

The affinity for thiodigalactosides 19–20 and 22–23 towards galectin-3 (Table 2) was determined by competitive fluorescence polarization using a recently reported probe molecule^15^ with improved affinity. Before evaluating towards galectin-1, we decided to synthesize a high affinity galectin-1 fluorescent probe molecule, which would allow lower experimental concentrations of the probe molecule and the protein in the range of the anticipated dissociation constants for the inhibitors. Reduction of azide 21 to amine 24 and amide coupling with 5-FAM-NHS resulted in fluorescent probe molecule 25 (Scheme 3). Compound 25 equipped with the affinity-enhancing thiazol-2-yltriazole moiety and fluorescein displayed improved galectin-1 affinity (*K*_d_ 65 nM, Fig. S1†). Thus, competitive fluorescence polarization experiments with 25 could be performed at lower concentrations of both the probe (1.0 nM) and galectin-1 (50 nM), resulting in improved assay sensitivity and accuracy, although a blocking protein (100 nM BSA) was required to prevent perturbing absorption losses at such low concentrations.

**Table 2:** *K*_d_ (nM) values for thiodigalactosides 1, 19–20, and 22–23 determined by competitive fluorescence polarization

	Galectin-1	Galectin-3	Galectin-3/galectin-1
1	6.1 ± 1	59 ± 4	9.7
19	8.4 ± 1	74 ± 4	8.8
20	25 ± 2	180 ± 10	7.2
22	12 ± 1	61 ± 3	5.1
23	13 ± 1	1.8 ± 0.2	0.14
TDG	24 000 ^13^	49 000 ^13^	2

Thiodigalactosides 1, 19–20, and 22–23 showed much higher affinities towards galectin-1 and galectin-3 than unsubstituted thiodigalactoside (TDG) and excellent galectin-1 affinity in the 6–13 nM range was observed with thiodigalactosides 1, 19, and 22–23. The imidazol-2-yl thiodigalactoside 20 had 4-fold lower affinity for galectin-1 than thiophene 1, which is interesting considering the corresponding monogalactosides (7–8) bound approximately equally well. Hence, while one imidazol-2-yl-triazole moiety induced improved affinity and selectivity for galectin-1, addition of a second imidazol-2-yl-triazole moiety on a thiodigalactoside scaffold apparently reduces the overall affinity enhancement, which leads to weaker affinity than the corresponding thiophene- and thiazole derivatives 1 and 19. Thiodigalactoside 23, with a difluorinated phenyl substituent, had higher affinity towards galectin-3 than galectin-1, which is in line with earlier observations of fluorophenyl groups enhancing galectin-3 affinities.^15^ Thiodigalactosides 1, 19–20, and 22 bound galectin-3 in the 50–200 nM range resulting in a 2-fold increased galectin-1 selectivity (7-10-fold in total) compared to their corresponding monogalactoside, indicating that the five-membered heterocycles on both sides of the core thiodigalactoside form more favorable interactions with galectin-1 than galectin-3. In order to have a first result on the possible suitability on thienyl and thiazolyl-galactose derivatives as drug leads, thiophenes 1, 6–7, and 12 and thiazoles 13–14, and 19 were tested for cytotoxicity against tumor cell lines JIMT-1 and MCF-7 and the normal cell line MCF-10A. All compounds were found to be non-cytotoxic at the indicated concentrations, which suggests that these compounds do not possess immediate cytotoxicity that would interfere with use in cell or *in vivo* assays (Fig. S2†).

### 2.3 Structural analysis

**Fig. 2:**
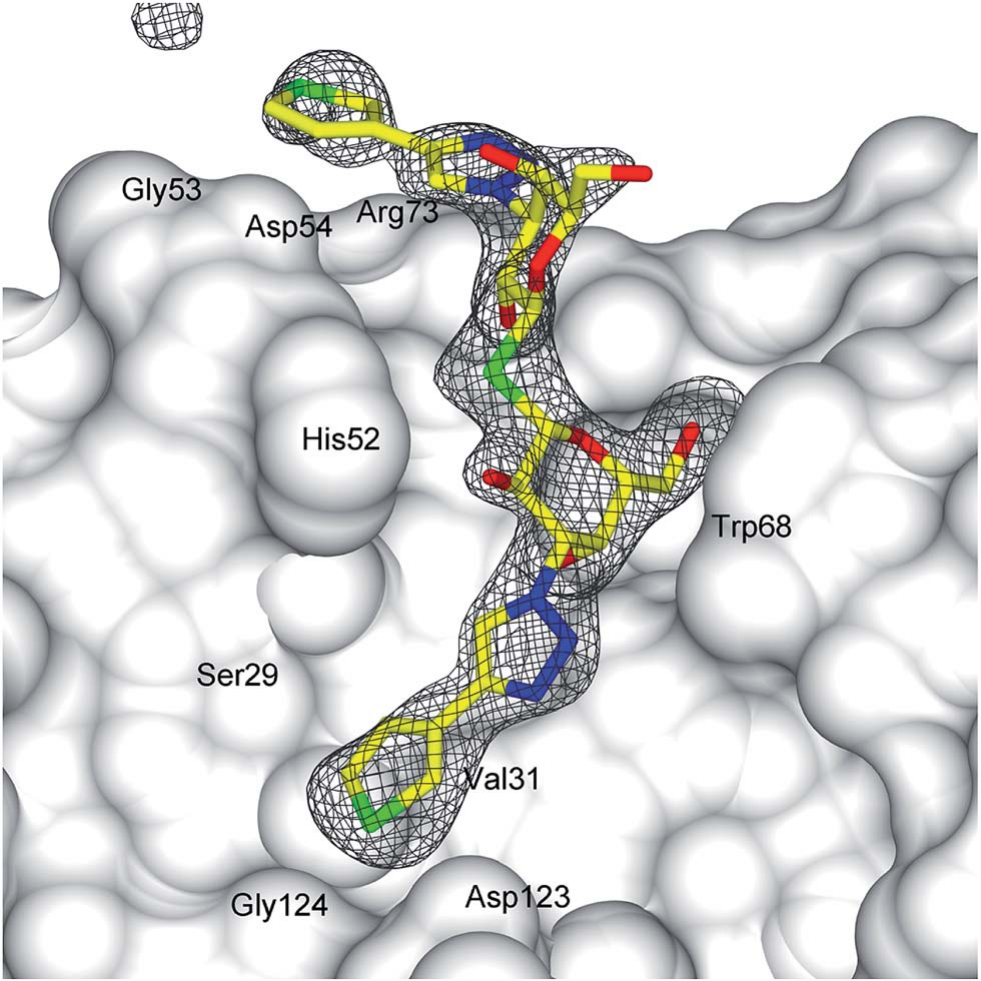
Difference electron density of 1 in the galectin-1 binding site. Difference electron density calculated from refinement with the ligand (stick representation) omitted from the model (|*F*_o_| − |*F*_c_| *α*_calc_; grey mesh, contoured at 3*σ*) and the protein represented by a grey solvent-accessible surface. The narrow pocket accommodating one of the thiophene rings is indicated with a yellow arrow.

**Fig. 3:**
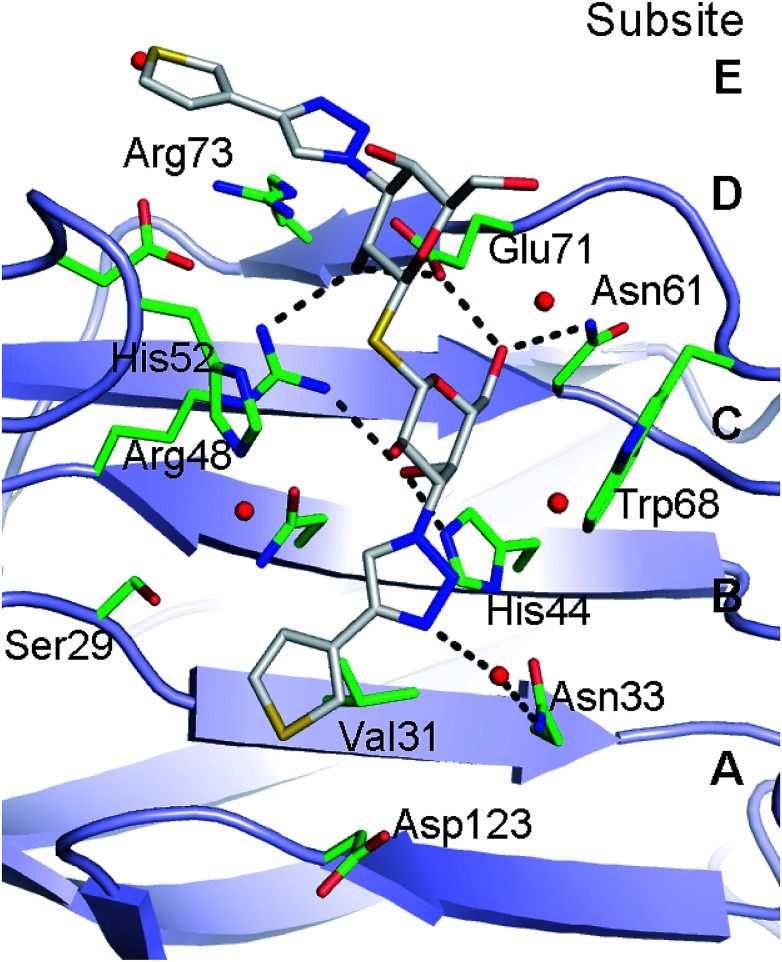
Galectin-1 binding site interactions with 1. H-bond interactions between ligand (carbons white) and protein/water (carbons green) are shown as dashed lines.

Lectin-ligand interactions are often comparatively weak and the high affinity observed of 1 towards galectin-1 obviously raises questions about the underlying molecular interactions. Hence, the X-ray structure of thiophene 1 was determined with galectin-1 to be compared to the corresponding published^14^ galectin-3 structure. Refinement of X-ray diffraction data (2.2 Å resolution, Fig. 2, Table S1†) showed electron density clearly evident for the thiodigalactoside core of 1 (except for one solvent orientated C6 hydroxyl) and for both triazole rings extending from the C3 positions of the two galactose residues (Fig. 2). The conformations of both thiophene rings can be identified from a higher electron density peak corresponding to the higher atomic weight sulfur atom. Compound 1 binds to galectin-1 with the thiodigalactoside core of 1 forming the same protein–ligand interactions as observed in the thiodigalactoside-bound galectin-1 complex.^25^ The triazole ring that is positioned close to the Asp54–Arg73 salt bridge in subsite E (Fig. 2 and 3) forms a stacking interaction with Arg73. One thiophene ring of 1 is positioned in a narrow pocket between Ser29 and Asp123 in subsite A–B with an apparently ideal steric fit of the five-membered thiophene ring of 1 (Fig. 2 and 3). This allows the ligand to be positioned deeper within the pocket (drawing the triazole ring away from Trp68) compared to the bulkier six-membered 3-fluorophenyl ring of 1,1′-sulfanediyl-bis-{3-deoxy-3-[4-(3-fluorophenyl)-1*H*-1,2,3-triazol-1-yl]-β-d-galactopyranoside}.^26^ Furthermore, the same thiophene ring of 1 forms a close contact with Val31 in subsite A (ring centroid to Val31 γ-carbon is 3.4 Å) and with Ser29 and Gly124 within subsite A. The thiophene ring positioned at the galectin-1 binding site near the Asp54–Arg73 salt bridge do not interact as closely with the protein, but are within potential van der Waals contact to the side-chains of Gly53, Asp54, and Arg73 in subsite E.

**Fig. 4:**
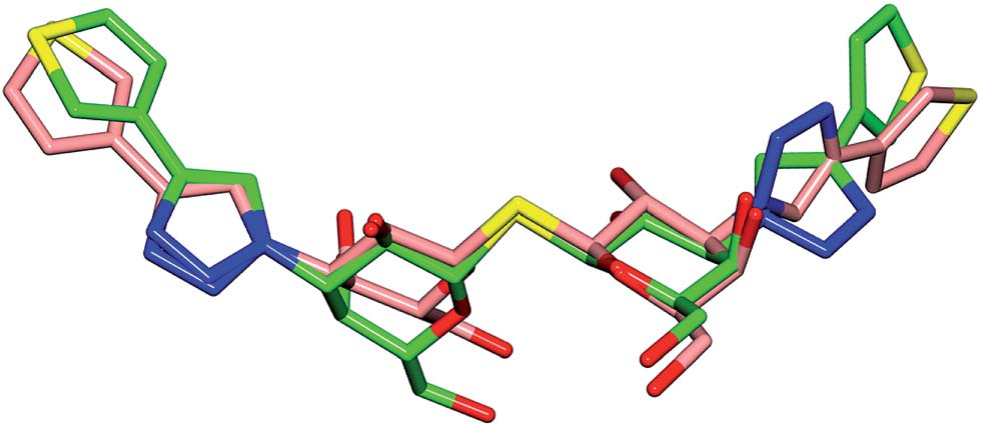
Comparison of the binding conformation of 1 when bound to galectin-1 (carbons pink) and galectin-3 ^14^ (carbons green).

Comparison of the galectin-3 : 1 complex^14^ to the galectin-1 : 1 complex reveals the high degree of similarity in the ligand conformation when bound to either galectin-1 or galectin-3 (Fig. 4) with the main differences in protein–ligand interactions occurring in the location of the terminal thiophene rings. The C–N bond between the triazole N1 and galactose C3 that is positioned close to the galectin-1 binding site Arg73–Asp54 salt bridge is rotated through ∼180° resulting in the thiophene in the galectin-3 complex to be positioned over the salt bridge Arg186, whereas it is positioned more directly over the center of the Arg73–Asp54 salt bridge in galectin-1 (Fig. 3). At the other end of the ligand, the thiophene ring is flipped 180° *via* rotation about the triazole–thiophene bond. In galectin-3 this results in the sulfur atom being buried in the Arg144 hydrophobic pocket and making contacts with Ala146 and Arg144,^14^ while in the galectin-1 complex the sulfur atom of 1 makes alternative contacts with the Asp123 carboxylate and backbone nitrogen (Fig. 3). The high degree of similarity in the binding conformation of 1 in complex with galectin-1 or galectin-3 is interesting considering the key differences in binding site amino acid residues are in the location of Ser29 in galectin-1 and Arg144 in galectin-3. The equivalent of these amino acids is absent in some galectins^27^ and targeting ligand interactions to this region is a possible means of enhancing galectin-binding selectivity.

## 3. Experimental

### 3.1 General

All reagents and solvents were dried prior to use according to standard methods and commercial reagents were used without further purification. Non-commercial acetylene derivatives were synthesized according to published procedures.^28–30^ Analytical TLC was performed using on silica gel 60 F_254_ (Merck) with detection by UV absorption and/or by charring following immersion in a 7% ethanolic solution of sulfuric acid. Purification of compounds was carried out by column chromatography on silica gel (40–60 μm, 60 Å) and/or preparative HPLC (Agilent 1260 infinity system, column SymmetryPrep-C18, 17 mL min^−1^ H_2_O–MeCN gradient 10–100% 15 min with 0.1% formic acid). Specific rotations were measured on a Perkin Elmer model 341 polarimeter. NMR spectra ^1^H, ^13^C, 2D COSY and HMQC were recorded with a Bruker Avance II 400 MHz spectrometer (400 Hz for ^1^H, 100 Hz for ^13^C) or a Bruker Avance III 500 MHz spectrometer (500 Hz for ^1^H, 125 Hz for ^13^C) at ambient temperature. Chemical shifts are reported in *δ* parts per million (ppm). HRMS was determined by direct infusion on a Waters XEVO-G2 QTOF mass spectrometer using electrospray ionization. Compounds 4–9, 12–15, 19–20, 22–23, and 25 were of >95% purity according to HPLC-analysis (Agilent series 1100 system, column Eclipse XDB-C18, 0.8 mL min^−1^ H_2_O–MeCN gradient 5–95% 13 min with 0.1% trifluoroacetic acid) or UPLC (Waters Acquity UPLC system, column Waters Acquity CSH C18, 0.5 mL min^−1^ H_2_O–MeCN gradient 5–95% 10 min with 0.1% formic acid) analysis.

### 3.2 Synthesis of azides (3) and (11)

#### 3.2.1 *p*-Methylphenyl 3-azido-3-deoxy-1-thio-β-d-galactopyranoside (3)

NaOMe (1 M, 5 mL) was added to a solution of *p*-methylphenyl 2,4,6-tri-*O*-acetyl-3-azido-3-deoxy-1-thio-β-d-galactopyranoside (813 mg, 1.86 mmol) in MeOH (25 mL) and the resulting solution was stirred overnight. After neutralization with Dowex 50W-X8 (H^+^) resin and filtration the product was purified with column chromatography (heptane : EtOAc 1 : 1 to >2 : 3) to give 3 (561 mg, 97%) as an amorphous white solid. [*α*]^20^_D_ −22.4 (c 0.90, CH_3_OH). ^1^H NMR (CD_3_OD, 400 MHz): *δ* 7.45 (d, *J* = 8.1 Hz, 2H, Ph), 7.12 (d, *J* = 8.1 Hz, 2H, Ph), 4.56 (d, *J* = 9.8 Hz, 1H, H-1), 3.95 (d, *J* = 3.1 Hz, 1H, H-4), 3.78 (t, *J* = 9.8 Hz, 1H, H-2), 3.75–3.65 (m, 2H, H-6), 3.55 (t, *J* = 6.1, 1H, H-5), 3.37 (dd, *J* = 9.9, 3.0 Hz, 1H, H-3), 2.31 (s, 3H, CH_3_). ^13^C NMR (CD_3_OD, 100 MHz): *δ* 138.6, 133.0, 131.8, 130.6, 91.2, 80.8, 69.5, 69.4, 68.5, 62.4, 21.1. HRMS calculated for [C_13_H_17_N_3_O_4_SNa]+, 334.0837; found: 334.0839.

#### 3.2.2 Methoxycarbonylmethyl 2,4,6-tri-*O*-acetyl-3-azido-3-deoxy-1-thio-β-d-galactopyranoside (11)

Boron trifluoride diethyl etherate (0.12 mL, 1.01 mmol) was added to a cooled solution of 1,2,4,6-tetra-*O*-acetyl-3-azido-3-deoxy-β-d-galactopyranoside (250 mg, 0.67 mmol) and methyl thioglycolate (0.07 mL, 0.80 mmol) in CH_2_Cl_2_ (8 mL) and the resulting mixture was stirred 6 h at rt. The reaction mixture was diluted with CH_2_Cl_2_, washed with sat. aq. NaHCO_3_ and brine and the organic phase was dried and evaporated. The obtained residue was purified with column chromatography (heptane : EtOAc 3 : 1 to >2 : 1) to give 11 (241 mg, 86%) as an transparent oil. [*α*]^20^_D_ −44.3 (c 0.80, CH_3_OH). ^1^H NMR (CDCl_3_, 400 MHz): *δ* 5.45 (dd, *J* = 3.3, 1.0 Hz, 1H, H-4), 5.19 (t, *J* = 10.0 Hz, 1H, H-2), 4.63 (d, *J* = 9.8 Hz, 1H, H-1), 4.10 (dd, *J* = 11.6, 6.4 Hz, 1H, H-6), 4.05 (dd, *J* = 11.6, 6.4 Hz, 1H, H-6), 3.89 (td, *J* = 6.5, 1.1 Hz, 1H, H-5), 3.74 (s, 3H, CH_3_), 3.67 (dd, *J* = 10.1, 3.3 Hz, 1H, H-3), 3.53 (d, *J* = 15.0 Hz, 1H, CH_2_), 3.32 (d, *J* = 15.0 Hz, 1H, CH_2_), 2.17 (s, 3H, Ac), 2.14 (s, 3H, Ac), 2.06 (s, 3H, Ac). ^13^C NMR (CDCl_3_, 100 MHz): *δ* 170.6, 170.3, 170.1, 169.7, 83.2, 75.5, 68.3, 67.8, 62.8, 61.6, 52.7, 31.1, 20.9, 20.8, 20.7. HRMS calculated for [C_15_H_22_N_3_O_9_S]^+^, 420.1077; found: 420.1083.

### 3.3 General procedure for preparation of triazoles (4–8) and procedure for preparation of triazole (9)

To a solution of *p*-methylphenyl 3-azido-3-deoxy-1-thio-β-d-galactopyranoside 3 (*m*) and CuI (0.25 equiv.) in MeCN (3 mL) the corresponding acetylene derivative (x, 1.5 equiv.) and diisopropylethylamine (*y* equiv.) were added. The mixture was stirred (*t*) h at 50 °C before quenching with sat. aq. NH_4_Cl followed by evaporation of the solvent. The residue was purified with column chromatography (heptane : EtOAc 2 : 3 to >1 : 3) to give the product as an amorphous white solid.

#### 3.3.1 *p*-Methylphenyl 3-deoxy-3-(4-cyclopentyl-1*H*-1,2,3-triazol-1-yl)-1-thio-β-d-galactopyranoside (4)

*m* = 21 mg, x = cyclopentylacetylene, *y* = 1, *t* = 30 h. Yield 15 mg, 55%. [*α*]^20^_D_ 37.8 (c 1.25, CH_3_OH). ^1^H NMR (CD_3_OD, 400 MHz): *δ* 7.79 (s, 1H, Ph), 7.49 (d, *J* = 8.1, 2H, Ph), 7.15 (d, *J* = 8.1, 2H, Ph), 4.75 (dd, *J* = 10.5, 3.0 Hz, 1H, H-3), 4.74 (d, *J* = 9.5 Hz, 1H, H-1), 4.18 (dd, *J* = 10.5, 9.6 Hz, 1H, H-2), 4.08 (d, *J* = 2.9 Hz, 1H, H-4), 3.79–3.67 (m, 3H, H-5 and H-6), 3.16 (quint, *J* = 8.3 Hz, 1H, CH), 2.33 (s, 3H, CH_3_), 2.14–2.06 (m, 2H, CH_2_), 1.83–1.64 (m, 6H, CH_2_). ^13^C NMR (CD_3_OD, 100 MHz): *δ* 138.7, 133.1, 131.7, 130.6, 91.7, 80.9, 69.6, 69.0, 67.9, 62.3, 38.1, 34.2, 34.1, 26.1, 21.1. HRMS calculated for [C_20_H_28_N_3_O_4_S]+, 406.1801; found: 406.1804.

#### 3.3.2 *p*-Methylphenyl 3-deoxy-3-[4-(furan-3-yl)-1*H*-1,2,3-triazol-1-yl]-1-thio-β-d-galactopyranoside (5)

*m* = 20 mg, x = furan-3-ylethynyltrimethylsilane, *y* = 2, *t* = 8 h. Yield 14 mg, 54%. [*α*]^20^_D_ 49.5 (c 0.90, CH_3_OH). ^1^H NMR (CD_3_OD, 400 MHz): *δ* 8.21 (s, 1H, Ph), 7.95 (dd, *J* = 1.5, 0.8 Hz, 1H, Ph), 7.58 (t, *J* = 1.7 Hz, 1H, Ph), 7.50 (d, *J* = 8.1 Hz, 2H, Ph), 7.15 (d, *J* = 8.1 Hz, 2H, Ph), 6.82 (dd, *J* = 1.9, 0.8 Hz, 1H, Ph), 4.85 (dd, *J* = 10.5, 3.0 Hz, 1H, H-3), 4.77 (d, *J* = 9.5 Hz, 1H, H-1), 4.28 (dd, *J* = 10.5, 9.6 Hz, 1H, H-2), 4.13 (d, *J* = 2.9 Hz, 1H, H-4), 3.83–3.70 (m, 3H, H-5 and H-6), 2.33 (s, 3H, CH_3_). 13C NMR (CD_3_OD, 100 MHz): *δ* 145.1, 141.3, 140.7, 138.8, 133.2, 131.6, 130.7, 121.6, 118.0, 109.7, 91.7, 80.9, 69.6, 69.1, 68.0, 62.3, 21.1. HRMS calculated for [C_19_H_22_N_3_O_5_S]^+^, 404.1280; found: 404.1282.

#### 3.3.3 *p*-Methylphenyl 3-deoxy-3-[4-(thien-2-yl)-1*H*-1,2,3-triazol-1-yl]-1-thio-β-d-galactopyranoside (6)

*m* = 25 mg, x = 2-ethynylthiophene, *y* = 1, *t* = 40 h. Yield 21 mg, 63%. [*α*]^20^_D_ 56.5 (c 0.98, CH_3_OH). ^1^H NMR (CD_3_OD, 400 MHz): *δ* 8.29 (s, 1H, Ph), 7.50 (d, *J* = 8.1 Hz, 2H, Ph), 7.41 (m, 2H, Ph), 7.15 (d, *J* = 8.1 Hz, 2H, Ph), 7.09 (dd, *J* = 5.1, 3.6 Hz, 1H, Ph), 4.86 (dd, *J* = 10.5, 3.0 Hz, 1H, H-3), 4.77 (d, *J* = 9.5 Hz, 1H, H-1), 4.28 (t, *J* = 10.0 Hz, 1H, H-2), 4.14 (d, *J* = 2.9 Hz, 1H, H-4), 3.83–3.70 (m, 3H, H-5 and H-6), 2.33 (s, 3H, CH_3_). ^13^C NMR (CD_3_OD, 100 MHz): *δ* 143.4, 138.8, 140.7, 133.9, 133.1, 131.6, 130.7, 128.7, 126.2, 125.5, 121.2, 91.7, 80.9, 69.5, 69.2, 67.9, 62.3, 21.1. HRMS calculated for [C_19_H_22_N_3_O_4_S_2_]^+^, 420.1052; found: 420.1052.

#### 3.3.4 *p*-Methylphenyl 3-deoxy-3-[4-(thien-3-yl)-1*H*-1,2,3-triazol-1-yl]-1-thio-β-d-galactopyranoside (7)

*m* = 30 mg, x = 3-ethynylthiophene, *y* = 1, *t* = 21 h. Yield 31 mg, 77%. [*α*]^20^_D_ 62.5 (c 75, CH_3_OH). ^1^H NMR (CD_3_OD, 400 MHz): *δ* 8.31 (s, 1H, Ph), 7.75 (m, 1H, Ph), 7.50 (m, 4H, Ph), 7.15 (d, *J* = 8.1 Hz, 2H, Ph), 7.09 (dd, *J* = 5.1, 3.6 Hz, 1H, Ph), 4.86 (dd, *J* = 10.5, 3.0 Hz, 1H, H-3), 4.78 (d, *J* = 9.5 Hz, 1H, H-1), 4.28 (t, *J* = 10.0 Hz, 1H, H-2), 4.14 (d, *J* = 2.9 Hz, 1H, H-4), 3.84–3.70 (m, 3H, H-5 and H-6), 2.32 (s, 3H, CH_3_). ^13^C NMR (CD_3_OD, 100 MHz): *δ* 144.6, 138.7, 140.7, 133.1, 133.0, 131.6, 130.7, 127.6, 126.7, 122.0, 121.6, 91.7, 80.9, 69.6, 69.1, 68.0, 62.3, 21.1. HRMS calculated for [C_19_H_22_N_3_O_4_S_2_]^+^, 420.1052; found: 420.1052.

#### 3.3.5 *p*-Methylphenyl 3-deoxy-3-[4-(1*H*-imidazol-2-yl)-1*H*-1,2,3-triazol-1-yl]-1-thio-β-d-galactopyranoside (8)

*m* = 24 mg, x = 2-ethynylimidazole, *y* = 1, *t* = 22 h. Yield 14 mg, 45%. [*α*]^20^_D_ 10.1 (c 0.44, CH_3_OH). ^1^H NMR (CD_3_OD, 400 MHz): *δ* 8.41 (bs, 1H, Ph), 7.50 (d, *J* = 8.2 Hz, 2H, Ph), 7.16 (bs, 1H, Ph), 7.16 (d, *J* = 8.2 Hz, 2H, Ph), 4.88 (obscured by water H-3), 4.77 (d, *J* = 9.5 Hz, 1H, H-1), 4.23 (t, *J* = 10.0 Hz, 1H, H-2), 4.14 (d, *J* = 2.2 Hz, 1H, H-4), 3.83–3.70 (m, 3H, H-5 and H-6), 2.34 (s, 3H, CH_3_). ^13^C NMR (CD_3_OD, 400 MHz): *δ* 138.8, 133.3, 131.5, 130.7, 91.6, 80.9, 69.5, 69.3, 67.9, 62.3, 21.1. HRMS calculated for [C_18_H_22_N_5_O_4_S]^+^, 404.1392; found: 404.1382.

#### 3.3.6 *p*-Methylphenyl 3-deoxy-3-[4-(1*H*-imidazol-4-yl)-1*H*-1,2,3-triazol-1-yl]-1-thio-β-d-galactopyranoside (9)

To a solution of *p*-methylphenyl 3-azido-3-deoxy-1-thio-β-d-galactopyranoside (20 mg, 0.064 mmol), 1-{[2-(trimethylsilyl)ethoxy]methyl}-4-[2-(trimethylsilyl)ethynyl]-1*H*-imidazole (28 mg, 0.096 mmol) and CuI (3.1 mg, 0.016 mmol) in MeCN (3 mL) was added diisopropylethylamine (0.022 mL, 0.128 mmol). The mixture was stirred 72 h at 50 °C before quenching with sat. aq. NH_4_Cl followed by evaporation of the solvent. The residue was filtered through a short silica column (CH_2_Cl_2_ : MeOH 9 : 1) to give an amorphous white solid that was dissolved in CH_2_Cl_2_ (4 mL) and cooled to 0 °C. Boron trifluoride diethyl etherate (0.016 mL, 0.129 mmol) was added and the resulting mixture was allowed to reach rt in 20 min and then stirred 3 h at rt. The reaction mixture was quenched with sat. aq. NaHCO_3_, the volatiles were evaporated and the residue purified with column chromatography (CH_2_Cl_2_ : MeOH 9 : 1 to >4 : 1) to give 9 (11 mg, 42%) as an amorphous white solid. [*α*]^20^_D_ 77.0 (c 0.57, CH_3_OH). ^1^H NMR (CD_3_OD, 400 MHz): *δ* 8.73 (bs, 1H, Ph), 8.46 (s, 1H, Ph), 7.82 (bs, 1H, Ph), 7.50 (d, *J* = 8.1 Hz, 2H, Ph), 7.16 (d, *J* = 8.1 Hz, 2H, Ph), 4.92 (obscured by water H-3), 4.78 (d, *J* = 9.5 Hz, 1H, H-1), 4.23 (t, *J* = 10.0 Hz, 1H, H-2), 4.15 (d, *J* = 2.7 Hz, 1H, H-4), 3.84–3.70 (m, 3H, H-5 and H-6), 2.33 (s, 3H, CH_3_). ^13^C NMR (CD_3_OD, 400 MHz): *δ* 138.9, 133.3, 131.4, 130.7, 91.5, 80.9, 69.5, 69.3, 68.0, 62.3, 21.1. HRMS calculated for [C_18_H_22_N_5_O_4_S]^+^, 404.1392; found: 404.1393.

### 3.4 General procedure for preparation of triazoles (12–15)

To a solution of methoxycarbonylmethyl 2,4,6-tri-*O*-acetyl-3-azido-3-deoxy-1-thio-β-d-galactopyranoside 11 (25 mg, 0.060 mmol) and CuI (2.8 mg, 0.015 mmol) in MeCN (3 mL), the corresponding acetylene derivative (x, 1.5 equiv.) and diisopropylethylamine (0.031 mL, 0.180 mmol) were added. The mixture was stirred (*t*) h at 50 °C before quenching with sat. aq. NH_4_Cl followed by evaporation of the solvent. The residue was extracted twice with EtOAc and the organic phases were washed with brine, dried and evaporated. The obtained crude product was dissolved in methanol (5 mL) and NaOMe (1 M, 2 mL) was added and the solution was stirred overnight. After neutralization with Dowex 50W-X8 (H^+^) resin and filtration was the residue and LiOH (5 equiv.) dissolved in THF (2 mL) and water (0.2 mL) and the solution was stirred overnight at rt. After neutralization with Dowex 50W-X8 (H^+^) resin and filtration was the product purified with column chromatography (CH_2_Cl_2_ : MeOH : H_2_O 4 : 1 : 0 to >65 : 35 : 1) to give the product as an amorphous white solid.

#### 3.4.1 Carboxymethyl 3-deoxy-3-[4-(thien-3-yl)-1*H*-1,2,3-triazol-1-yl]-1-thio-β-d-galactopyranoside (12)

x = 3-ethynylthiophene, *t* = 14 h. Yield 17 mg, 73%. [*α*]^20^_D_ 16.2 (c 1.07, CH_3_OH). ^1^H NMR (CD_3_OD, 400 MHz): *δ* 8.33 (s, 1H, Ph), 7.76 (dd, *J* = 2.5, 1.7 Hz, 1H, Ph), 7.51 (m, 2H, Ph), 4.84 (dd, *J* = 10.6, 3.0 Hz, 1H, H-3), 4.74 (d, *J* = 8.7 Hz, 1H, H-1), 4.28 (d, *J* = 10.0 Hz, 1H, H-2), 4.14 (d, *J* = 2.9 Hz, 1H, H-4), 3.84–3.77 (m, 2H, H-5 and H-6), 3.70 (dd, *J* = 10.7, 4.8 Hz, 1H, H-6), 3.63 (d, *J* = 15.2 Hz, 1H, CH_2_), 3.50 (d, *J* = 15.2 Hz, 1H, CH_2_). ^13^C NMR (CD_3_OD, 400 MHz): *δ* 174.5, 144.6, 133.0, 127.6, 126.7, 122.0, 121.7, 87.4, 81.1, 69.7, 68.9, 68.4, 62.3, 32.0. HRMS calculated for [C_14_H_18_N_3_O_6_S_2_]^+^, 388.0637; found: 388.0637.

#### 3.4.2 Carboxymethyl 3-deoxy-3-[4-(thiazol-2-yl)-1*H*-1,2,3-triazol-1-yl]-1-thio-β-d-galactopyranoside (13)

x = thiazol-2-ylethynyltrimethylsilane, *t* = 21 h. Yield 11 mg, 48%. [*α*]^20^_D_ 17.6 (c 0.59, CH_3_OH). ^1^H NMR (CD_3_OD, 400 MHz): *δ* 8.55 (s, 1H, Ph), 7.88 (d, *J* = 3.3 Hz, 1H, Ph), 7.63 (d, *J* = 3.3 Hz, 1H, Ph), 4.91 (dd, *J* = 10.6, 3.0 Hz, 1H, H-3), 4.72 (d, *J* = 9.4 Hz, 1H, H-1), 4.27 (dd, *J* = 10.6, 9.4 Hz, 1H, H-2), 4.15 (d, *J* = 3.0 Hz, 1H, H-4), 3.85–3.76 (m, 2H, H-5 and H-6), 3.70 (dd, *J* = 11.0, 5.2 Hz, 1H, H-6), 3.59 (d, *J* = 15.2 Hz, 1H, CH_2_), 3.51 (d, *J* = 15.2 Hz, 1H, CH_2_). ^13^C NMR (CD_3_OD, 400 MHz): *δ* 161.0, 144.1, 143.3, 122.9, 120.7, 87.4, 81.1, 69.7, 69.1, 68.3, 62.3, 32.3. HRMS calculated for [C_13_H_17_N_4_O_6_S_2_]^+^, 389.0590; found: 389.0586.

#### 3.4.3 Carboxymethyl 3-deoxy-3-[4-(thiazol-4-yl)-1*H*-1,2,3-triazol-1-yl]-1-thio-β-d-galactopyranoside (14)

x = thiazol-4-ylethynyltrimethylsilane, *t* = 26 h. Yield 11 mg, 48%. [*α*]^20^_D_ 18.8 (c 0.80, CH_3_OH). ^1^H NMR (CD_3_OD, 400 MHz): *δ* 9.18 (bs, 1H, Ph), 8.46 (s, 1H, Ph), 8.02 (bs, 1H, Ph), 4.89 (obscured by water H-3), 4.73 (d, *J* = 8.3 Hz, 1H, H-1), 4.28 (t, *J* = 9.9 Hz, 1H, H-2), 4.16 (d, *J* = 2.5 Hz, 1H, H-4), 3.85–3.77 (m, 2H, H-5 and H-6), 3.70 (dd, *J* = 11.0, 5.2 Hz, 1H, H-6), 3.61 (d, *J* = 15.3 Hz, 1H, CH_2_), 3.51 (d, *J* = 15.3 Hz, 1H, CH_2_). ^13^C NMR (CD_3_OD, 400 MHz): *δ* 121.4, 87.5, 81.2, 69.7, 69.1, 68.3, 62.3, 30.9. HRMS calculated for [C_13_H_17_N_4_O_6_S_2_]^+^, 389.0590; found: 389.0593.

#### 3.4.4 Carboxymethyl 3-deoxy-3-[4-(thiazol-5-yl)-1*H*-1,2,3-triazol-1-yl]-1-thio-β-d-galactopyranoside (15)

x = thiazol-5-ylethynyltrimethylsilane, *t* = 25 h. Yield 7 mg, 30%. [*α*]^20^_D_ 5.3 (c 0.94, (CD_3_)_2_SO). ^1^H NMR ((CD_3_)_2_SO, 400 MHz): *δ* 9.08 (s, 1H, Ph), 8.61 (s, 1H, Ph), 8.28 (s, 1H, Ph), 5.31 (bs, 1H, OH-4), 4.83 (dd, *J* = 10.5, 3.0 Hz, 1H, H-3), 4.64 (d, *J* = 9.3 Hz, 1H, H-1), 4.08 (t, *J* = 9.9 Hz, 1H, H-2), 3.93 (s, 1H, H-4), 3.70 (t, *J* = 6.3 Hz, 1H, H-5), 3.55–3.40 (m, 4H, H-6 and CH_2_). ^13^C NMR ((CD_3_)_2_SO, 100 MHz): *δ* 153.2, 139.5, 138.0, 121.4, 85.3, 79.3, 67.5, 67.1, 66.7, 60.2, 31.1. HRMS calculated for [C_13_H_17_N_4_O_6_S_2_]^+^, 389.0590; found: 389.0594.

### 3.5 General procedure for preparation of triazoles (19–21)

To a solution of 1,1′-sulfanediyl-bis-(2,4,6-tri-*O*-acetyl-3-azido-3-deoxy-β-d-galactopyranoside) 18 (*m*) and CuI (0.25 equiv.) in DMF (3 mL), the corresponding acetylene derivative (x, 1.5 equiv./azide) and diisopropylethylamine (2 equiv./acetylene derivative) were added. The mixture was stirred (*t*) h at 50 °C before quenching with sat. aq. NH_4_Cl followed by evaporation of the solvent. The residue was extracted twice with EtOAc and the organic phases were washed with brine, dried and evaporated. The obtained crude product was dissolved in MeOH (3 mL) and NaOMe (1 M, 1 mL) was added and the solution was stirred overnight at rt. After neutralization with Dowex 50W-X8 (H^+^) resin and filtration the product was purified with either column chromatography (CH_2_Cl_2_ : MeOH 9 : 1) or preparative HPLC to give the product as an amorphous white solid.

#### 3.5.1 1,1′-Sulfanediyl-bis-{3-deoxy-3-[4-(thiazol-2-yl)-1*H*-1,2,3-triazol-1-yl]-β-d-galactopyranoside} (19)

*m* = 40 mg, x = thiazol-2-ylethynyltrimethylsilane, *t* = 24 h. Yield 21 mg, 55%. [*α*]^20^_D_ 4.4 (c 0.23, CH_3_OH). ^1^H NMR ((CD_3_)_2_SO, 400 MHz): *δ* 8.52 (s, 2H, Ph), 7.92 (d, *J* = 3.3 Hz, 2H, Ph), 7.77 (d, *J* = 3.3 Hz, 2H, Ph), 5.54 (d, *J* = 6.9 Hz, 2H, OH-2), 5.35 (d, *J* = 7.4 Hz, 2H, OH-4), 4.95 (m, 4H, H-1 and H-3), 4.76 (s, 2H, OH-6), 4.24 (m, 2H, H-2), 3.99 (m, 2H, H-4), 3.75 (t, *J* = 6.3 Hz, 2H, H-5), 3.55 (m, 4H, H-6). ^13^C NMR ((CD_3_)_2_SO, 100 MHz): *δ* 158.9, 143.5, 141.6, 121.6, 119.8, 83.4, 79.3, 67.6, 67.3, 66.7, 60.1. HRMS calculated for [C_22_H_27_N_8_O_8_S_3_]^+^, 627.1114; found: 627.1109.

#### 3.5.2 1,1′-Sulfanediyl-bis-{3-deoxy-3-[4-(1*H*-imidazol-2-yl)-1*H*-1,2,3-triazol-1-yl]-β-d-galactopyranoside} (20)

*m* = 30 mg, x = 2-ethynylimidazole, *t* = 60 h. Yield 8 mg, 30%. [*α*]^20^_D_ 3.5 (c 0.57, CH_3_OH). ^1^H NMR (CD_3_OD, 400 MHz): *δ* 8.63 (s, 2H, Ph), 7.10 (bs, 4H, Ph), 5.05 (t, *J* = 10.1 Hz, 2H, H-2), 4.92 (dd, *J* = 10.8, 2.8 Hz, 2H, H-3), 4.78 (d, *J* = 9.5 Hz, 2H, H-1), 4.14 (d, *J* = 2.8 Hz, 2H, H-4), 3.87–3.77 (m, 4H, H-5 and H-6), 3.68 (dd, *J* = 11.3, 4.2 Hz, 2H, H-6). ^13^C NMR (CD_3_OD, 100 MHz): *δ* 140.7, 140.2, 122.9, 87.1, 81.5, 69.5, 68.6, 68.2, 62.8. HRMS calculated for [C_22_H_29_N_10_O_8_S]^+^, 593.1891; found: 593.1893.

#### 3.5.3 3′-Azido-3,3′-dideoxy-3-[4-(thiazol-2-yl)-1*H*-1,2,3-triazol-1-yl]-1,1′-sulfanediyl-di-β-d-galactopyranoside (21)

*m* = 100 mg, x = thiazol-2-ylethynyltrimethylsilane, *t* = 18 h. Yield 24 mg, 31%. [*α*]^20^_D_ 15.8 (c 1.07, CH_3_OH). ^1^H NMR (CD_3_OD, 400 MHz) *δ*: 8.58 (s, 1H, Ph), 7.88 (d, *J* = 3.3 Hz, 1H, Ph), 7.63 (d, *J* = 3.3 Hz, 1H, Ph), 4.93 (m, 2H, H-1 and H-3), 4.77 (d, *J* = 9.7 Hz, 1H, H-1′), 4.44 (t, *J* = 10.1 Hz, 1H, H-2), 4.14 (d, *J* = 3.0 Hz, 1H, H-4), 4.00 (t, *J* = 9.9 Hz, 1H, H-2′), 3.98 (d, *J* = 3.0 Hz, 1H, H-4′), 3.88–3.60 (m, 6H, H-5 and H-6), 3.38 (dd, *J* = 9.9, 3.0 Hz, 1H, H-3′). ^13^C NMR (CD_3_OD, 100 MHz) *δ*: 161.0, 144.1, 143.2, 122.9, 120.7, 86.2, 85.7, 81.3, 81.2, 70.0, 69.8, 69.6, 69.1, 68.5, 68.3, 62.8, 62.6. HRMS calculated for [C_17_H_24_N_7_O_8_S_2_]^+^ 518.1128; found 518.1133.

### 3.6 General procedure for preparation of triazoles (22–23)

To a solution of 3′-azido-3,3′-dideoxy-3-[4-(thiazol-2-yl)-1*H*-1,2,3-triazol-1-yl]-1,1′-sulfanediyl-di-β-d-galactopyranoside 21 (*m*) and CuI (0.25 equiv.) in DMF (3 mL), the corresponding acetylene derivative (x, 1.5 equiv.) and diisopropylethylamine (1 equiv.) were added. The mixture was stirred (*t*) h at 50 °C before quenching with sat. aq. NH_4_Cl followed by evaporation of the solvent. The residue was purified with column chromatography (CH_2_Cl_2_ : MeOH 9 : 1) to give the product as an amorphous white solid.

#### 3.6.1 3,3′-Dideoxy-3-[4-(thiazol-2-yl)-1*H*-1,2,3-triazol-1-yl]-3′-[4-(thien-3-yl)-1*H*-1,2,3-triazol-1-yl]-1,1′-sulfanediyl-di-β-d-galactopyranoside (22)

*m* = 8 mg, x = 3-ethynylthiophene, *t* = 20 h. Yield 6.6 mg, 70%. [*α*]^20^_D_ 2.7 (c 0.56, CH_3_OH). ^1^H-NMR (CD_3_OD, 400 MHz) *δ* 8.67 (s, 1H, Ph), 8.48 (s, 1H, Ph), 7.86 (d, *J* = 3.3 Hz, 1H, Ph), 7.73 (dd, *J* = 2.4, 1.8 Hz, 1H, Ph), 7.63 (d, *J* = 3.3 Hz, 1H, Ph), 7.49 (m, 2H, Ph), 4.97 (dd, *J* = 10.2, 2.7 Hz, 1H, H-3), 4.92–4.82 (obscured by water, 5H, H-1, H-2 and H-3), 4.16 (bs, 2H, H-4), 3.91–3.79 (m, 4H, H-6), 3.72 (t, *J* = 4.4 Hz, 1H, H-5), 3.69 (t, *J* = 4.4 Hz, 1H, H-5). ^13^C NMR (CD_3_OD, 100 MHz): *δ* 144.6, 144.0, 143.2, 133.1, 127.6, 126.7, 122.9, 121.9, 121.8, 120.7, 86.9, 86.8, 81.4, 69.6, 69.0, 68.6, 68.4, 68.2, 62.8. HRMS calculated for [C_23_H_28_N_7_O_8_S_3_]^+^, 626.1161; found: 626.1155.

#### 3.6.2 3,3′-Dideoxy-3′-[4-(3,4-difluorophenyl)-1*H*-1,2,3-triazol-1-yl]-3-[4-(thiazol-2-yl)-1*H*-1,2,3-triazol-1-yl]-1,1′-sulfanediyl-di-β-d-galactopyranoside (23)

*m* = 13 mg, x = 3,4-difluorophenylacetylene, *t* = 17 h. Yield 12 mg, 73%. [*α*]^20^_D_ 3.4 (c 0.87, CH_3_OH). ^1^H NMR (CD_3_OD, 400 MHz) *δ* 8.67 (s, 1H, Ph), 8.58 (s, 1H, Ph), 7.87 (bs, 1H, Ph), 7.74 (ddd, *J* = 11.5, 7.7, 2.2 Hz, 2H, Ph), 7.62 (m, 2H, Ph), 7.31 (dt, *J* = 10.5, 8.5 Hz, 1H, Ph), 4.98–4.83 (m, 6H, H-1, H-2 and H-3), 4.16 (t, *J* = 2.6 Hz, 2H, H-4), 3.91–3.80 (m, 4H, H-6), 3.72 (t, *J* = 3.6 Hz, 1H, H-5), 3.69 (t, *J* = 3.6 Hz, 1H, H-5). ^13^C NMR (CD_3_OD, 100 MHz): *δ* 146.4, 144.0, 143.2, 123.1, 122.9, 122.3, 120.7, 119.1, 118.9, 115.5, 115.3, 86.9, 86.7, 81.4, 69.7, 69.6, 69.0, 68.7, 68.4, 68.2, 62.8. HRMS calculated for [C_25_H_28_F_2_N_7_O_8_S_2_]^+^, 656.1409; found: 656.1423.

### 3.7 Procedures for preparation of probe molecule (25) *via* (24)

#### 3.7.1 3′-Amino-3,3′-dideoxy-3-[4-(thiazol-2-yl)-1*H*-1,2,3-triazol-1-yl]-1,1′-sulfanediyl-di-β-d-galactopyranoside (24)

To a solution of compound 21 (10 mg, 0.019 mmol) in MeOH (2 mL) was added 1,3-propanedithiol (0.008 mL, 0.076 mmol), followed by Et_3_N (0.011 mL, 0.076 mmol). The resulting mixture was stirred 6 h at rt. The volatiles were evaporated and purification of the obtained residue with preparative HPLC gave 24 (8.9 mg, 95%) as an amorphous white solid. [*α*]^20^_D_ 33.0 (c 0.72, CH_3_OH). ^1^H NMR (CD_3_OD, 400 MHz) *δ*: 8.54 (s, 1H, Ph), 7.89 (d, *J* = 3.3 Hz, 1H, Ph), 7.64 (d, *J* = 3.3 Hz, 1H, Ph), 4.93 (dd, *J* = 10.7, 3.0 Hz, 1H, H-3), 4.91 (obscured by water H-1), 4.76 (d, *J* = 9.7 Hz, 1H, H-1′), 4.45 (t, *J* = 10.1 Hz, 1H, H-2), 4.15 (d, *J* = 3.0 Hz, 1H, H-4), 4.03 (d, *J* = 3.0 Hz, 1H, H-4′), 3.88–3.65 (m, 7H, H-2′, H-5, H-5′, H-6 and H-6′), 3.13 (dd, *J* = 10.0, 3.0 Hz, 1H, H-3′). ^13^C NMR (CD_3_OD, 100 MHz) *δ*: 170.3, 161.1, 144.1, 143.3, 122.7, 120.8, 103.6, 86.2, 85.8, 81.4, 81.2, 69.6, 69.2, 69.1, 68.5, 67.8, 62.6, 62.5, 58.6. HRMS calculated for [C_17_H_26_N_5_O_8_S_2_]^+^ 492.1223; found 492.1223.

#### 3.7.2 3,3′-Dideoxy-3-(fluorescein-5-yl-carbonylamino)-3′-[4-(thiazol-2-yl)-1*H*-1,2,3-triazol-1-yl]-1,1′-sulfanediyl-di-β-d-galactopyranoside (25)

To a solution of 24 (4.0 mg, 0.0081 mmol) and 5-FAM-NHS (4.8 mg, 0.0101 mmol) in DMSO (1.5 mL), diisopropylethylamine (0.004 mL, 0.024 mmol) was added and the mixture was stirred 40 h at rt. Purification with preparative HPLC gave 25 (4.2 mg, 61%) as an amorphous yellow solid. [*α*]^20^_D_ 13.7 (c 0.51, CH_3_OH). ^1^H NMR (CD_3_OD, 500 MHz) *δ*: 8.59 (s, 1H, Ph), 8.52 (d, *J* = 1.5 Hz, 1H, Ph), 8.22 (dd, *J* = 8.0, 1.5 Hz, 1H, Ph), 7.89 (d, *J* = 3.3 Hz, 1H, Ph), 7.63 (d, *J* = 3.3 Hz, 1H, Ph), 7.32 (dd, *J* = 8.0, 0.4 Hz, 1H, Ph), 6.71–6.68 (m, 4H, Ph), 6.56 (dd, *J* = 8.8, 2.3 Hz, 2H, Ph), 4.95 (m, 2H, H-1′ and H-3′), 4.87 (obscured by water H-1), 4.49 (t, *J* = 10.0 Hz, 1H, H-2′), 4.23 (dd, *J* = 10.4, 3.0 Hz, 1H, H-3), 4.16 (d, *J* = 2.9 Hz, 1H, H-4′), 4.10 (d, *J* = 2.9 Hz, 1H, H-4), 4.04 (t, *J* = 10.0 Hz, 1H, H-2), 3.90–3.76 (m, 4H, H-5, H-5′, H-6 and H-6′), 3.73–3.70 (m, 2H, H-6 and H-6′). ^13^C NMR (CD_3_OD, 125 MHz) *δ*: 169.0, 161.0, 144.1, 143.3, 138.0, 130.6, 122.9, 120.7, 103.7, 86.4, 81.7, 81.4, 69.7, 69.3, 69.1, 68.5, 63.0, 62.6, 40.4. HRMS calculated for [C_38_H_36_N_5_O_14_S_2_]^+^ 850.1700; found 850.1708.

### 3.8 Direct binding fluorescence polarization experiments determining probe properties

Human galectin-1 was expressed and purified as earlier described.^31^ A fixed concentration (1 nM) of fluorescein-labeled probe molecule 25 in PBS, was mixed with a range of galectin-1 in dilution from 0.0002–30 μM in PBS in a final volume of 160 μL at rt, in black 96 well plates (FluoroNunc). Polarization was measured using a PheraStarFS plate reader with software PHERAstar Mars version 2.10 R3 (BMG, Offenburg, Germany) and fluorescence anisotropy of fluorescein tagged probes measured with excitation at 485 nm and emission at 520 nm. *K*_d_ and SEM values were determined in GraphPad Prism as previously described.^22^

### 3.9 Competitive fluorescence polarization experiments determining galectin affinities

Human galectin-1 ^31^ and galectin-3 ^32^ were expressed and purified as earlier described. Fluorescence polarization experiments were performed on a PheraStarFS plate reader with software PHERAstar Mars version 2.10 R3 (BMG, Offenburg, Germany) and fluorescence anisotropy of fluorescein tagged probes measured with excitation at 485 nm and emission at 520 nm. *K*_d_ values and SEM were determined in GraphPad Prism as previously described^22,23^ with specific conditions for each galectin as described below. Galectin-1 and galectin-3 experiments for compounds 4–9, 12–15, and 17 were performed as described^13^ and galectin-3 experiments for compounds 1, 19–20, and 22–23 were performed as described.^15^ Galectin-1 experiments for high affinity compounds 1, 19–20, and 22–23 were done at rt with galectin-1 at 50 nM and the fluorescent probe molecule 25 at 1 nM; 100 nM bovine serum albumin was included to prevent significant loss of galectin-1 and the probe at these low concentrations. Inhibitors 4–9, 12–17, and 19–23 were dissolved in neat DMSO at 10–50 mM and diluted in PBS to 3–6 different concentrations to be tested in duplicates. *K*_d_ average and SEM were calculated from 4 to 25 single point measurements from at least two independent experiments showing between 10–90% inhibition.

### 3.10 Co-crystallization of galectin-1 with ligand 1

Compound 1 was prepared in the galectin-1 crystallization conditions by initially solubilizing in 55% w/v polyethylene glycol (PEG 4000 for galectin-1), before addition of other crystallization reagents to give a final concentration of 20 mM of 1 in the galectin crystallization condition (25% w/v PEG 4000, 0.1 M sodium acetate trihydrate, pH 6.2, 0.2 M ammonium sulphate for galectin-1). Apo galectin-1 crystals (prepared as previously described^33^) were soaked in the corresponding ligand-containing crystallization condition for 17–24 h prior to X-ray diffraction experiments.

### 3.11 X-ray diffraction analysis and structure determination

X-ray diffraction data sets were collected at rt from human galectin-1 crystals mounted in 0.7 mm quartz capillaries on a ProteumR (Bruker AXS, Madison, WI, USA) diffractometer with a MacScience M06X^CE^ rotating-anode generator (wavelength 1.5418 Å) equipped with a SMART6000 CCD detector. X-ray diffraction data were integrated using SAINT (Bruker AXS, Madison, WI, USA) and scaled and merged using SCALA^34^ within the CCP4 suite of crystallographic software.^35^ Structures were solved by initial rigid body refinement using a previously determined human galectin-1 structure (3OY8)^33^ with ligand and waters removed, as the initial model. TLS and restrained refinement was performed using REFMAC5 ^36^ with medium NCS restraints for galectin-1 only. Anomalously scattering elements were identified using single wavelength anomalous dispersion log-likelihood gradient maps (SAD LLG maps); calculated using Phaser^37^ (in experimental phasing mode within CCP4) in the ‘SAD with molecular replacement partial structure’ mode with purely anomalous scatterers and zero LLG-map completion cycles using the current model and F+ and F− structure factor amplitudes as input. Visualization of electron density and model building was performed using Coot.^38^ Ligand geometry topologies for refinement were initially created by REFMAC5 within CCP4 (LIBCHECK) or using the Dundee PRODRG2 Server.^39^ In most cases minor to moderate manual editing of the automatically generated topologies was performed to ensure correct atom and bond types. Model validation and analysis was performed using MolProbity.^40^ Figures were created using the CCP4 molecular-graphics project (CCP4MG).^41^

### 3.12 Cell lines and cell culture

The human breast cancer cell line JIMT-1 (ACC589) was purchased from the German Collection of Microorganisms and Cell Cultures (DSMZ) and was routinely maintained in Dulbecco's modified Eagle's medium/nutrient mixture Ham's F12 medium (VWR, Lund, Sweden). The human breast cancer cell lines MCF-7 (HTB-22), HCC1937, and human normal-like breast epithelial cell line MCF-10A (CRL-10317) were obtained from American Type Culture Collection (Manassas, VA, USA) and were cultured in RPMI1640 medium (VWR). The JIMT-1, MCF-7, and HCC1937 cell lines were cultured with the addition of 10% fetal calf serum (FCS) (VWR), nonessential amino acids (1 mM) (VWR), insulin (10 μg mL^−1^) (Sigma-Aldrich), penicillin (100 U mL^−1^) (VWR), and streptomycin (100 μg mL^−1^) (VWR). In addition, HCC1937 cell line was also supplemented with epidermal growth factor (20 ng mL^−1^) (Sigma-Aldrich). The MCF-10A cells were cultured with the addition of 10% heat-inactivated FCS, nonessential amino acids (1 mM), insulin (10 μg mL^−1^), penicillin (100 U mL^−1^), streptomycin (100 μg mL^−1^), epithermal growth factor (20 ng mL^−1^), cholera toxin (50 ng mL^−1^) (Sigma-Aldrich), and hydrocortisol (250 ng mL^−1^) (Sigma-Aldrich). All cell lines were maintained at 37 °C in a humidified incubator with 5% CO_2_.

### 3.13 MTT assay

An MTT assay was used to evaluate the dose response of the compounds as previously described.^42^ Briefly, the compounds were dissolved in DMSO to 20 mM as stock solutions and then serially diluted in PBS and used at final concentrations from 0.04 μM to 40 μM. The final DMSO concentration in the assays was 0.2% for all concentrations used. Accordingly, control was treated with 0.2% DMSO in PBS. Cells were seeded in 96-well plates (5000 cells for JIMT-1, 6000 cells for MCF-7 and HCC1937 cell lines, and 3000 cells for MCF-10A cell line per well in 180 μL medium) and the plates were incubated for 24 h before addition of compound. After 72 h of treatment, MTT solution (20 μL of 5 mg mL^−1^ in PBS) was added to each well and the plate was incubated for 1 h. Thereafter, the medium was removed and the purple formazan product was dissolved by the addition of 100 μL of 100% DMSO per well. The plates were swirled gently for 10 min and the absorbance was monitored at 540 nm using a Labsystems iEMS Reader MF (Labsystems Oy, Helsinki, Finland) and the software DeltaSoft II v.4.14 (Biometallics Inc., Princeton, NJ, USA). The software program GraphPad Prism was used to analyze the data and plot dose response curves. The dose response curve data were from 3 to 5 independent experiments with 6 repetitions for each experiment. The error bars stand for SE.

## 4. Conclusions

The synthesis of thiogalactosides carrying different five-membered heterocycle-triazolyl moieties at C3 revealed that 3-thiophene, 2-thiazole and 2-imidazole heterocycles results in high affinity and selectivity for galectin-1. The structure–activity relationship of thiogalactosides carrying different five-membered heterocycle-triazolyl moieties at C3 revealed a 3-fold decrease in affinity when the heterocycle has a nitrogen or oxygen in the 3-position. Derivatizing the thiodigalactoside scaffold with the heterocycle-triazoles at both galactose C3 carbons resulted in two compounds, the thiophene 1 and the thiazole 19, having single-digit nM affinity for galectin-1 in combination with almost 10-fold selectivity over galectin-3. The structural analysis of thiophene 1 revealed that the five-membered thiophene ring binds deeper in the pocket between Ser29 and Asp123 than six-membered phenyl rings earlier reported as unselective galectin-1/galectin-3 ligands. Hence, implementing the thiazolyl-triazoles on the thiodigalactoside scaffold opens up for alternative five membered (thiazoles 19 and 23) analogs to the thiophene 1, thus advancing the development of galectin-1 ligands.

## Conflicts of interest

F. R. Z. is an employee of and H. L. and U. J. N. are shareholders in Galecto Biotech AB, a company developing galectin inhibitors. The other authors have no conflicts to declare.

## Supplementary Material

RA-008-C8RA04389B-s001
