# Supplementary material for: Aromatic heterocycle galectin-1 interactions for selective single-digit nM affinity ligands
Source: RSC Adv. 2018 Jul 11;8(44):24913–22. doi: 10.1039/c8ra04389b (PMC9082524; doi:10.1039/c8ra04389b)
Supplement: RA-008-C8RA04389B-s001 [file RA-008-C8RA04389B-s001.pdf]

Electronic Supplementary Material (ESI) for RSC Advances.
This journal is © The Royal Society of Chemistry 2018

Supporting Information

Aromatic heterocycle galectin-1 interactions for selective single-digit nM affinity ligands

Kristoffer Peterson,[a] Patrick M. Collins,[b] Xiaoli Huang,[c] Barbro Kahl-Knutsson,[d] Sofia Essén,[a] Fredrik R. Zetterberg,[e] Stina Oredsson,[c] Hakon Leffler,[d] Helen Blanchard[b*] and Ulf J. Nilsson[a*]

[a] Centre for Analysis and Synthesis, Department of Chemistry, Lund University, POB 124, 221 00, Lund, Sweden

[b] Institute for Glycomics, Griffith UniversityGold Coast Campus, Queensland 4222, Australia

[c] Department of Biology, Lund University, POB 118, 221 00, Lund, Sweden

[d] Department of Laboratory Medicine, Section MIG, Lund University BMC-C1228b, Klinikgatan 28, 221 84 Lund, Sweden

[e] Galecto Biotech AB, Sahlgrenska Science Park, Medicinaregatan 8 A, 413 46 Gothenburg, Sweden

*Corresponding author. *Email*: ulf.nilsson@chem.lu.se

Table of Contents

S2-S20	NMR-spectra
S21-S27	Purity chromatograms
S28	Fluorescence polarization titration curves
S29	Dose response curves from the MTT assay
S30-S31	Crystallographic data

NMR spectra of synthesized compounds

***p*-Methylphenyl 3-azido-3-deoxy-1-thio-β-D-galactopyranoside 3**

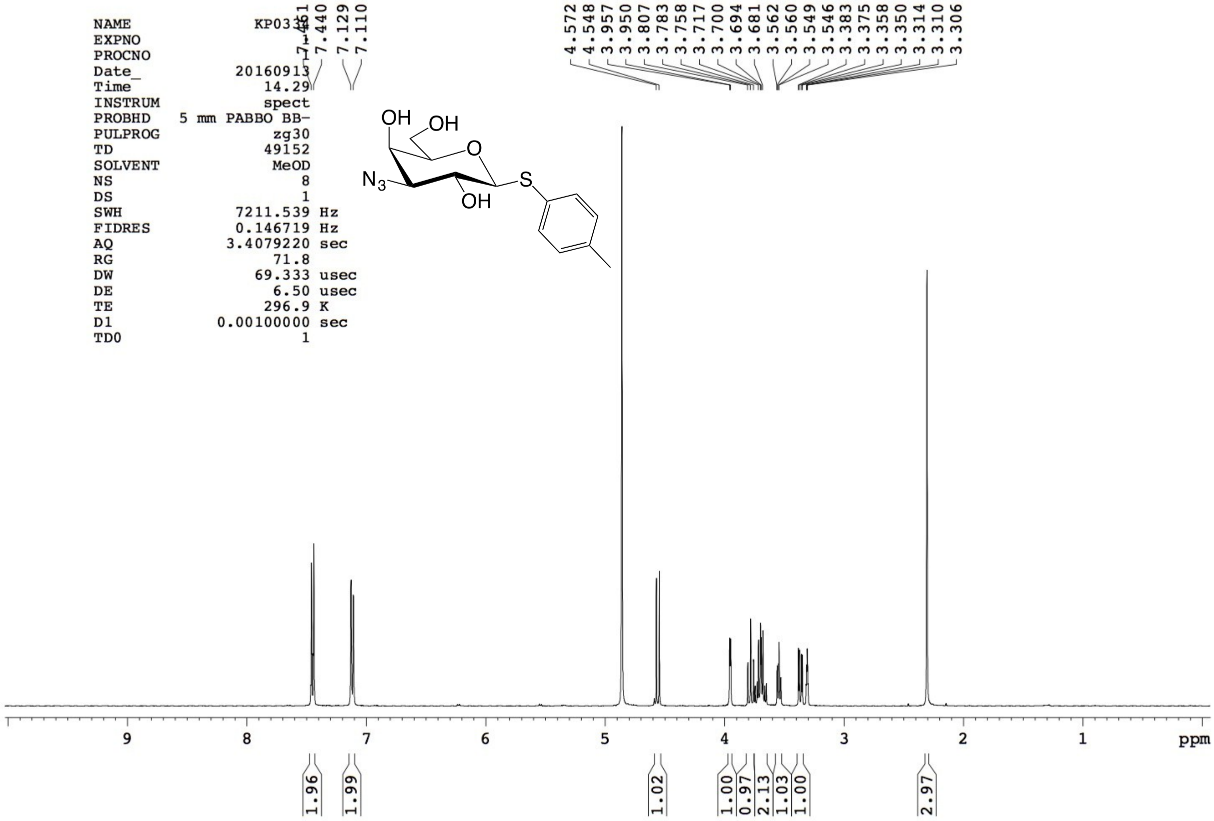

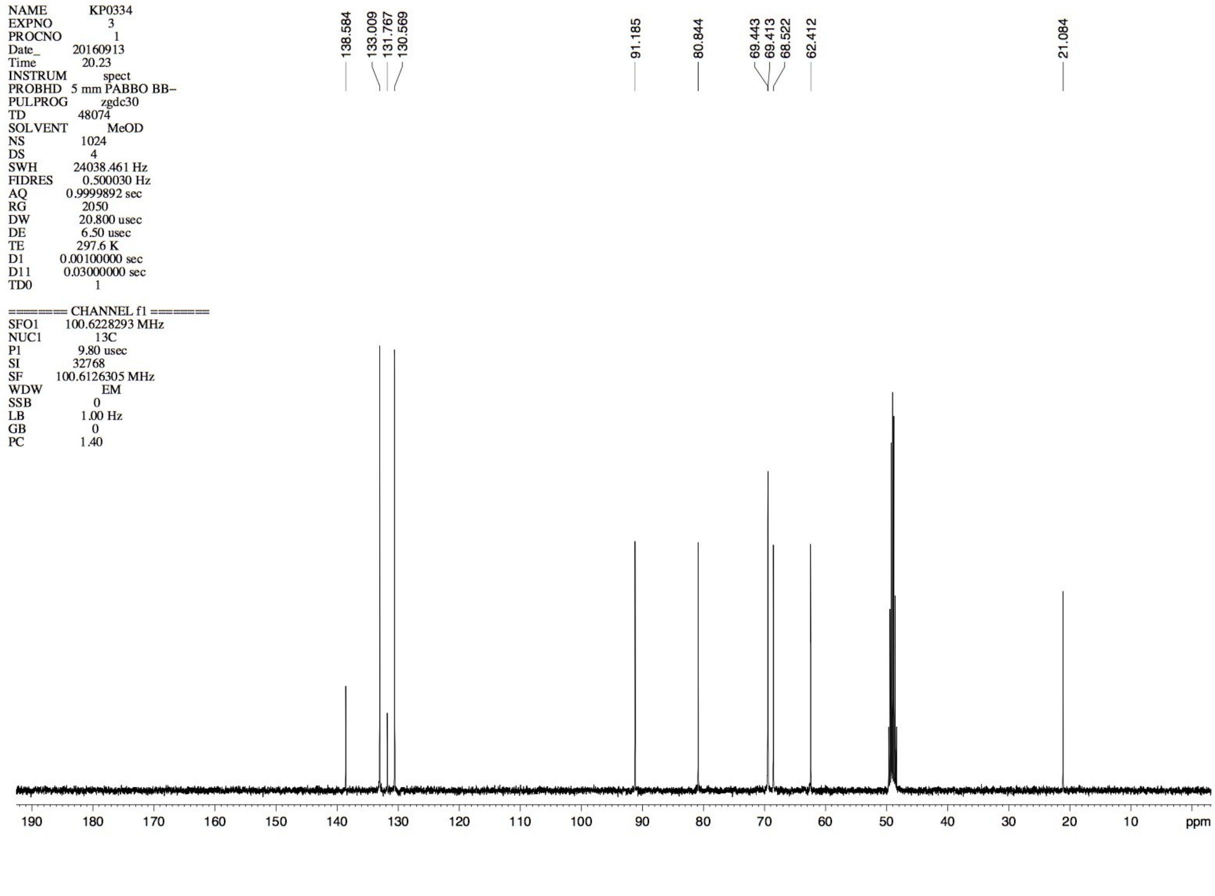

***p*-Methylphenyl 3-deoxy-3-[4-(cyclopentyl)-1*H*-1,2,3-triazol-1-yl]-1-thio-β-D-galactopyranoside 4**

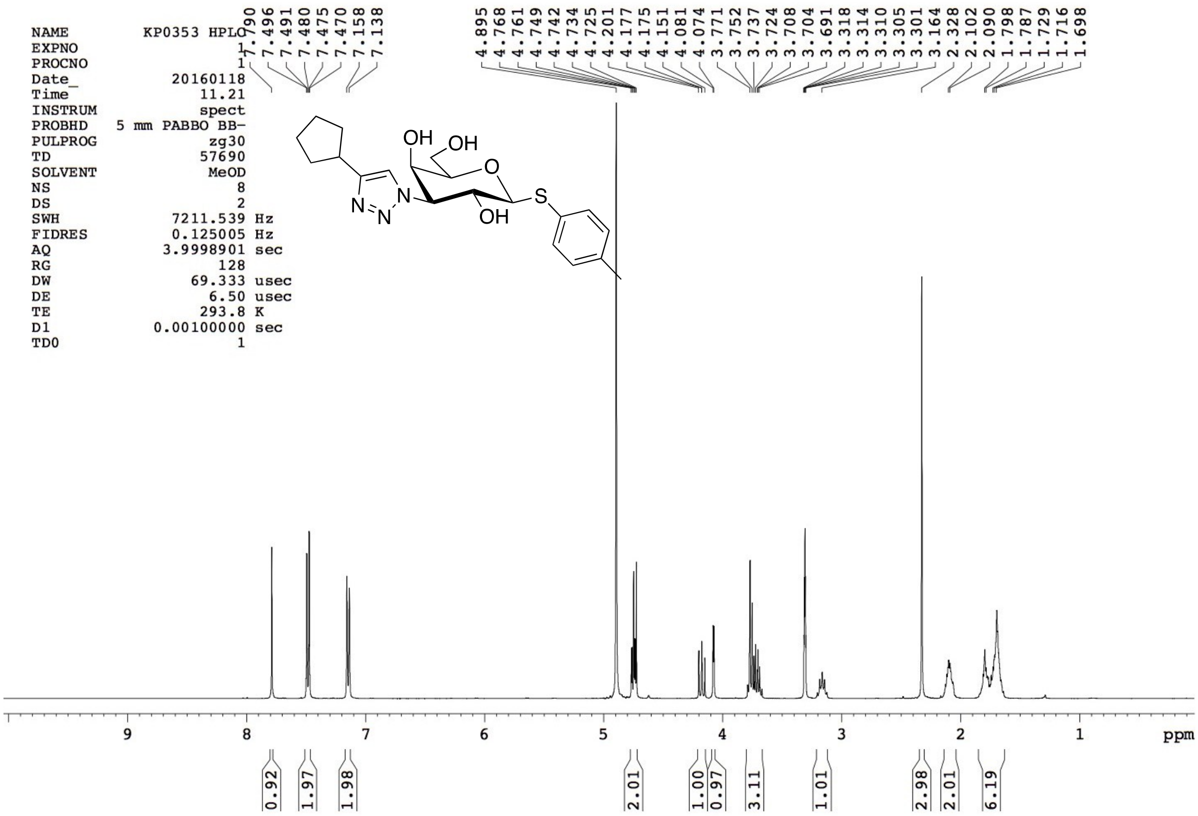

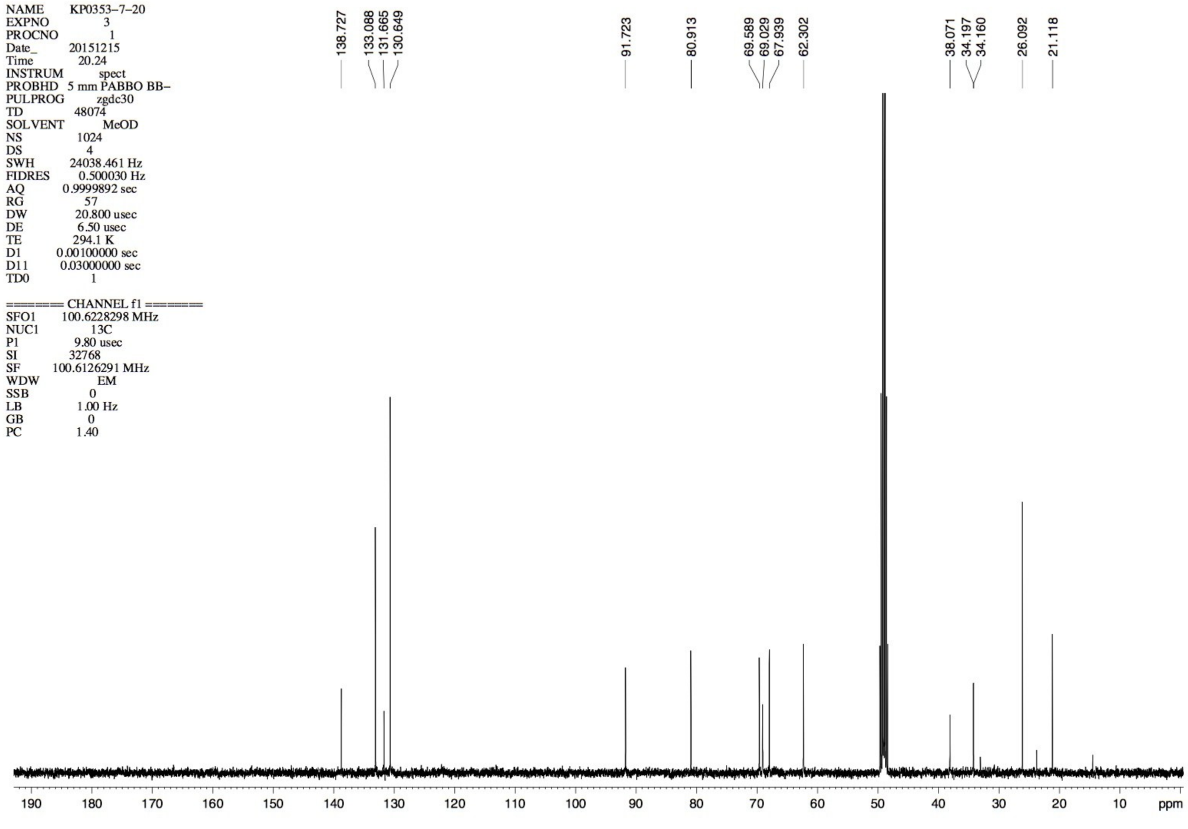

***p*-Methylphenyl 3-deoxy-3-[4-(furan-3-yl)-1*H*-1,2,3-triazol-1-yl]-1-thio-β-D-galactopyranoside 5**

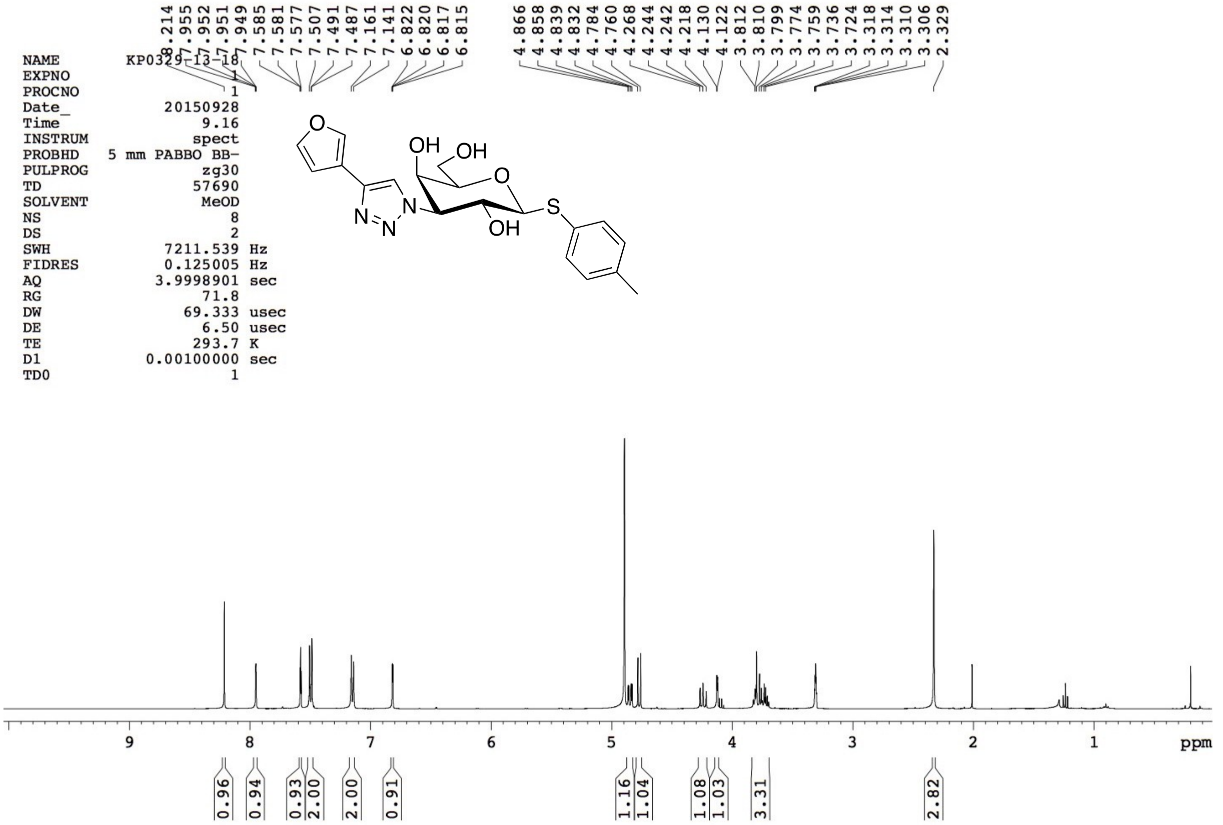

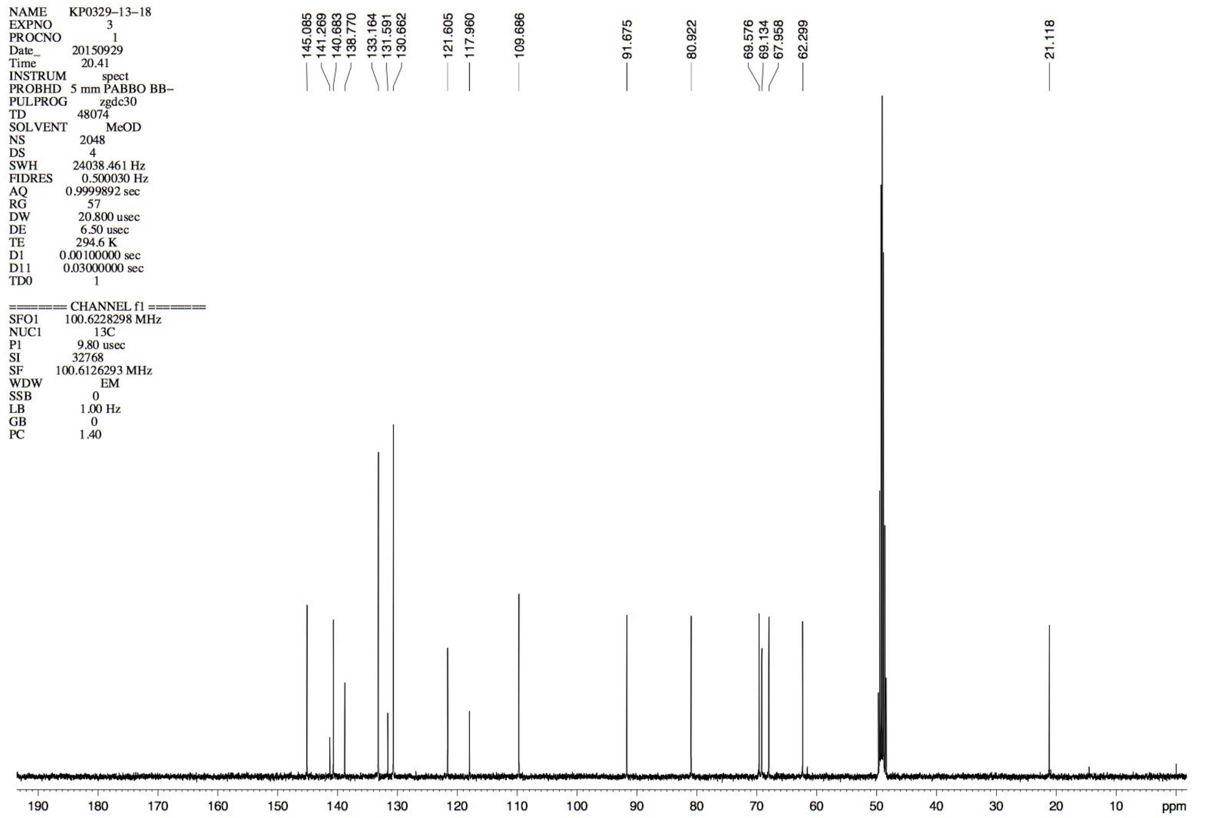

***p*-Methylphenyl 3-deoxy-3-[4-(thien-2-yl)-1*H*-1,2,3-triazol-1-yl]-1-thio-β-D-galactopyranoside 6**

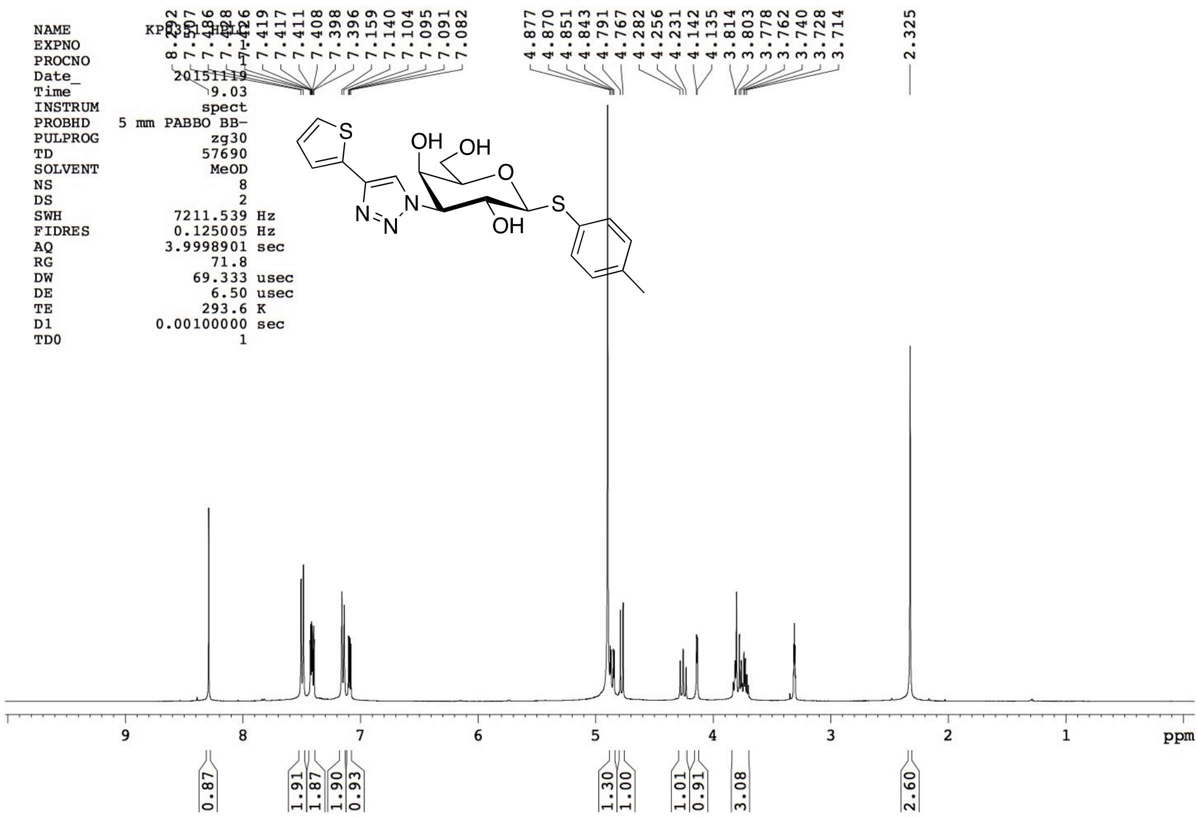

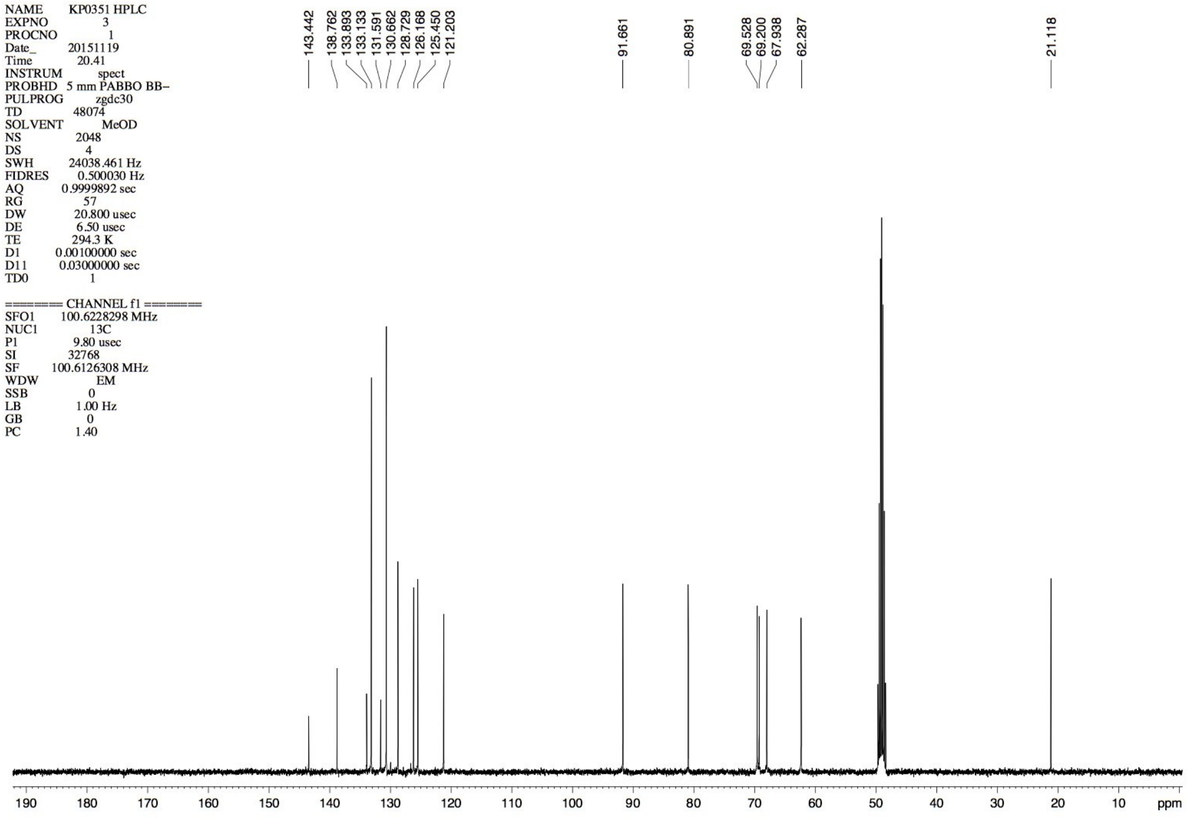

***p*-Methylphenyl 3-deoxy-3-[4-(thien-3-yl)-1*H*-1,2,3-triazol-1-yl]-1-thio-β-D-galactopyranoside 7**

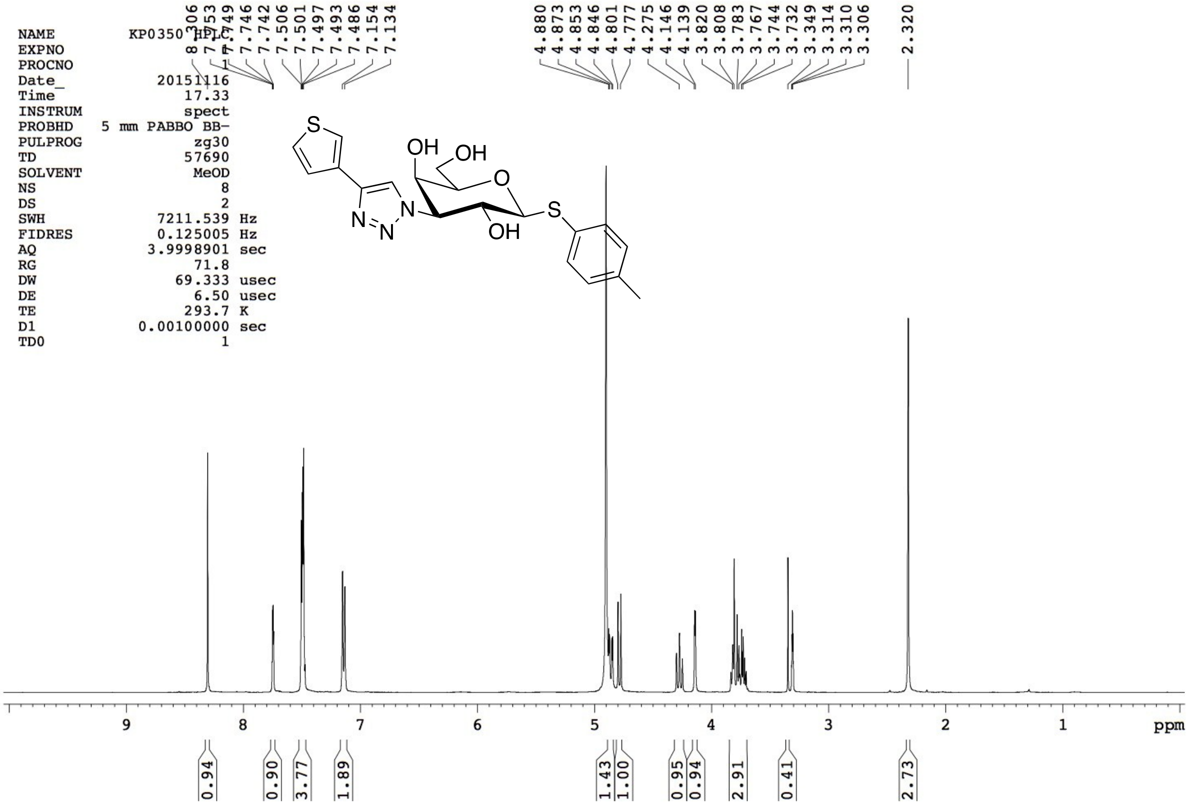

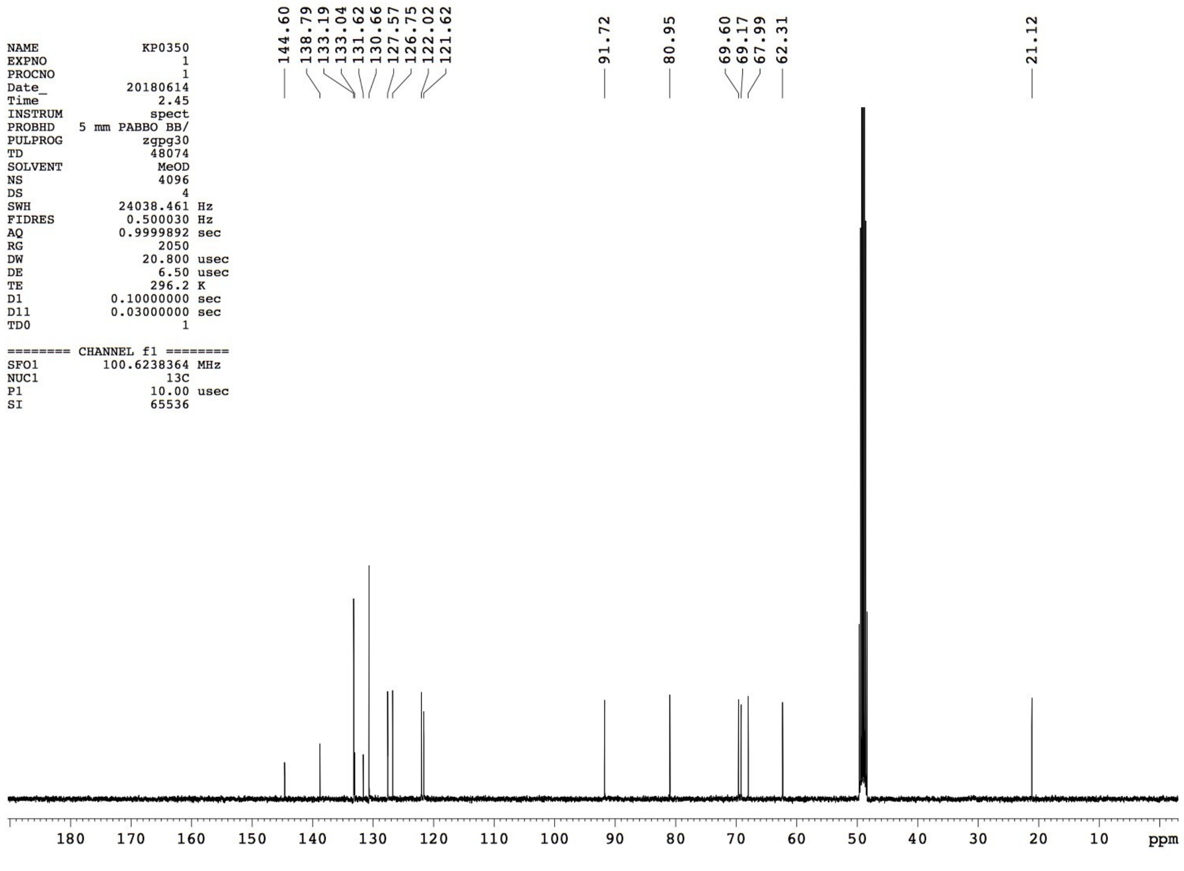

***p*-Methylphenyl 3-deoxy-3-[4-(1*H*-imidazol-2-yl)-1*H*-1,2,3-triazol-1-yl]-1-thio-β-D-galactopyranoside 8**

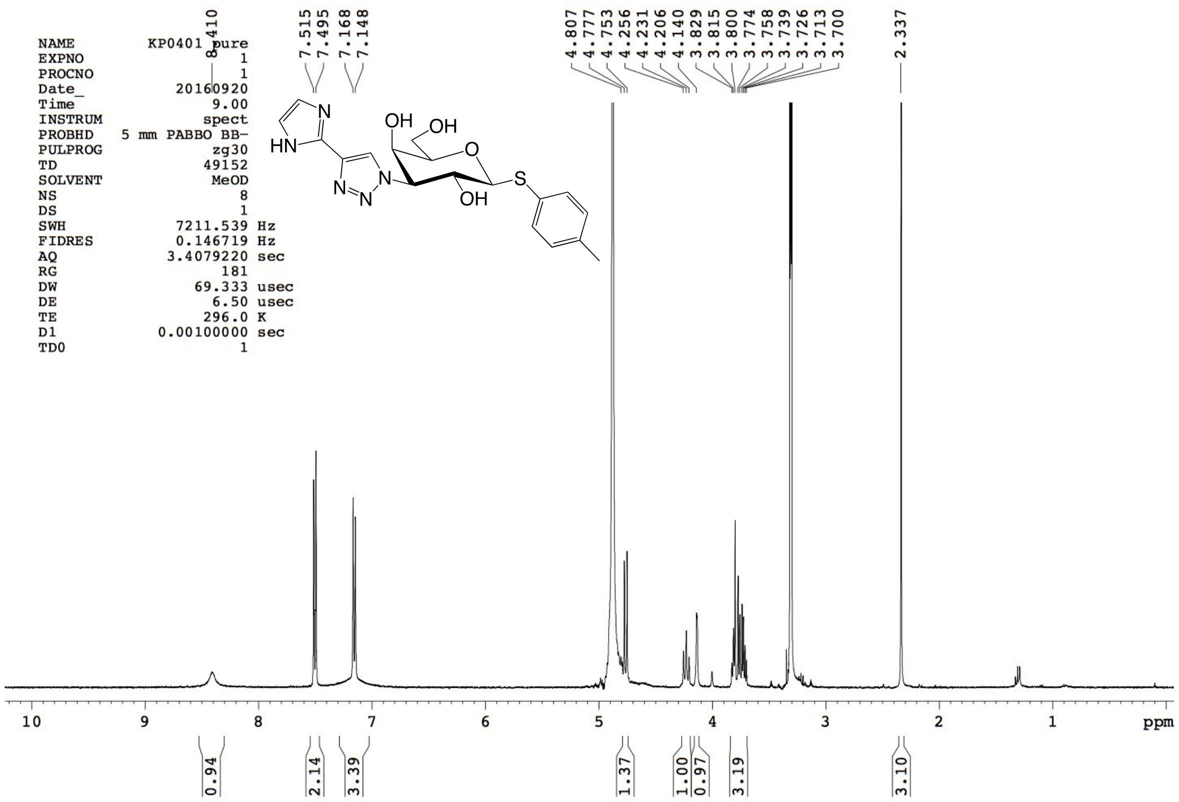

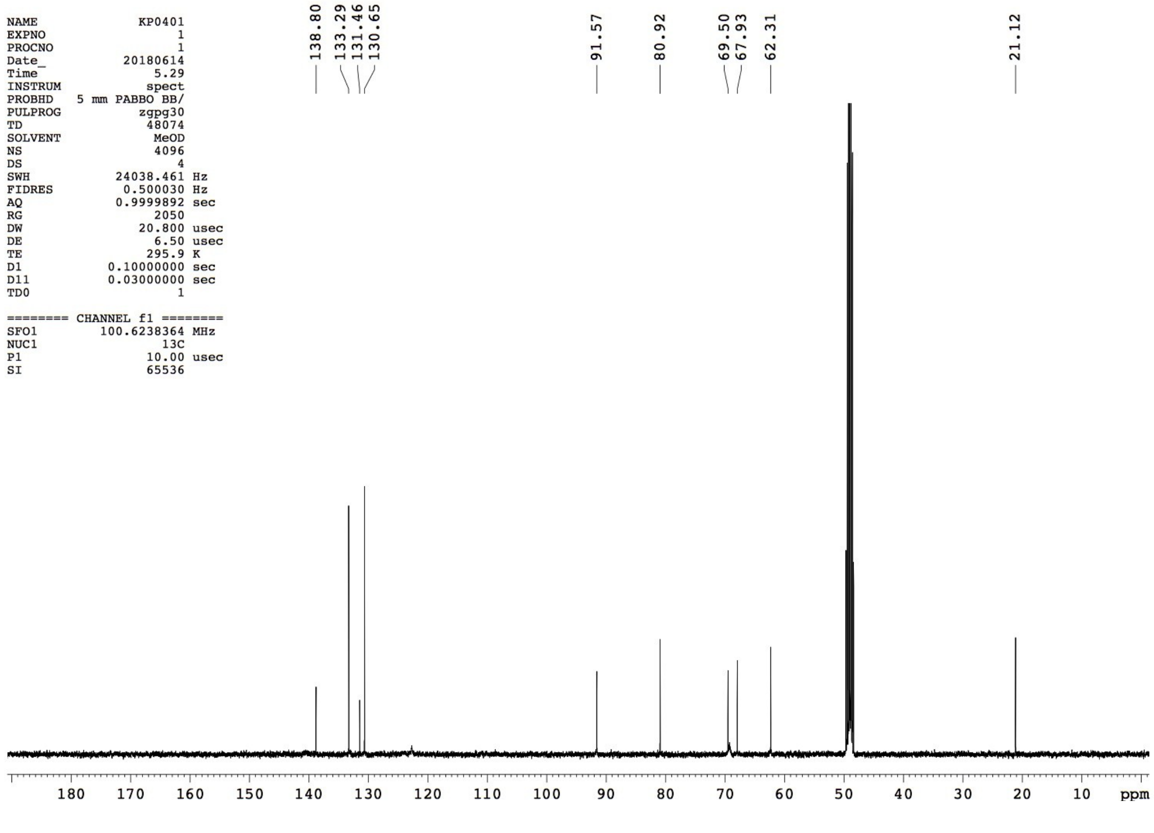

***p*-Methylphenyl 3-deoxy-3-[4-(1*H*-imidazol-4-yl)-1*H*-1,2,3-triazol-1-yl]-1-thio-β-D-galactopyranoside 9**

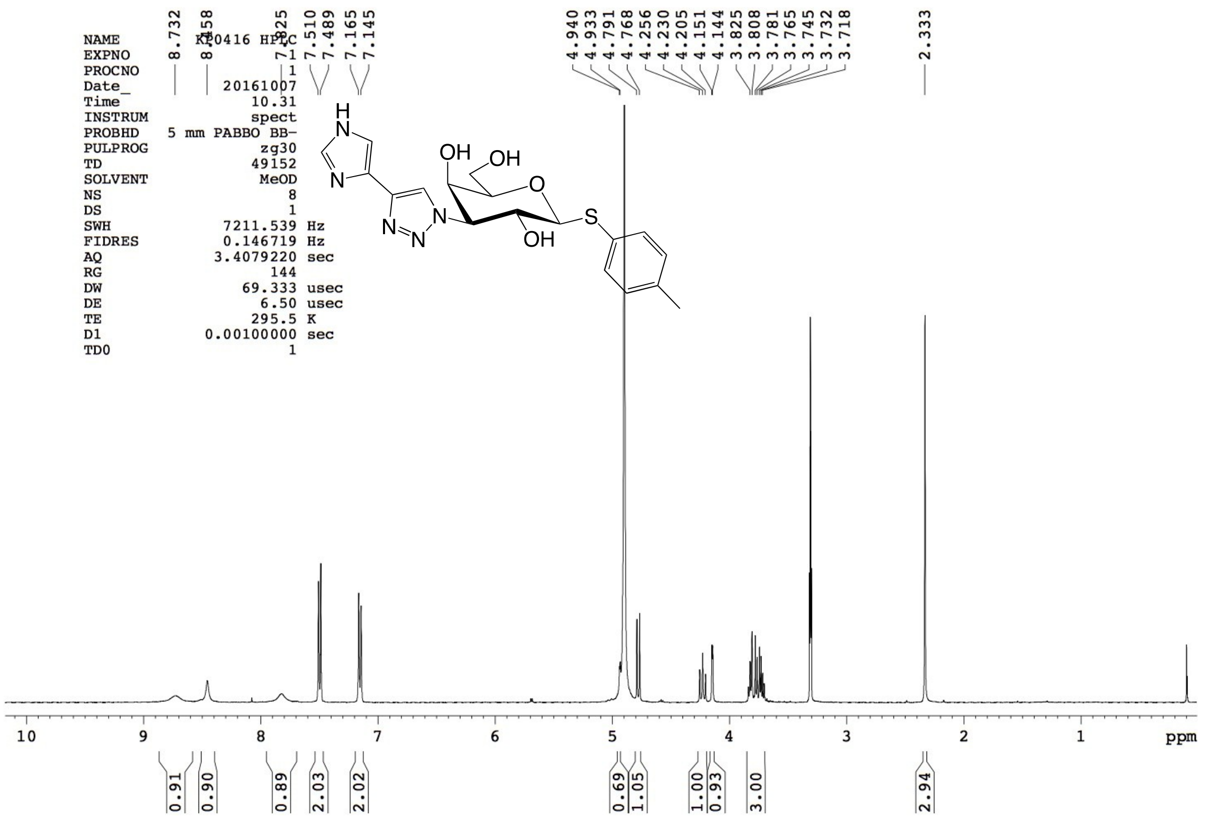

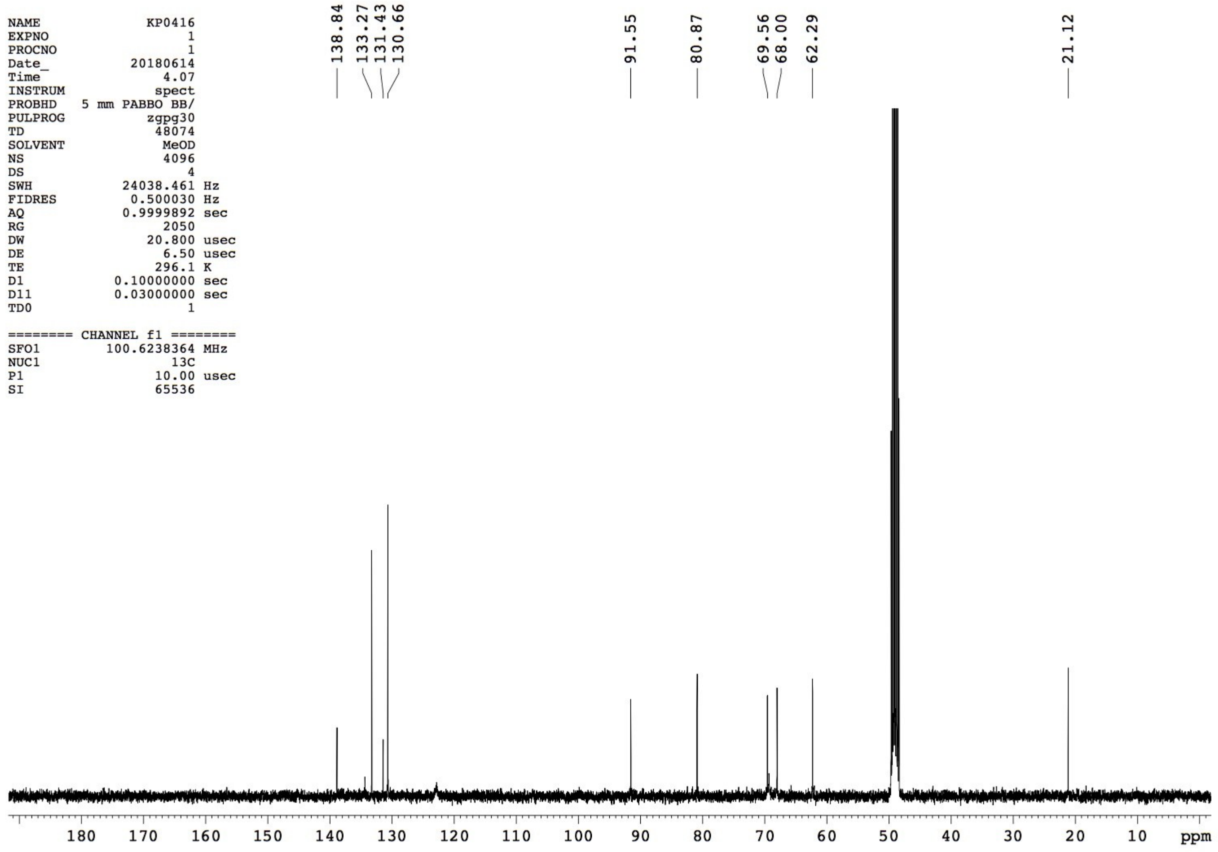

Methyl glycolate-2,4,6-tri-*O*-acetyl-3-azido-3-deoxy-1-thio-β-D-galactopyranoside 11

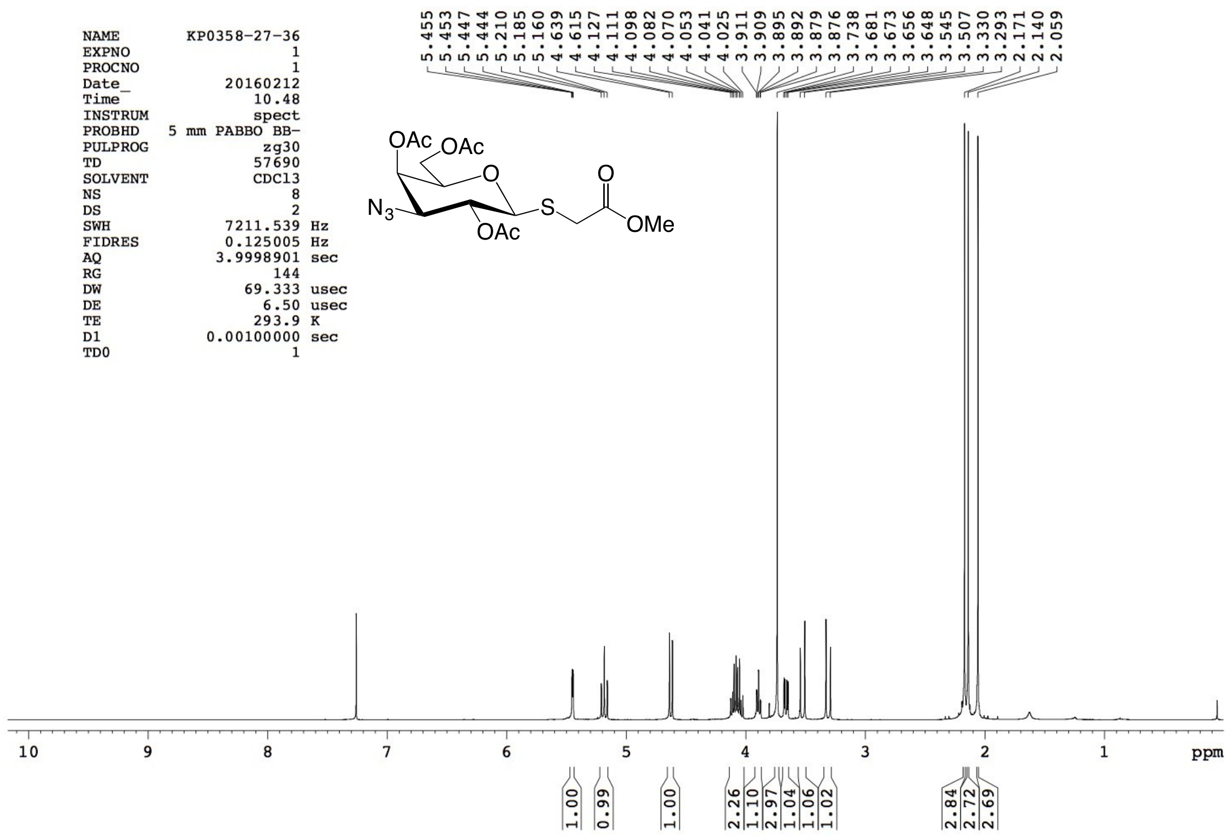

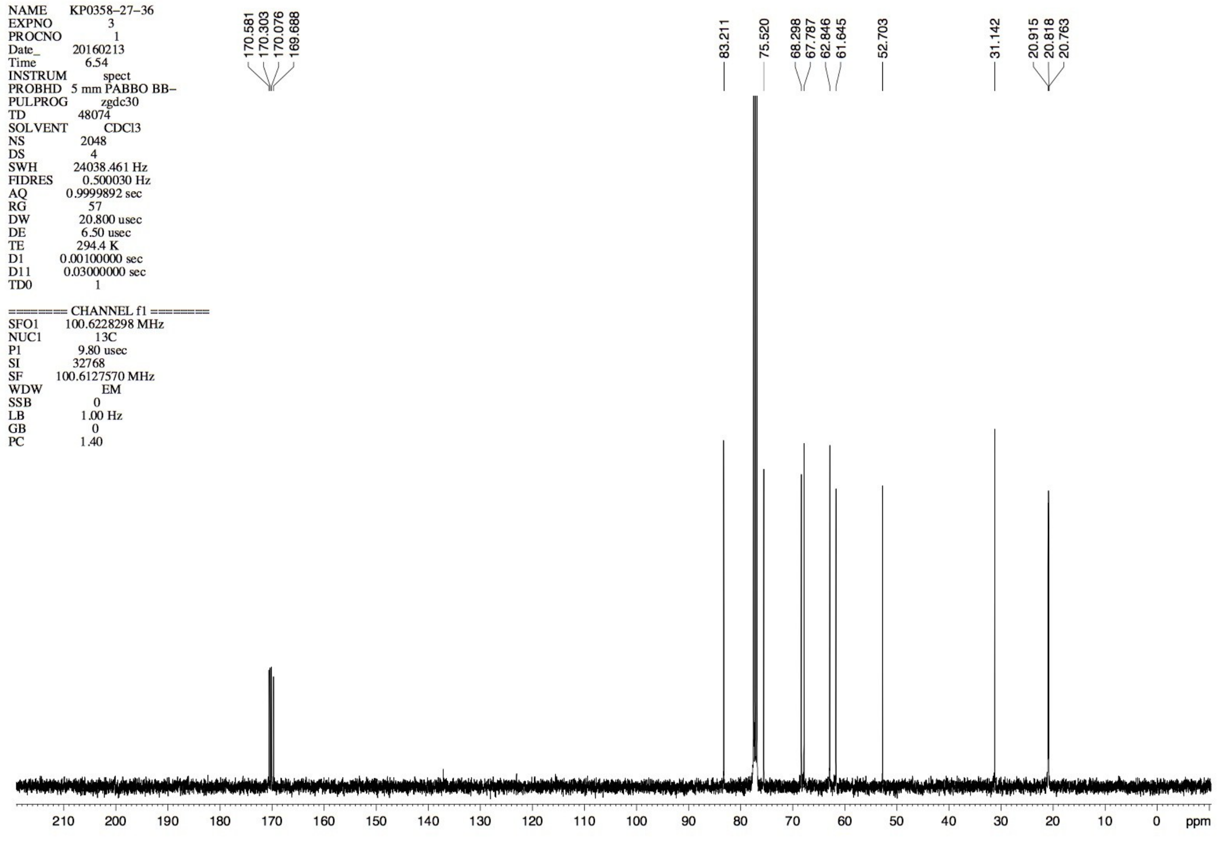

1,3-Dideoxy-3-[4-(thien-3-yl)-1*H*-1,2,3-triazol-1-yl]-1-thiocarboxymethyl-β-D-galactopyranoside 12

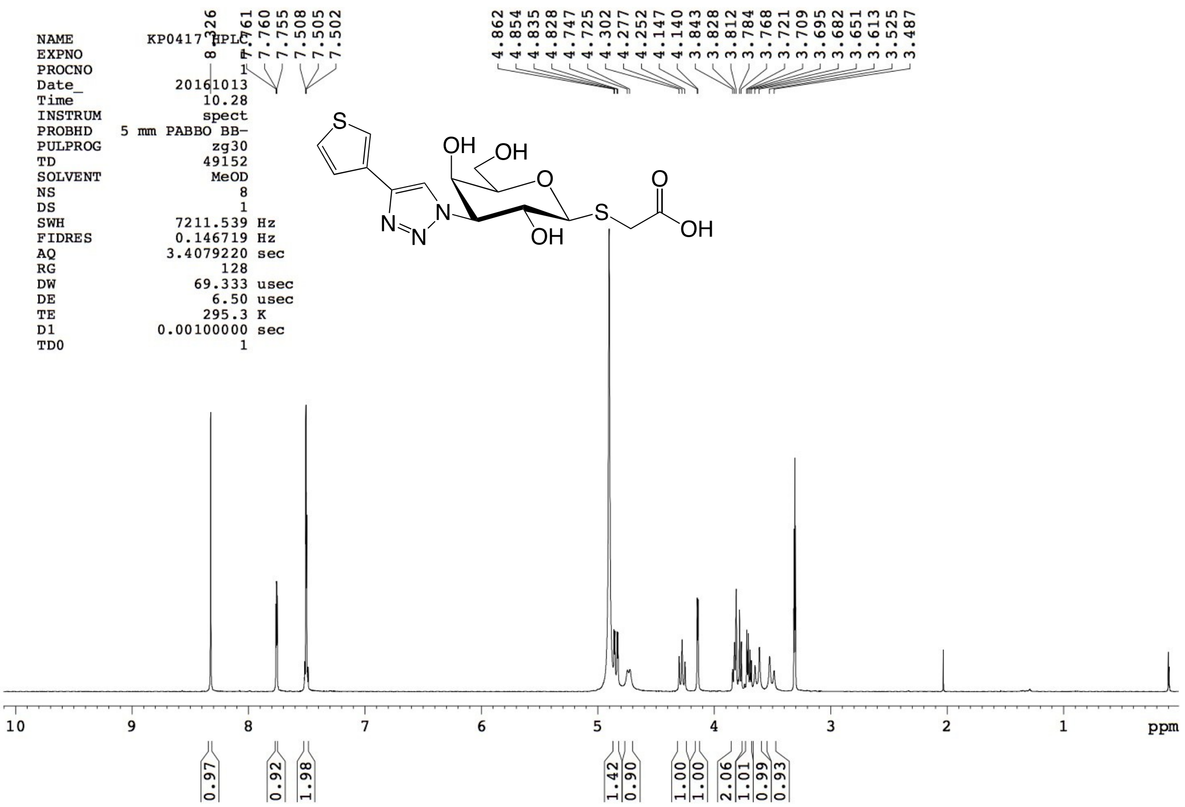

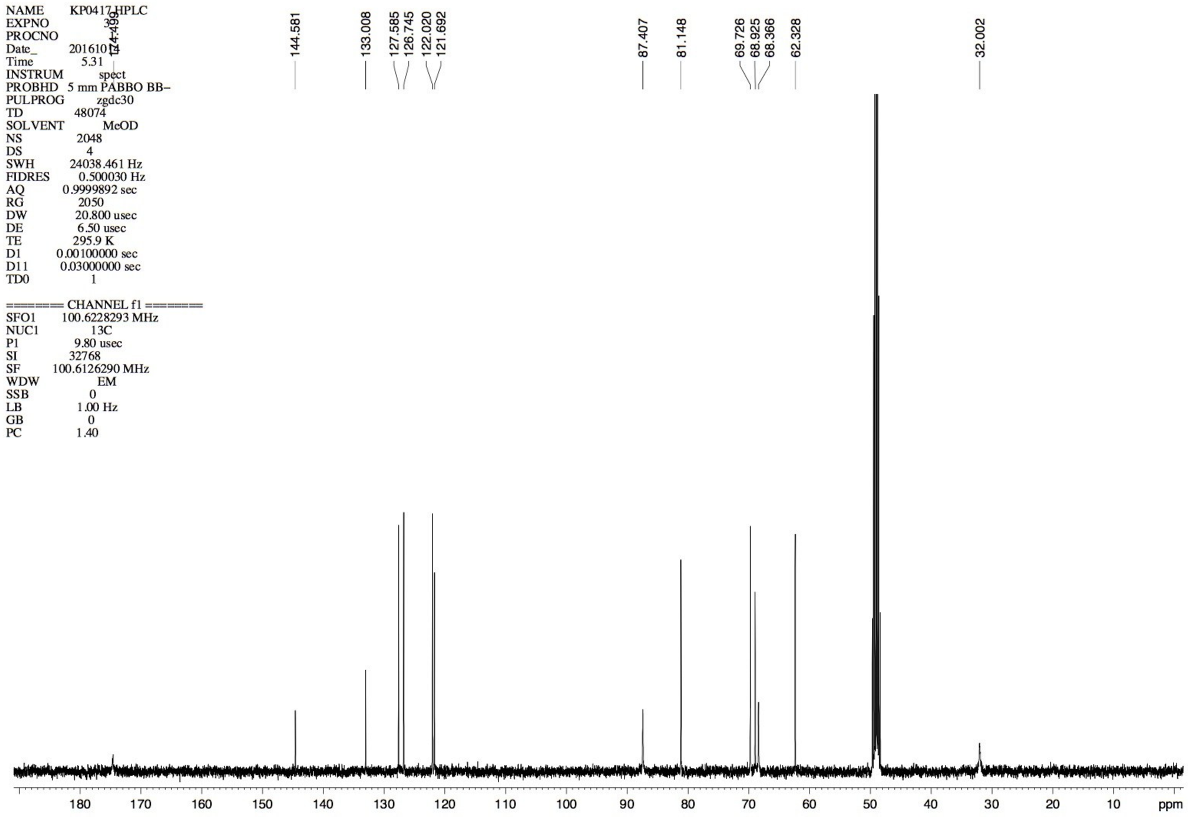

1,3-Dideoxy-3-[4-(thiazol-2-yl)-1*H*-1,2,3-triazol-1-yl]-1-thiocarboxymethyl-β-D-galactopyranoside 13

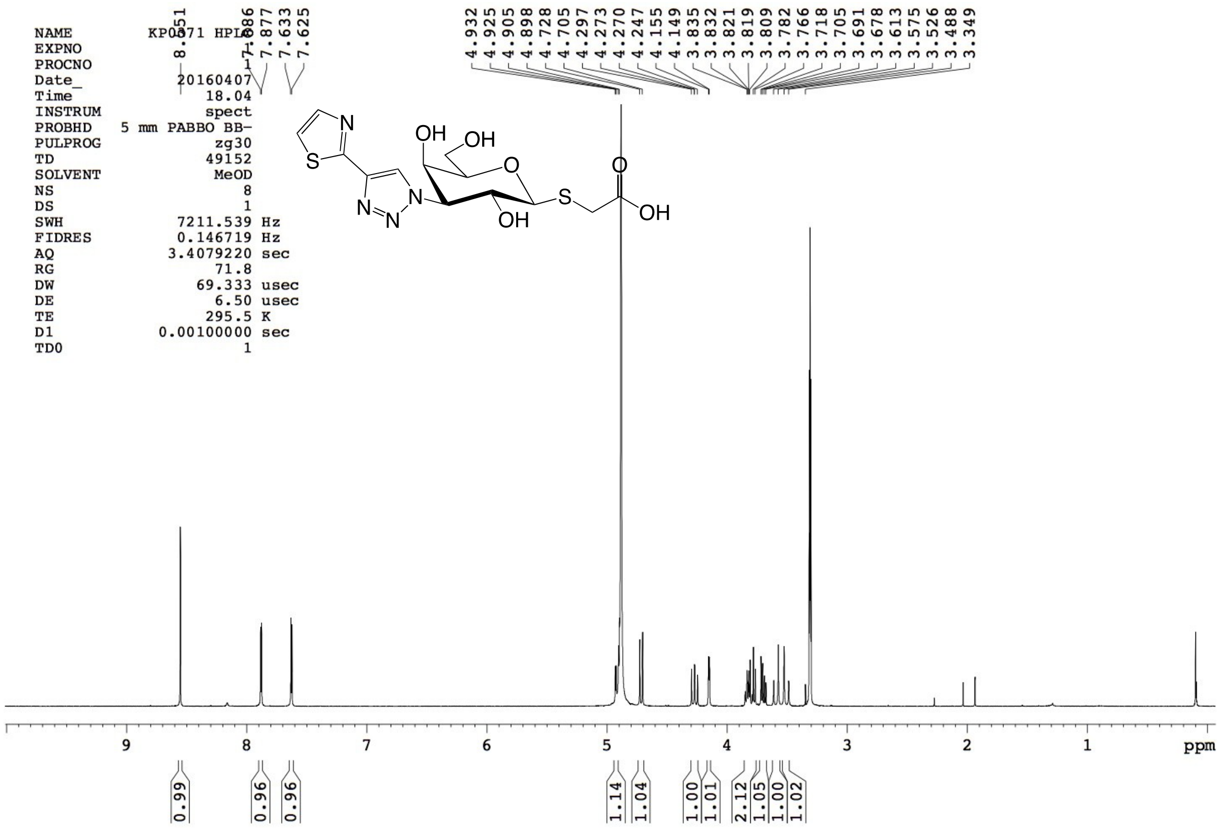

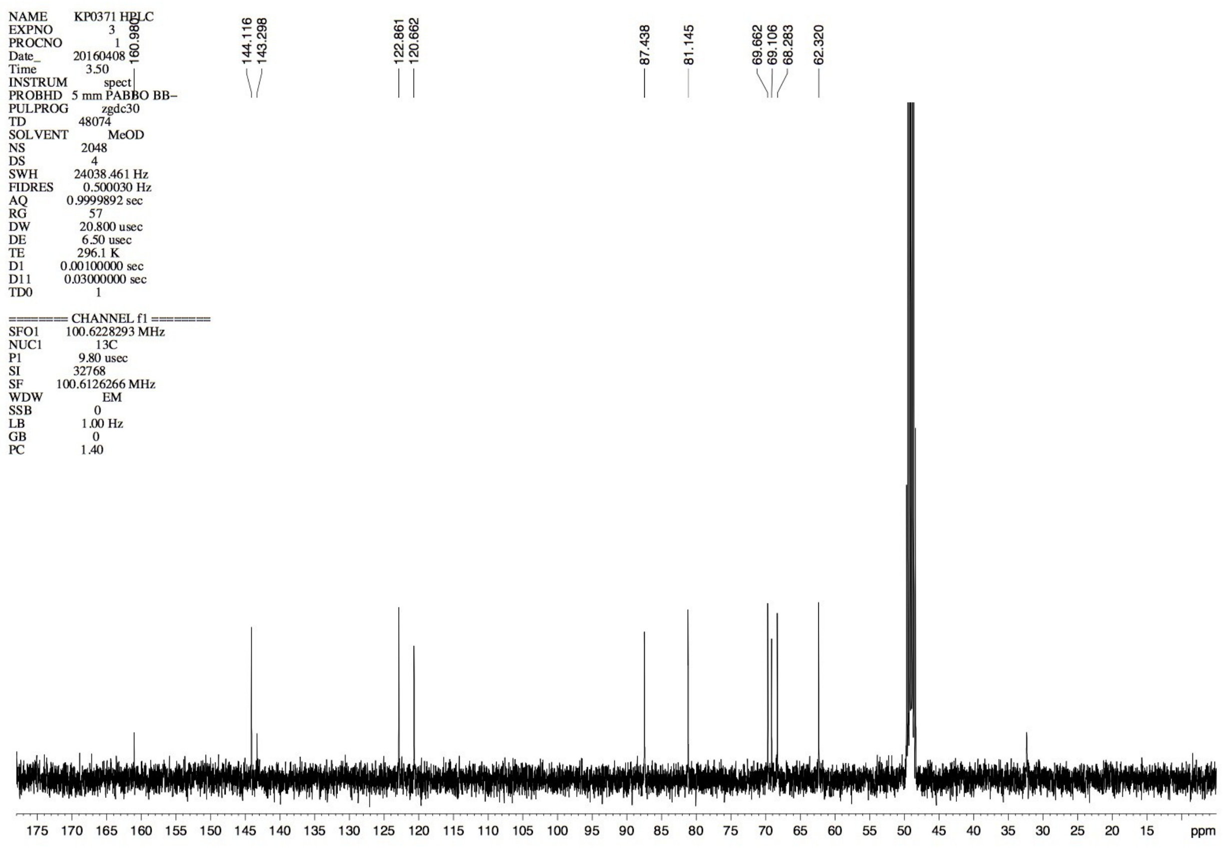

1,3-Dideoxy-3-[4-(thiazol-4-yl)-1*H*-1,2,3-triazol-1-yl]-1-thiocarboxymethyl-β-D-galactopyranoside 14

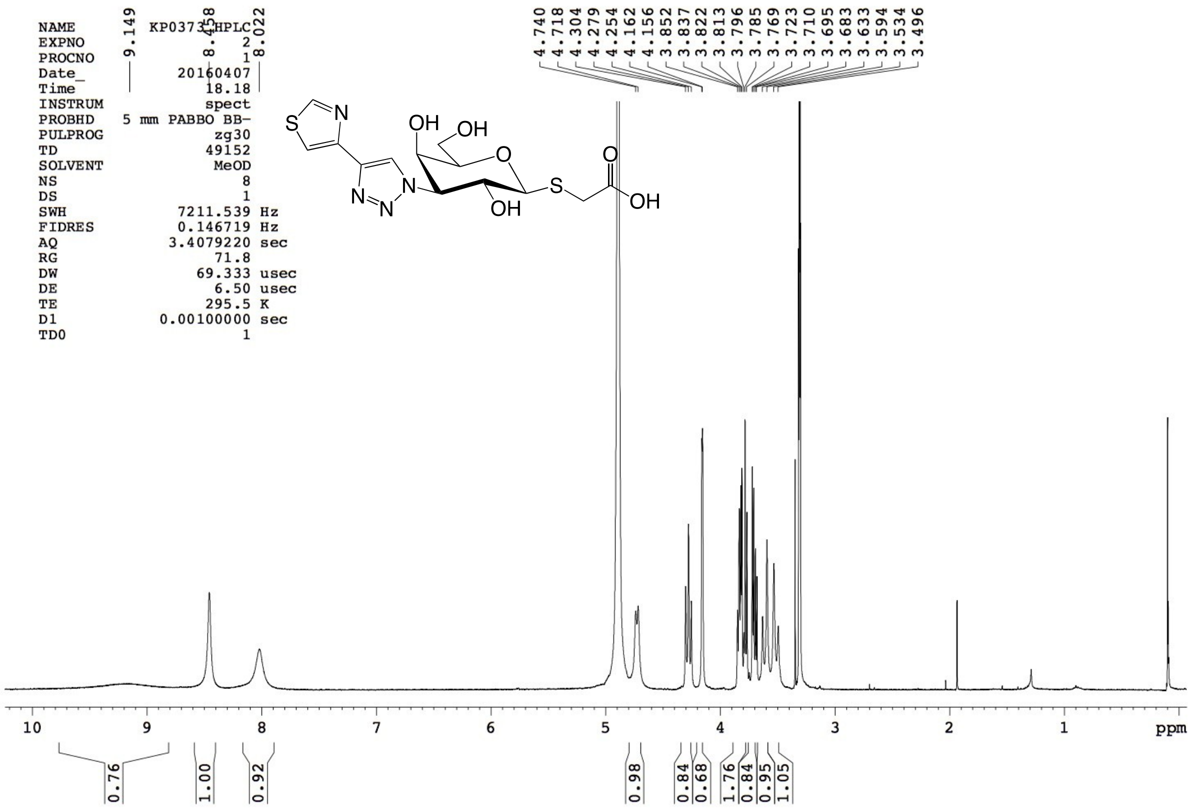

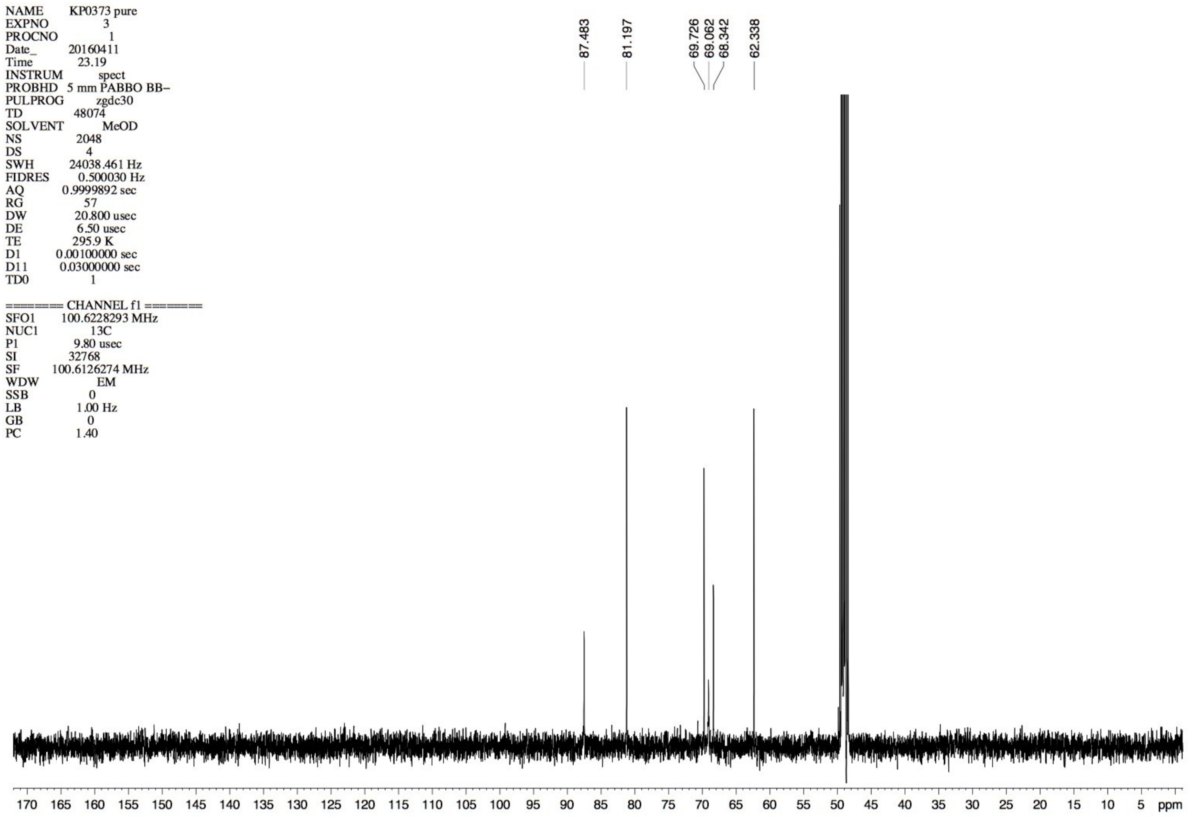

1,3-Dideoxy-3-[4-(thiazol-5-yl)-1*H*-1,2,3-triazol-1-yl]-1-thiocarboxymethyl-β-D-galactopyranoside 15

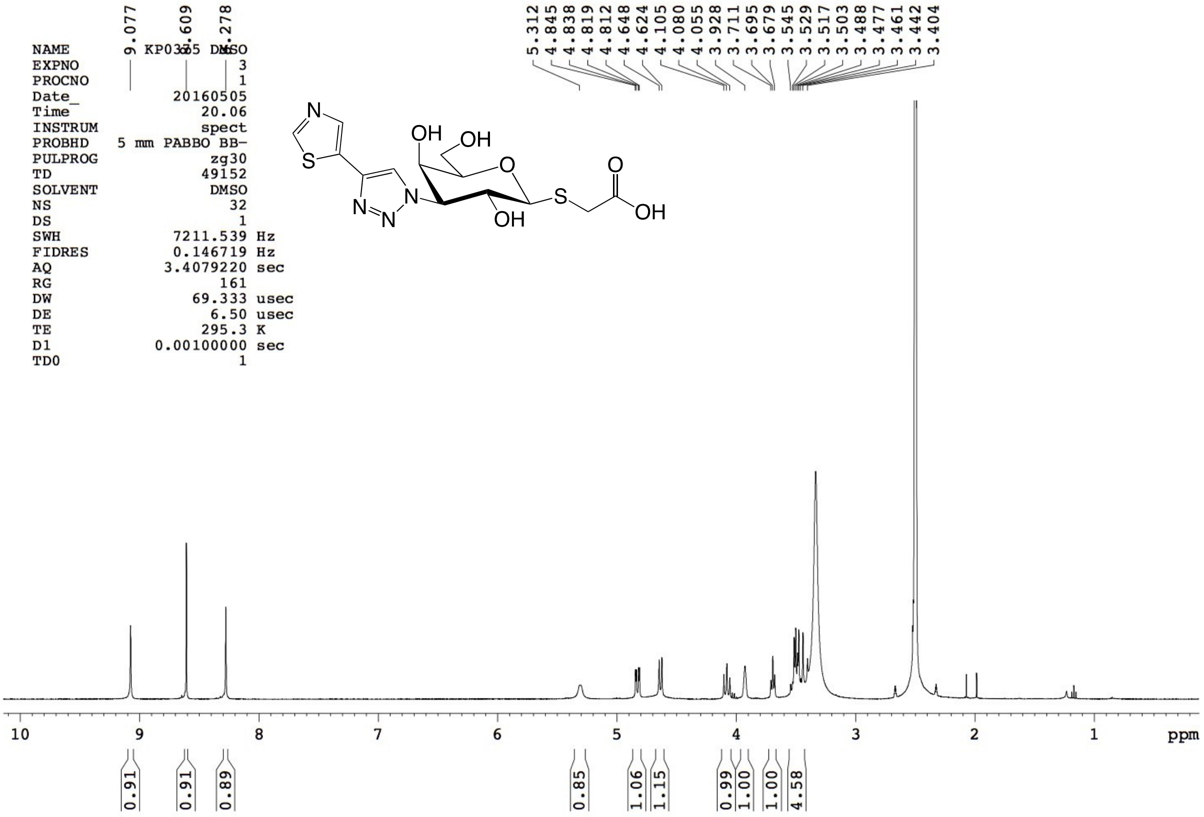

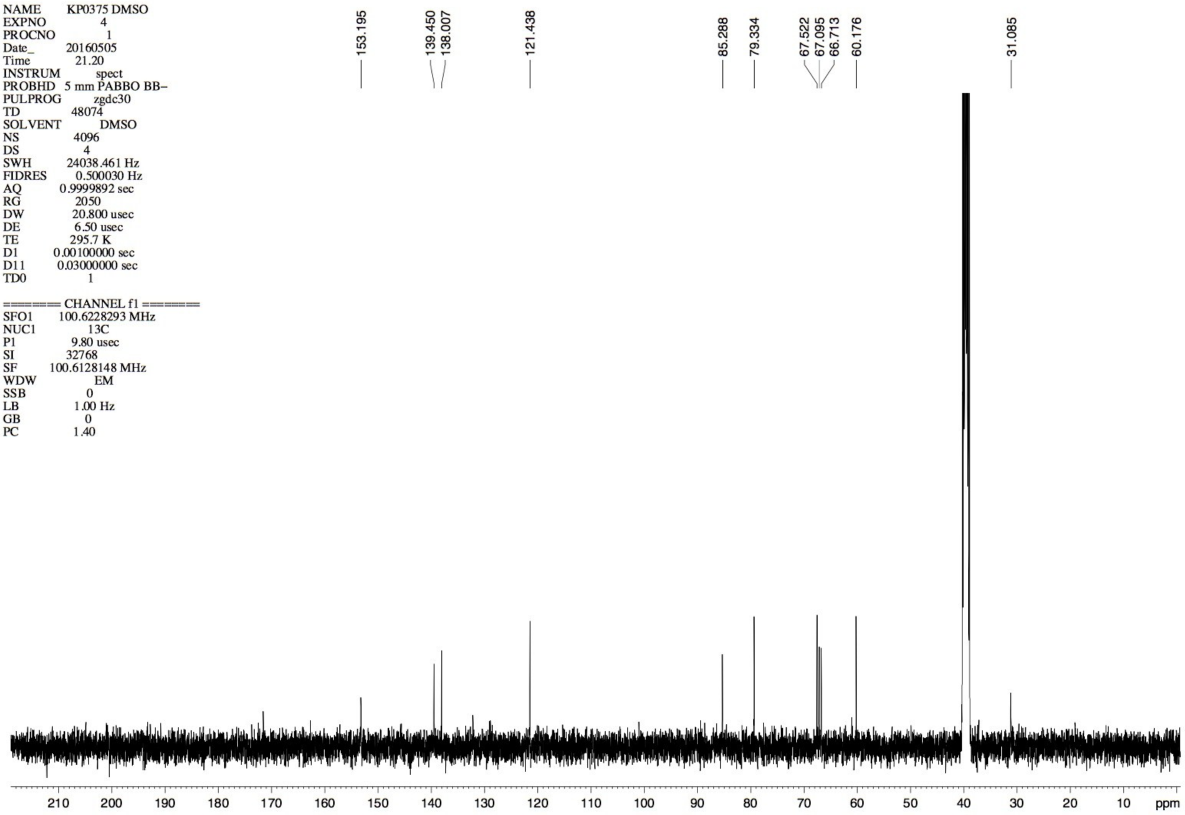

1-1’-sulfanediyl-bis-{3-deoxy-3-[4-(thiazol-2-yl)-1*H*-1,2,3-triazol-1-yl]-β-D-galactopyranoside} 19

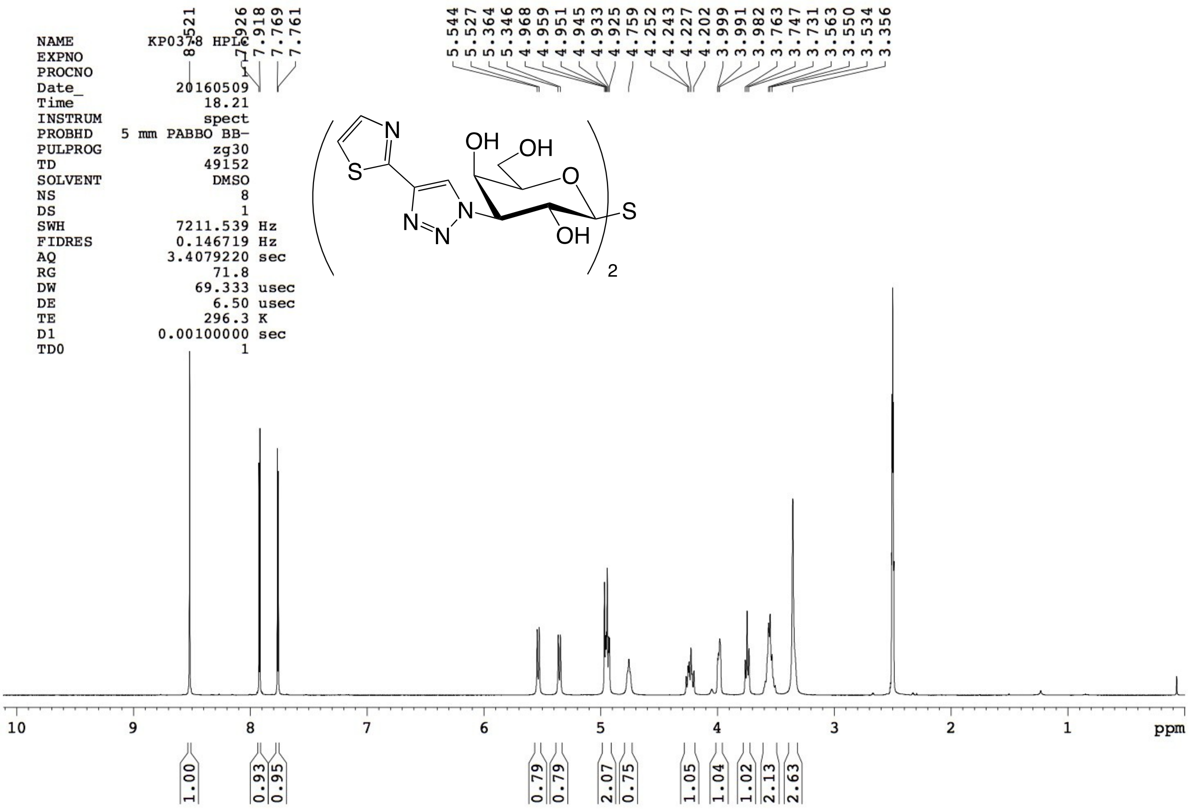

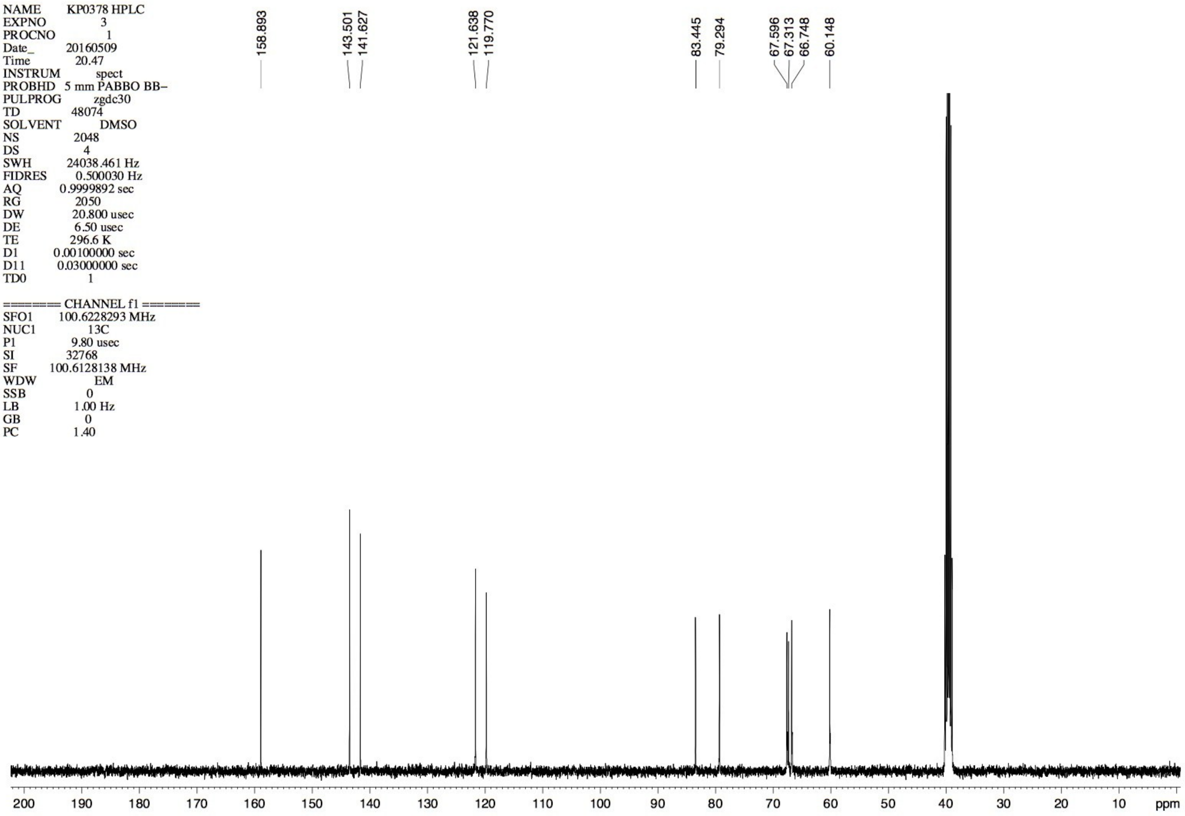

1-1'-sulfanediyl-bis-{3-deoxy-3-[4-(1*H*-imidazol-2-yl)-1*H*-1,2,3-triazol-1-yl]-β-D-galactopyranoside} 20

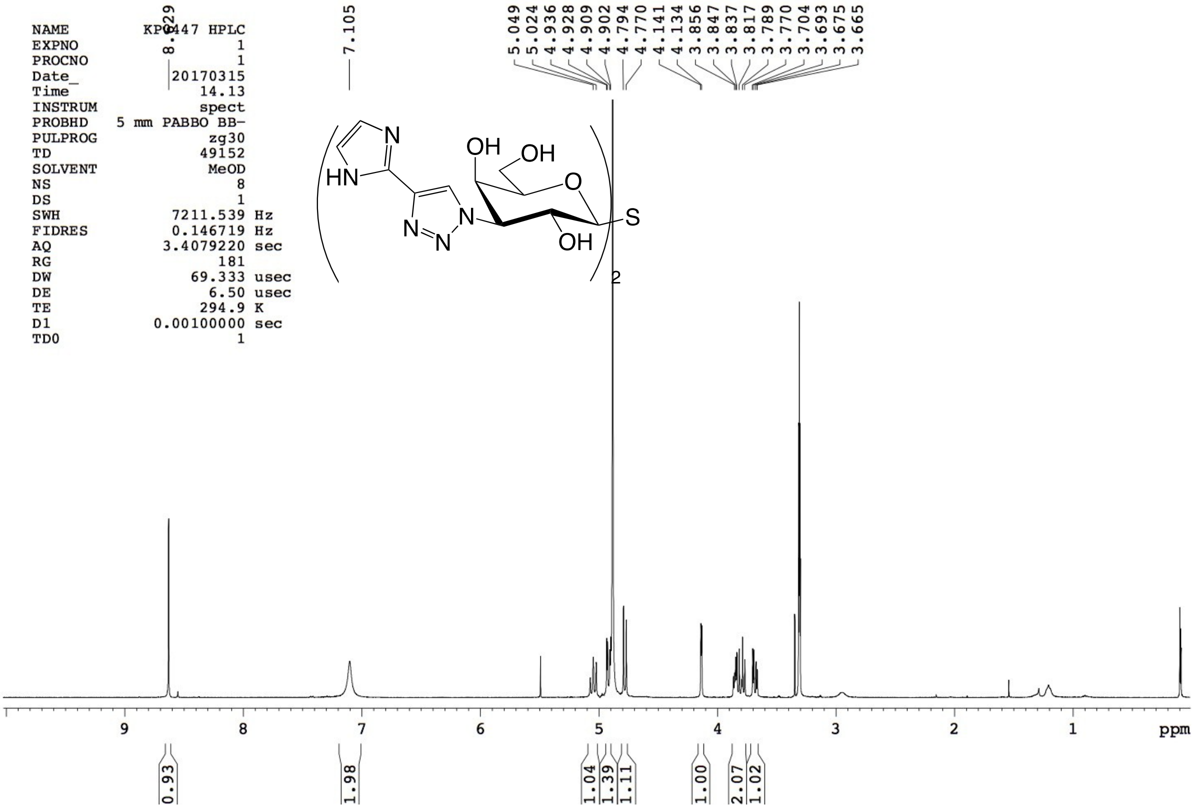

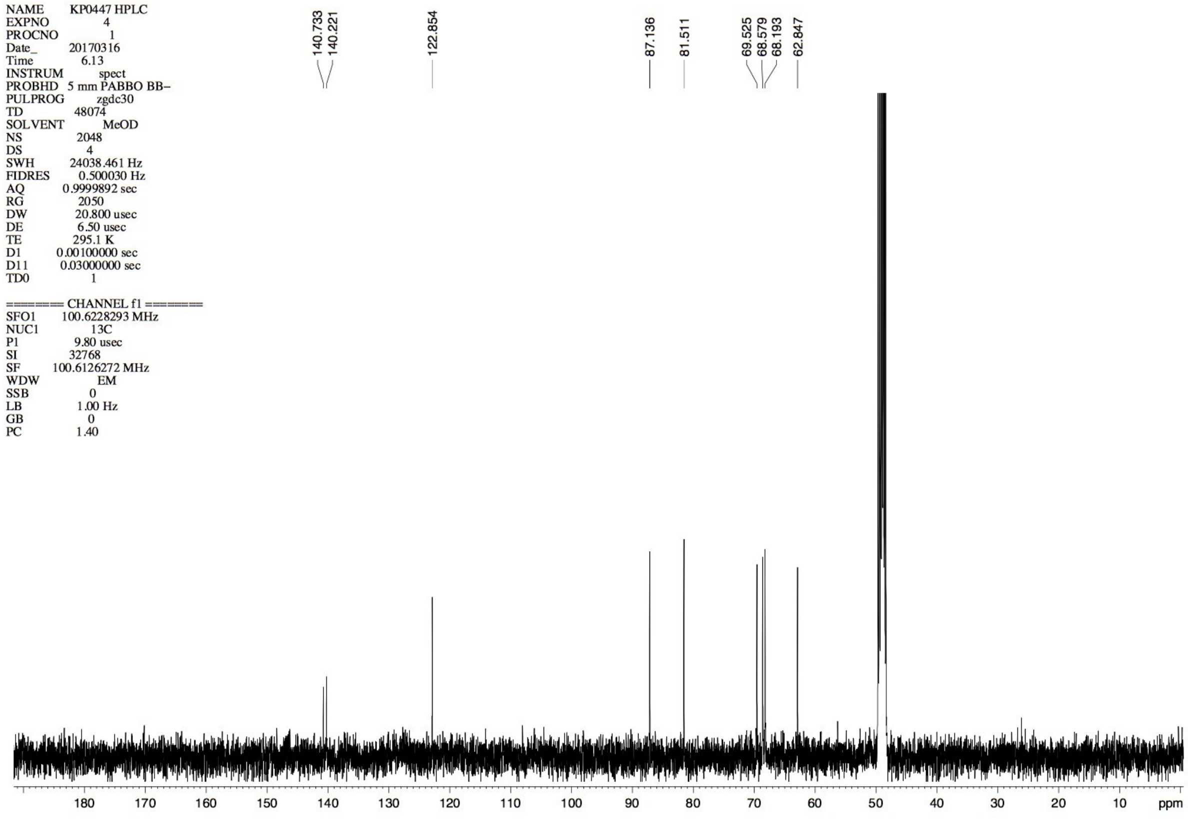

3’-Azido-3-3’-dideoxy-3-[4-(thiazol-2-yl)-1*H*-1,2,3-triazol-1-yl]-1,1’-sulfanediyl-di-β-D-galactopyranoside 21

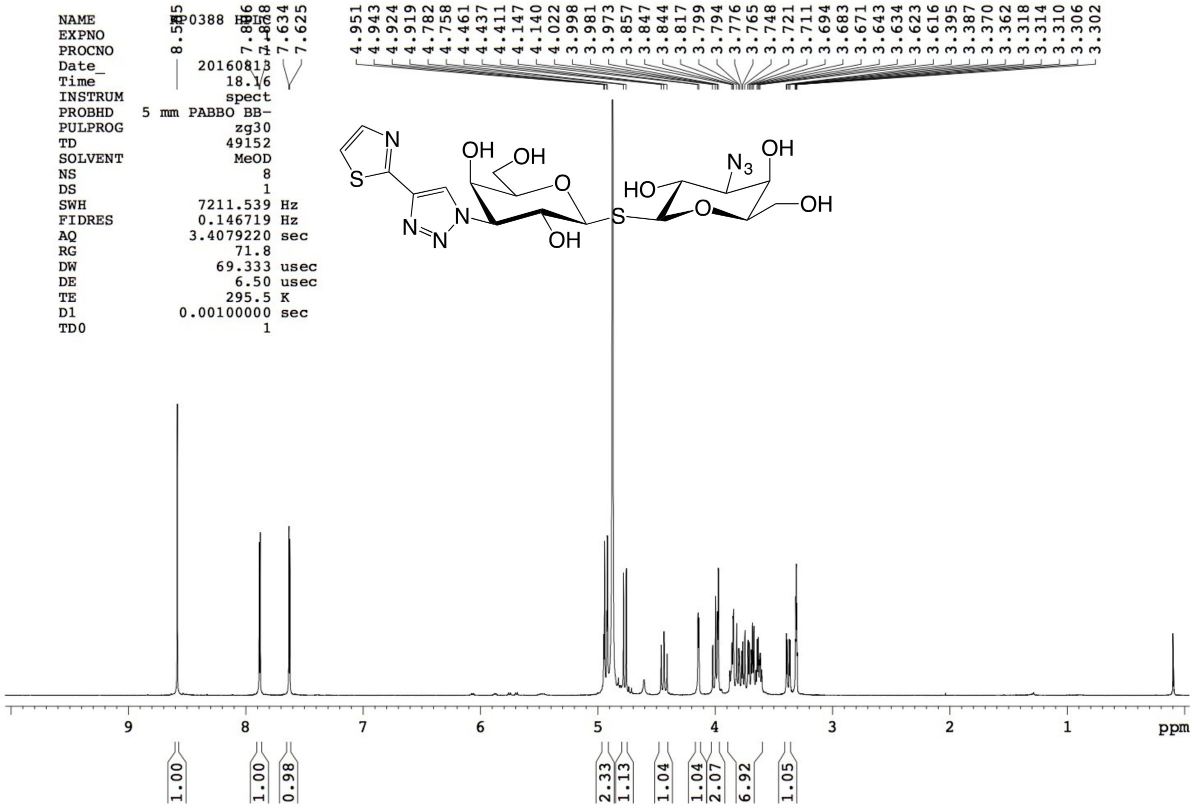

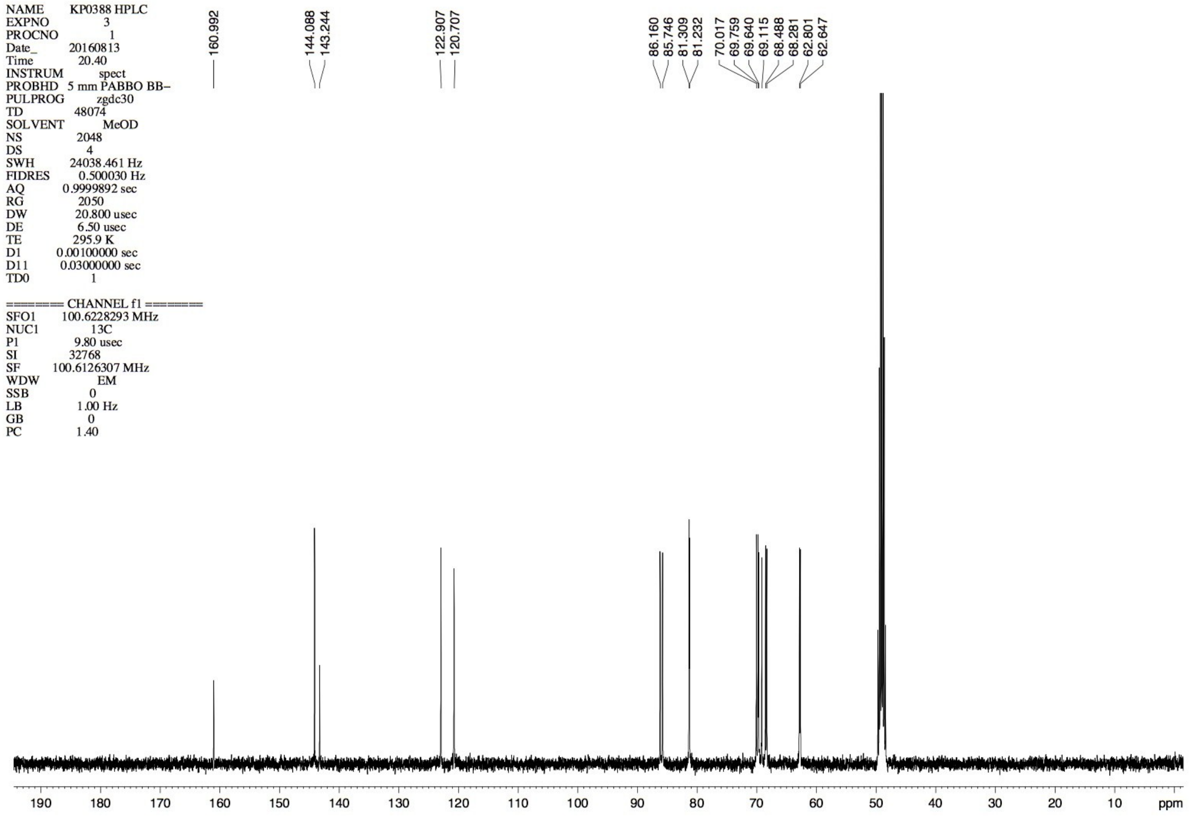

3,3’-Dideoxy-3-[4-(thiazol-2-yl)-1*H*-1,2,3-triazol-1-yl]-3’-[4-(thien-3-yl)-1*H*-1,2,3-triazol-1-yl]-1,1’-sulfanediyl-di-β-D-galactopyranoside 22

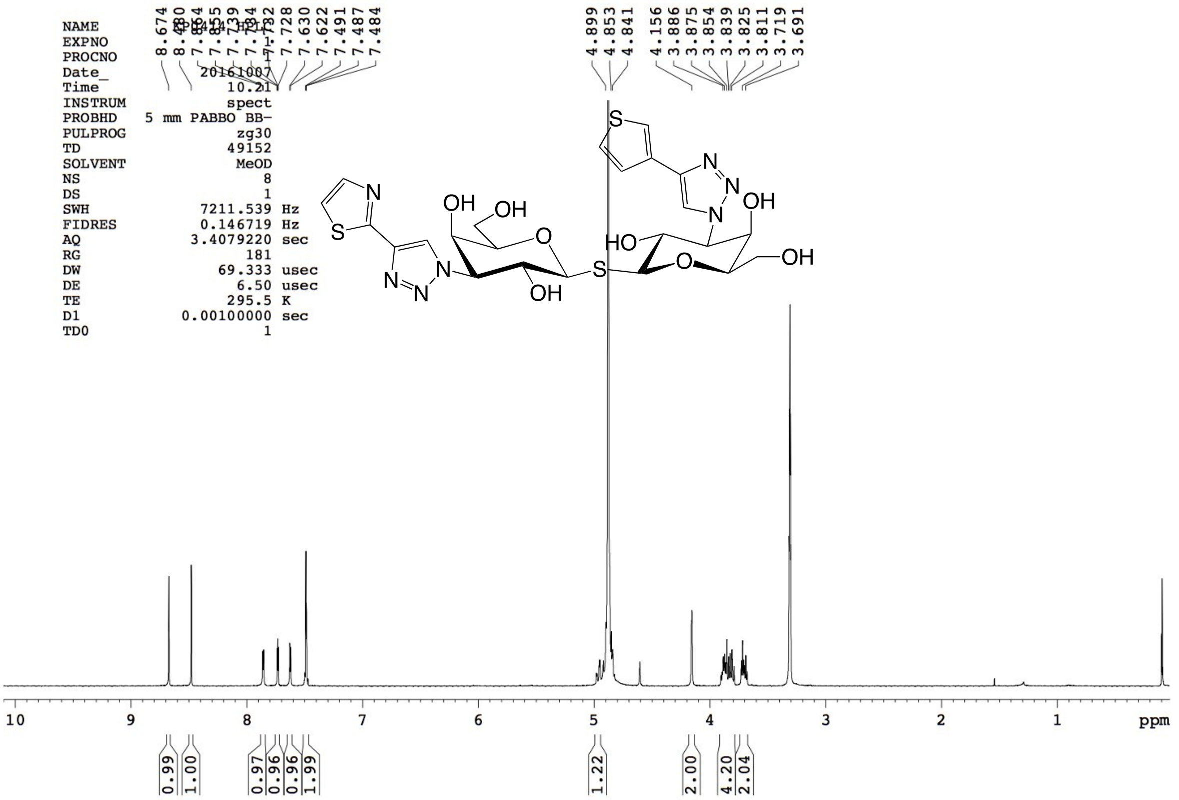

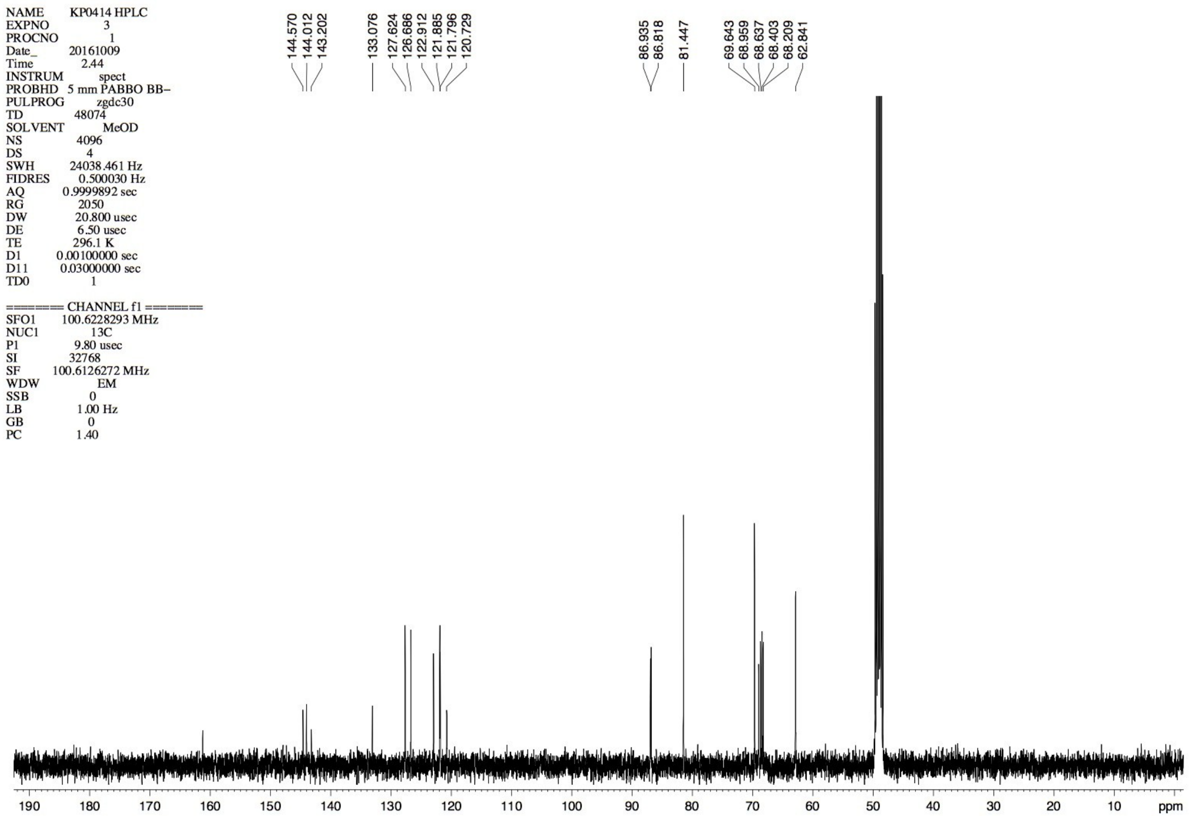

3,3’-Dideoxy-3-[4-(thiazol-2-yl)-1*H*-1,2,3-triazol-1-yl]-3’-[4-(3,4-difluorophenyl)-1*H*-1,2,3-triazol-1-yl]-1,1’-sulfanediyl-di-β-D-galactopyranoside 23

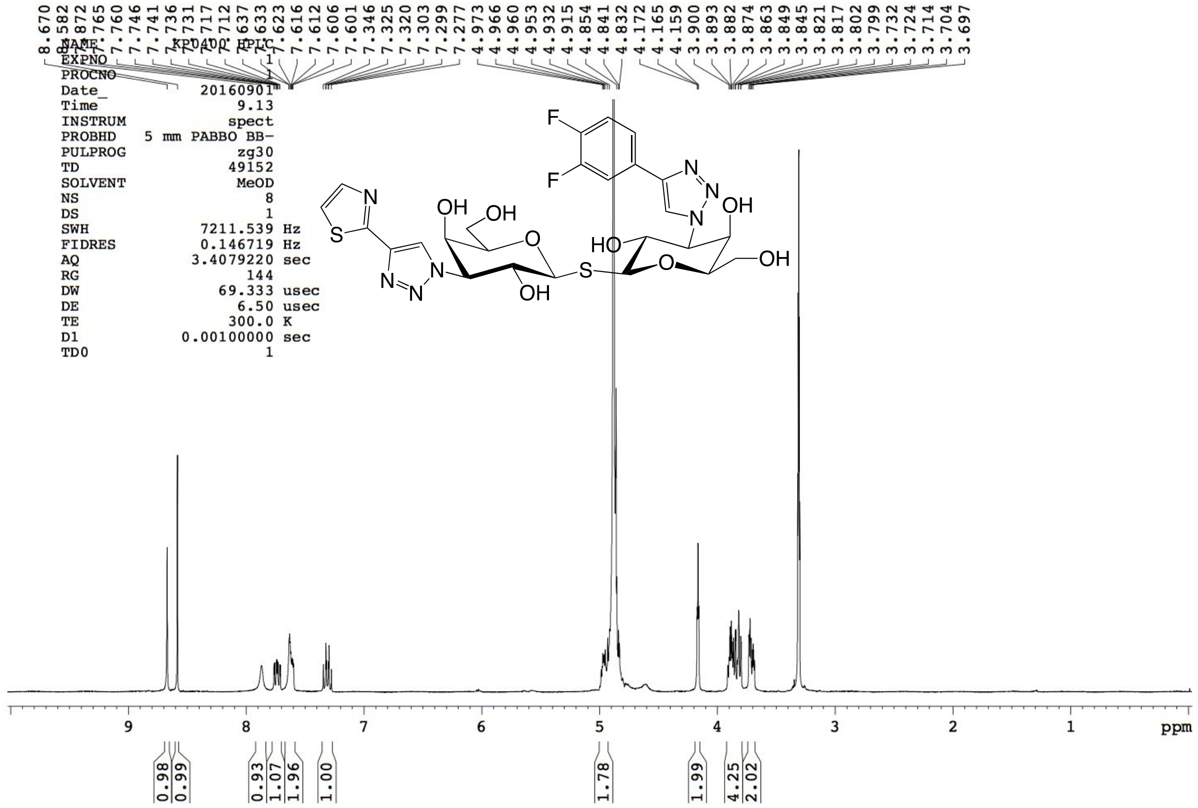

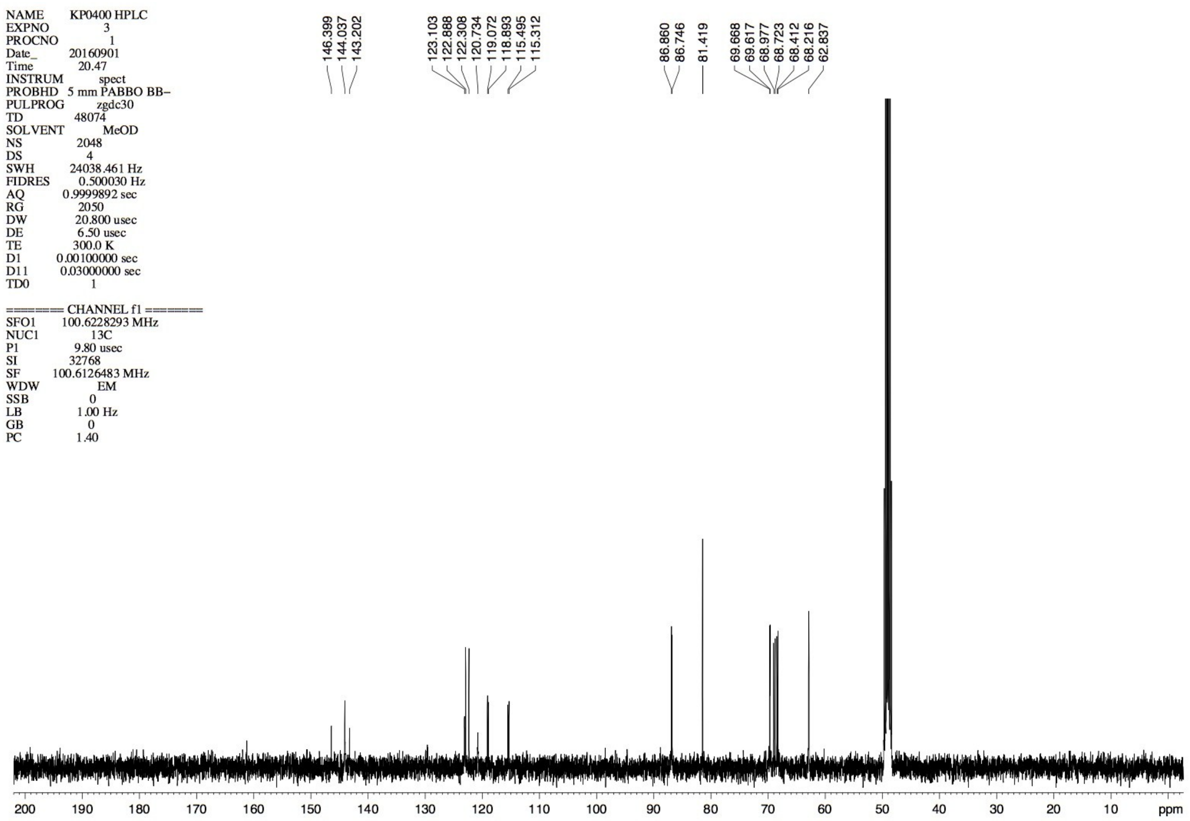

3’-Amino-3-3’-dideoxy-3-[4-(thiazol-2-yl)-1*H*-1,2,3-triazol-1-yl]-1,1’-sulfanediyl-di-β-D-galactopyranoside 24

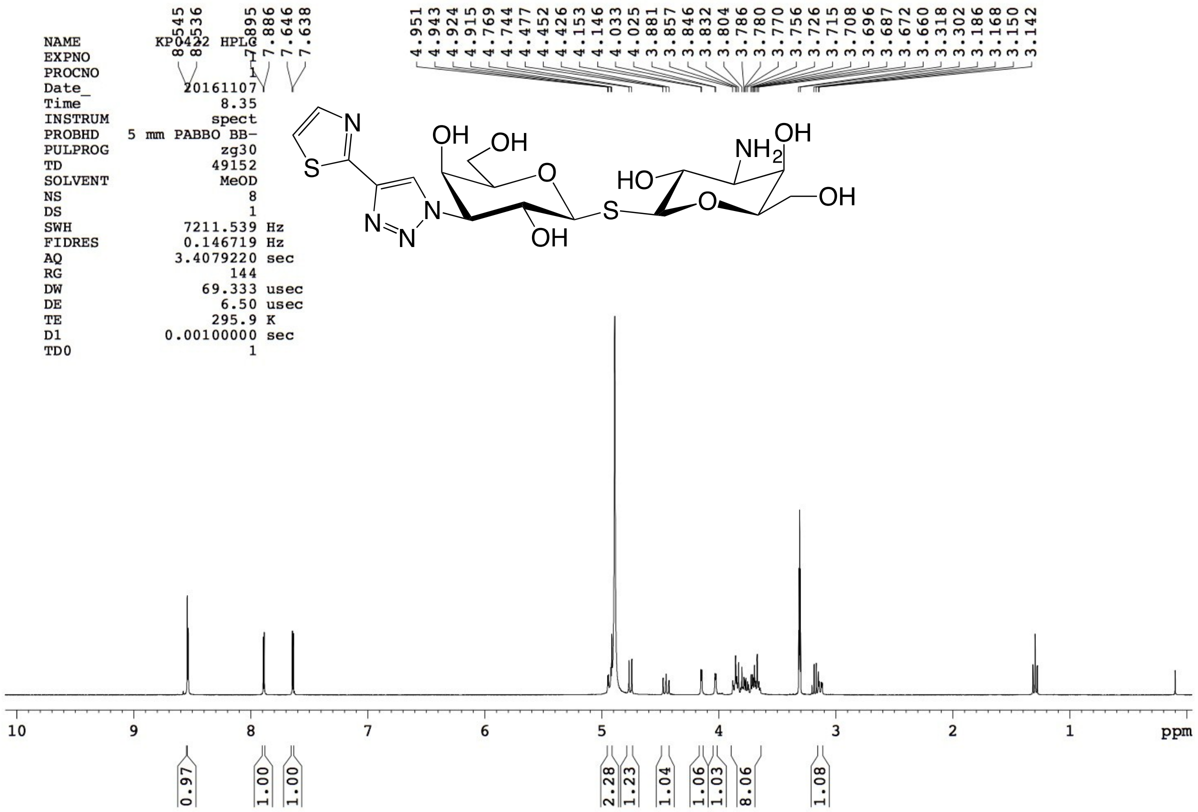

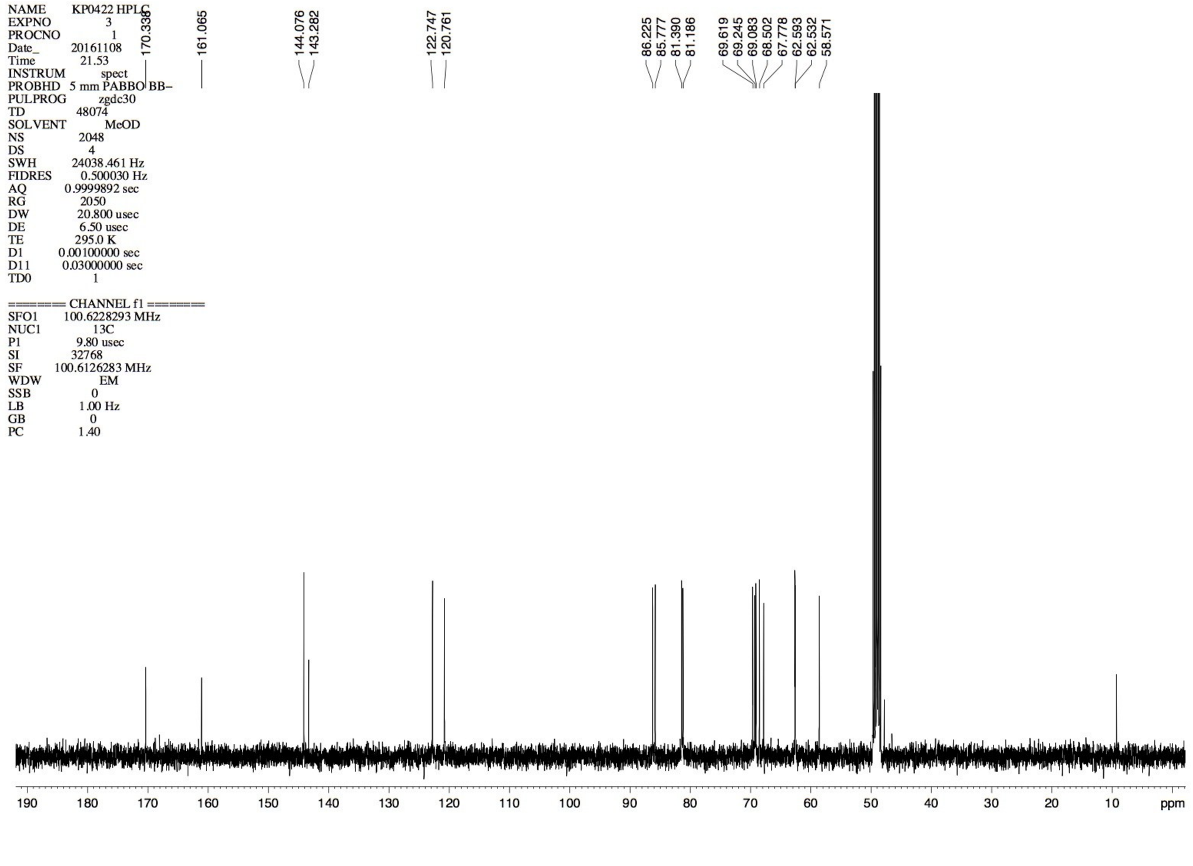

3,3'-Dideoxy-3-(fluorescein-5-yl-carbonylamino)-3'-[4-(thiazol-2-yl)-1*H*-1,2,3-triazol-1-yl]-1,1'-sulfanediyl-di-β-D-galactopyranoside 25

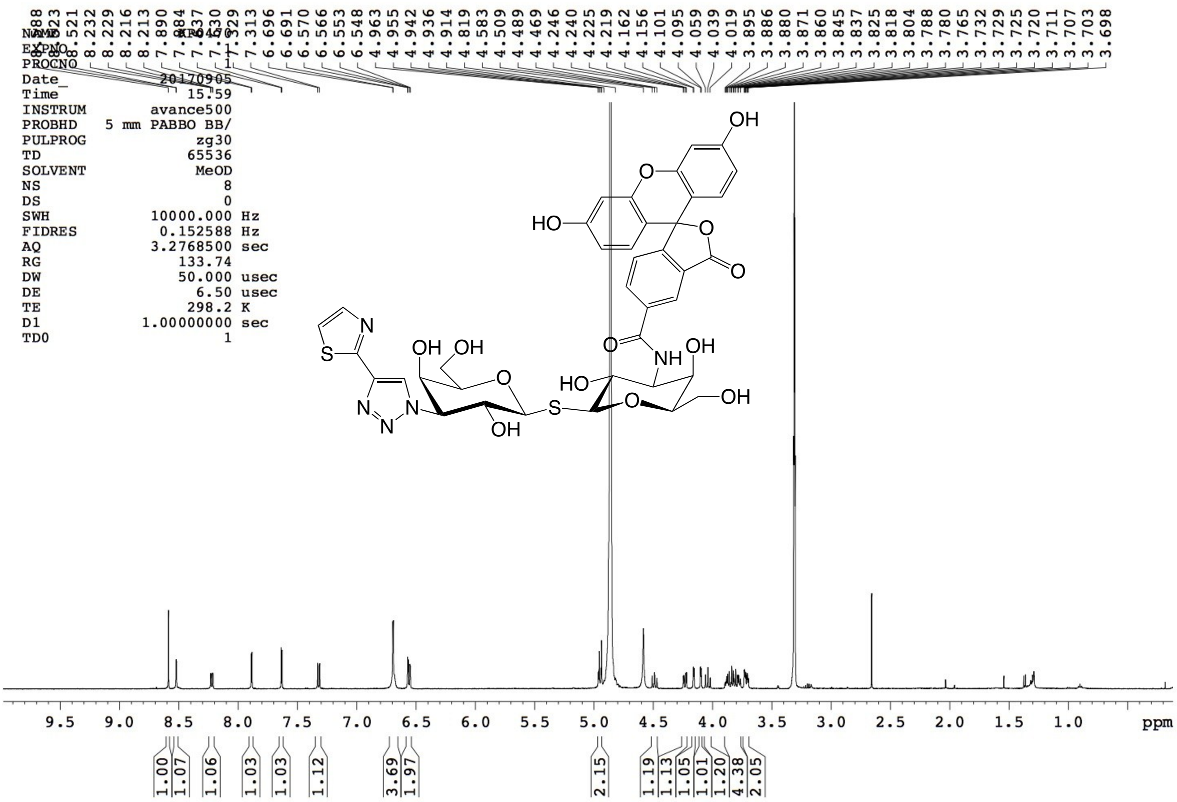

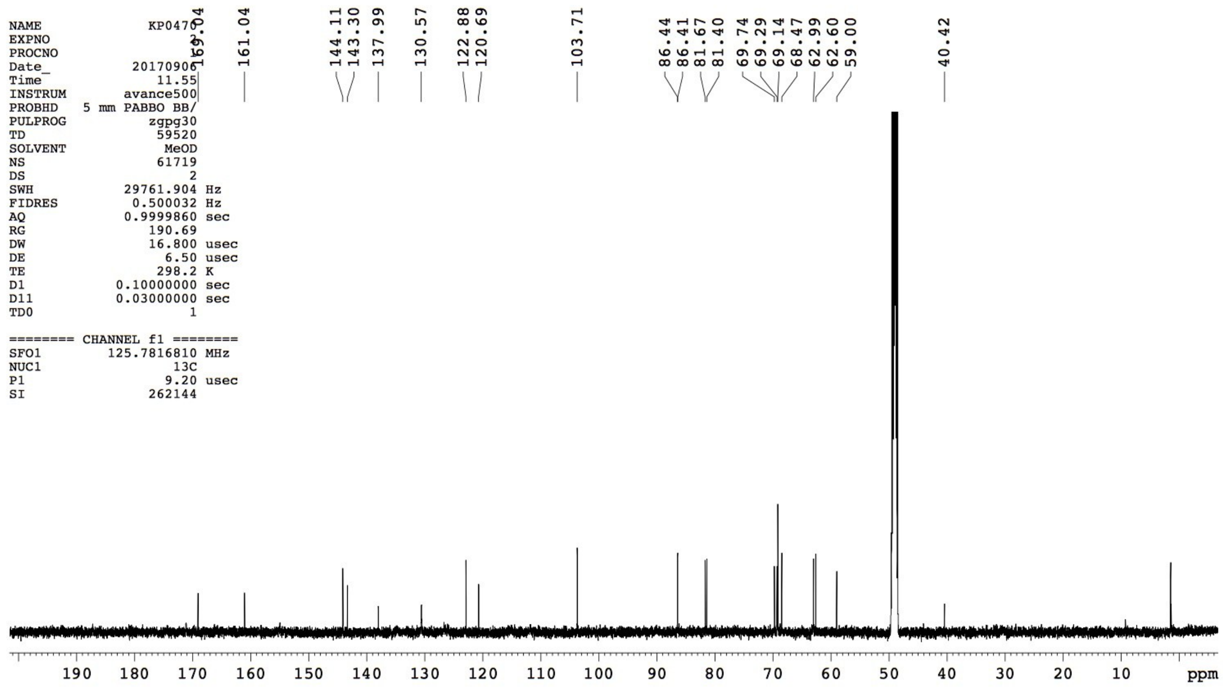

Purity chromatograms

***p*-Methylphenyl 3-deoxy-3-[4-(cyclopentyl)-1*H*-1,2,3-triazol-1-yl]-1-thio-β-D-galactopyranoside 4**

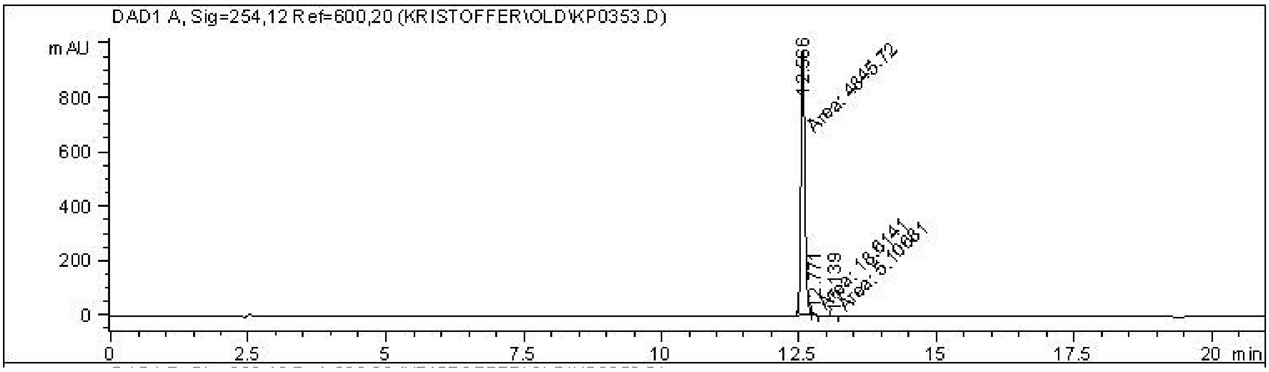

***p*-Methylphenyl 3-deoxy-3-[4-(furan-3-yl)-1*H*-1,2,3-triazol-1-yl]-1-thio-β-D-galactopyranoside 5**

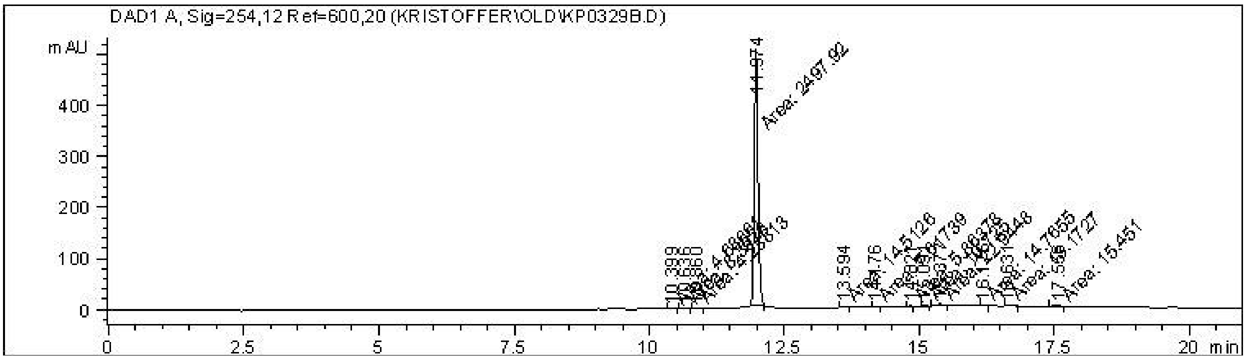

***p*-Methylphenyl 3-deoxy-3-[4-(thien-2-yl)-1*H*-1,2,3-triazol-1-yl]-1-thio-β-D-galactopyranoside 6**

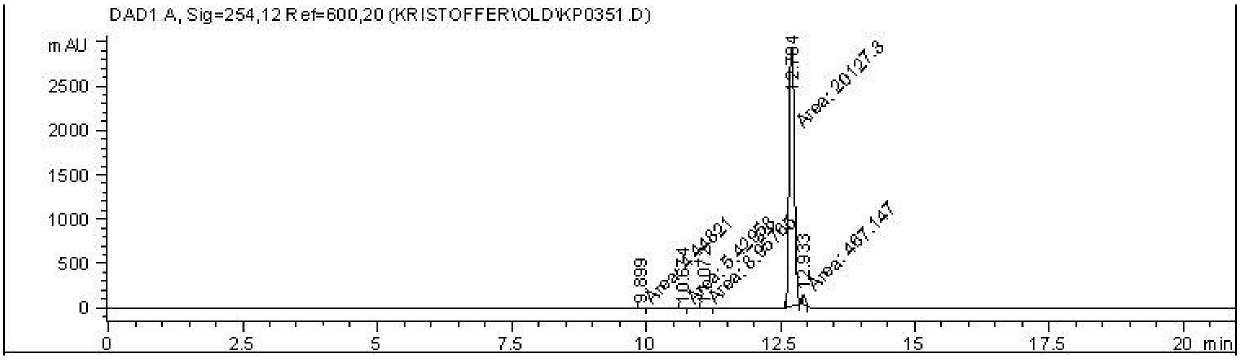

***p*-Methylphenyl 3-deoxy-3-[4-(thien-3-yl)-1*H*-1,2,3-triazol-1-yl]-1-thio-β-D-galactopyranoside 7**

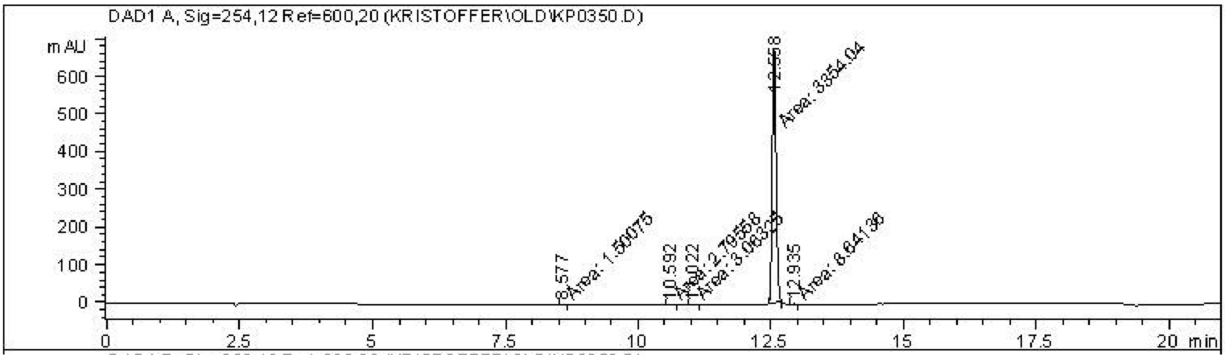

***p*-Methylphenyl 3-deoxy-3-[4-(1*H*-imidazol-2-yl)-1*H*-1,2,3-triazol-1-yl]-1-thio-β-D-galactopyranoside 8**

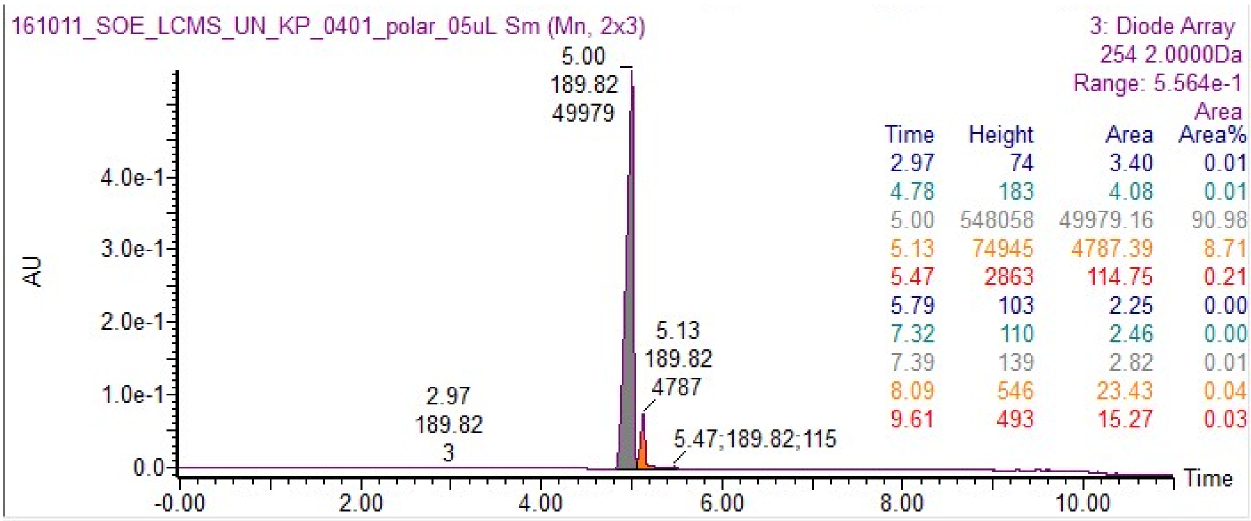

Comment: The two main peaks are isomers with exact mass corresponding to compound **8** and when the analyzed at higher temperature the two peaks merge into one, indicating an equilibrium of isomers.

_p_-Methylphenyl 3-deoxy-3-[4-(1_H_-imidazol-4-yl)-1_H_-1,2,3-triazol-1-yl]-1-thio-β-D-galactopyranoside 9

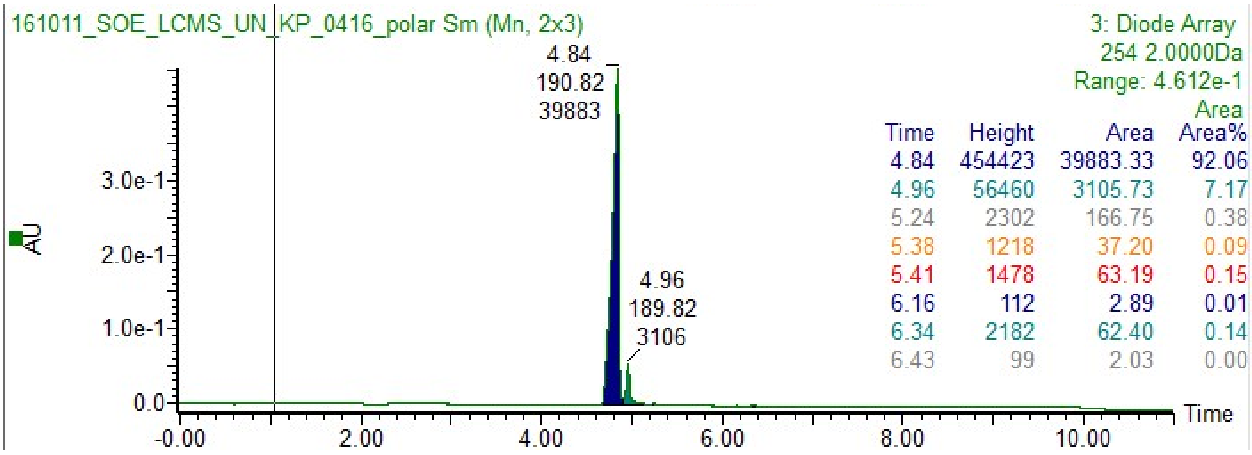

Comment: The two main peaks are isomers with exact mass corresponding to compound **8** and when the analyzed at higher temperature the two peaks merge into one, indicating an equilibrium of isomers.

1,3-Dideoxy-3-[4-(thien-3-yl)-1_H_-1,2,3-triazol-1-yl]-1-thiocarboxymethyl-β-D-galactopyranoside 12

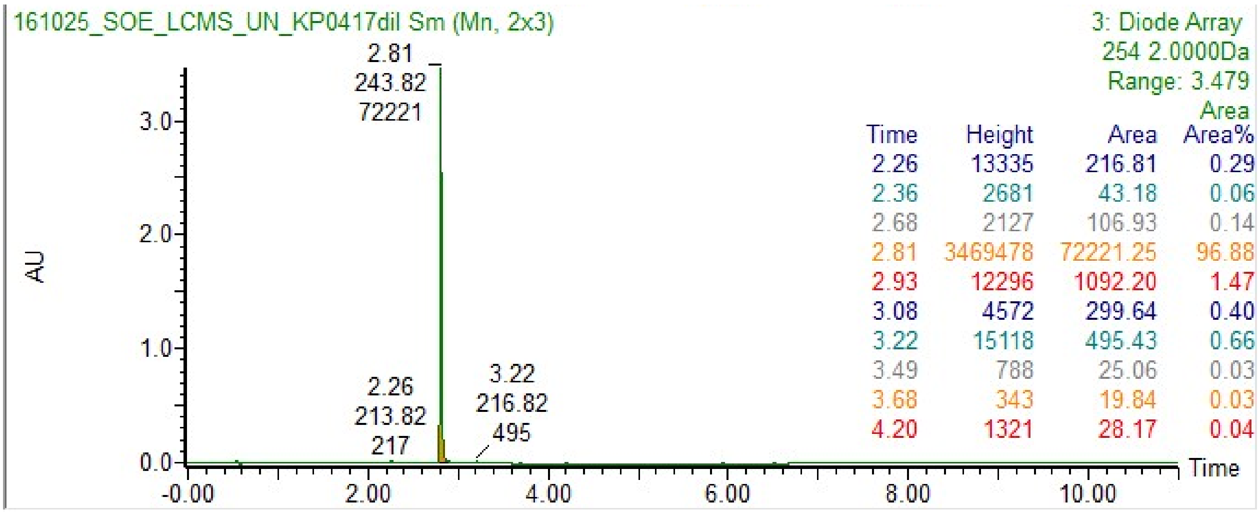

1,3-Dideoxy-3-[4-(thiazol-2-yl)-1*H*-1,2,3-triazol-1-yl]-1-thiocarboxymethyl-β-D-galactopyranoside 13

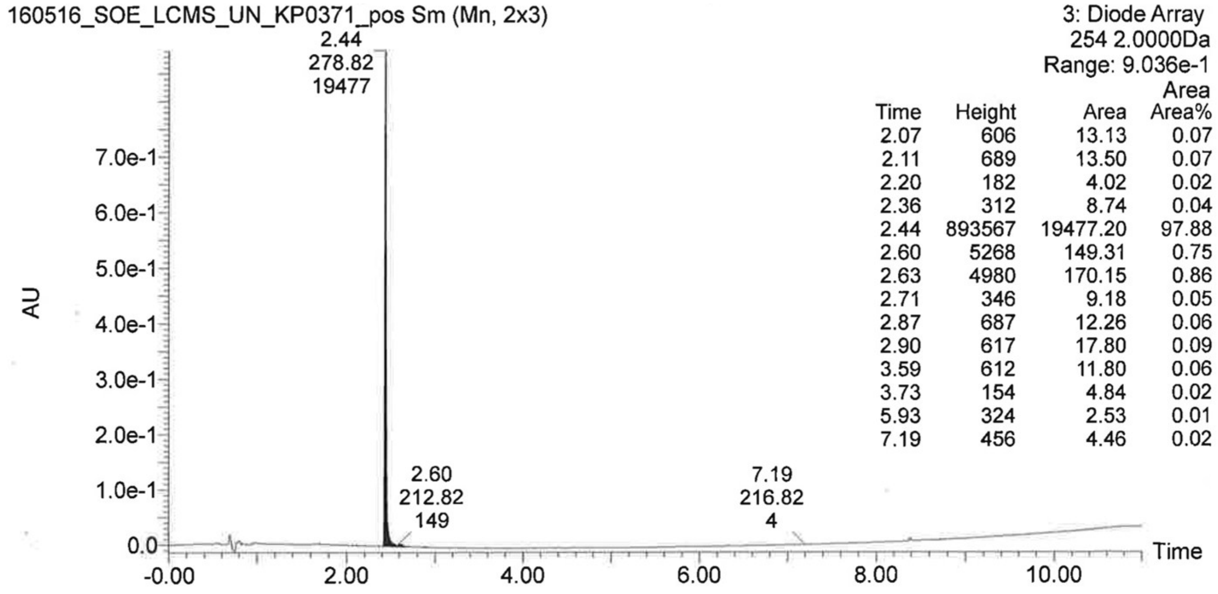

1,3-Dideoxy-3-[4-(thiazol-4-yl)-1*H*-1,2,3-triazol-1-yl]-1-thiocarboxymethyl-β-D-galactopyranoside 14

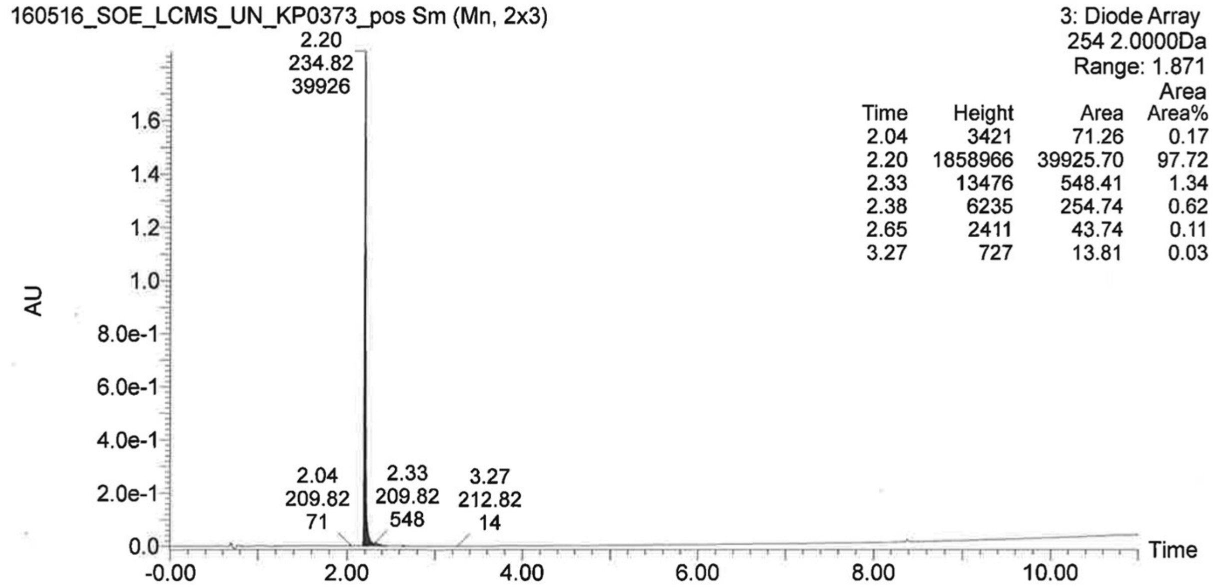

1,3-Dideoxy-3-[4-(thiazol-5-yl)-1*H*-1,2,3-triazol-1-yl]-1-thiocarboxymethyl-β-D-galactopyranoside 15

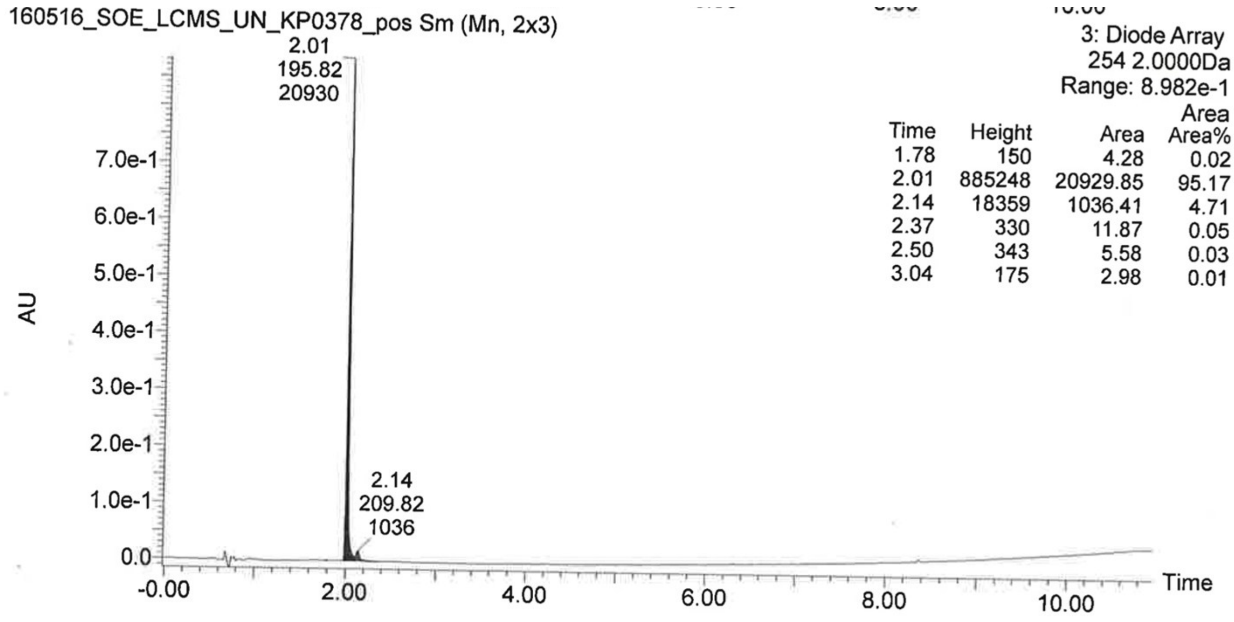

1-1'-sulfanediyl-bis-{3-deoxy-3-[4-(thiazol-2-yl)-1*H*-1,2,3-triazol-1-yl]-β-D-galactopyranoside} 19

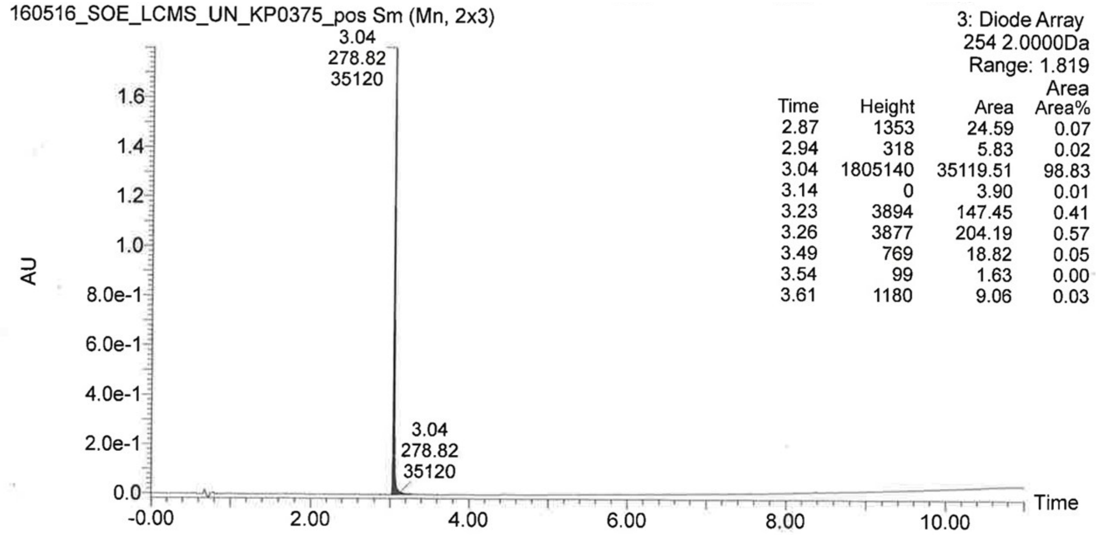

1-1'-sulfanediyl-bis-{3-deoxy-3-[4-(1*H*-imidazol-2-yl)-1*H*-1,2,3-triazol-1-yl]-β-D-galactopyranoside} 20

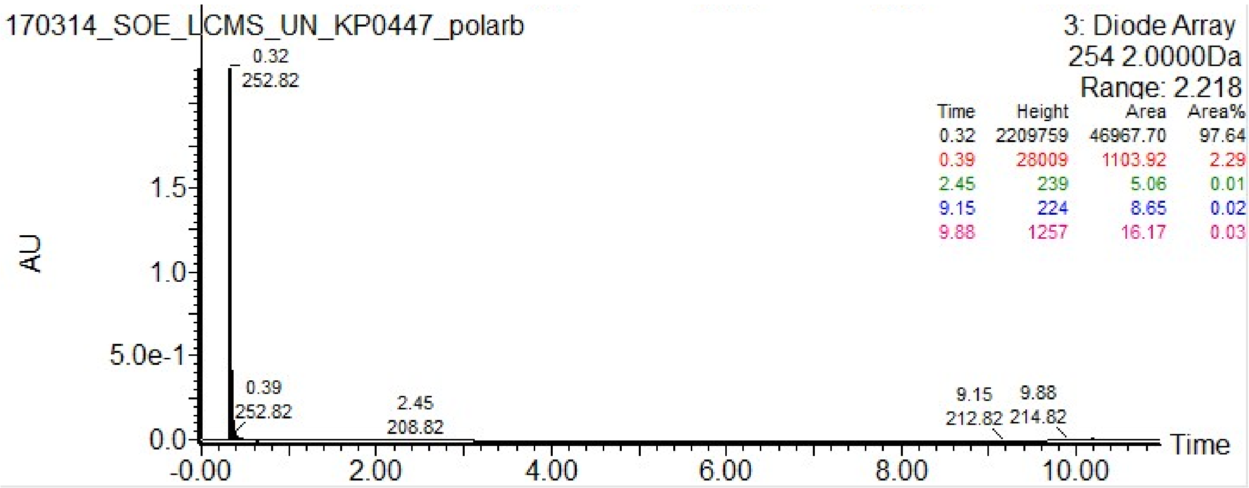

3,3'-Dideoxy-3-[4-(thiazol-2-yl)-1*H*-1,2,3-triazol-1-yl]-3'-[4-(thien-3-yl)-1*H*-1,2,3-triazol-1-yl]-1,1'-sulfanediyl-di-β-D-galactopyranoside 22

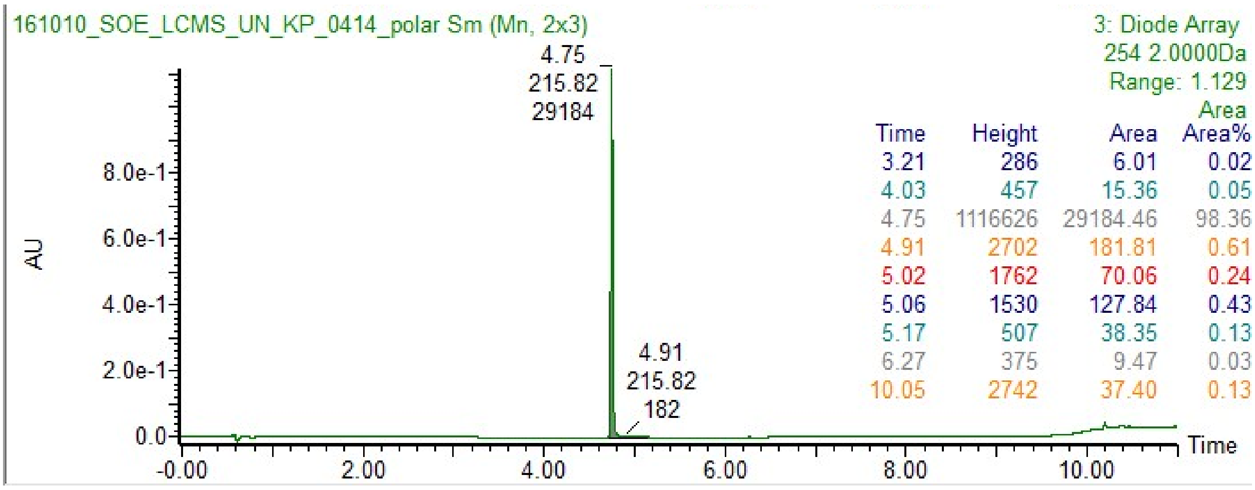

3,3’-Dideoxy-3-[4-(thiazol-2-yl)-1*H*-1,2,3-triazol-1-yl]-3’-[4-(3,4-difluorophenyl)-1*H*-1,2,3-triazol-1-yl]-1,1’-sulfanediyl-di-β-D-galactopyranoside 23

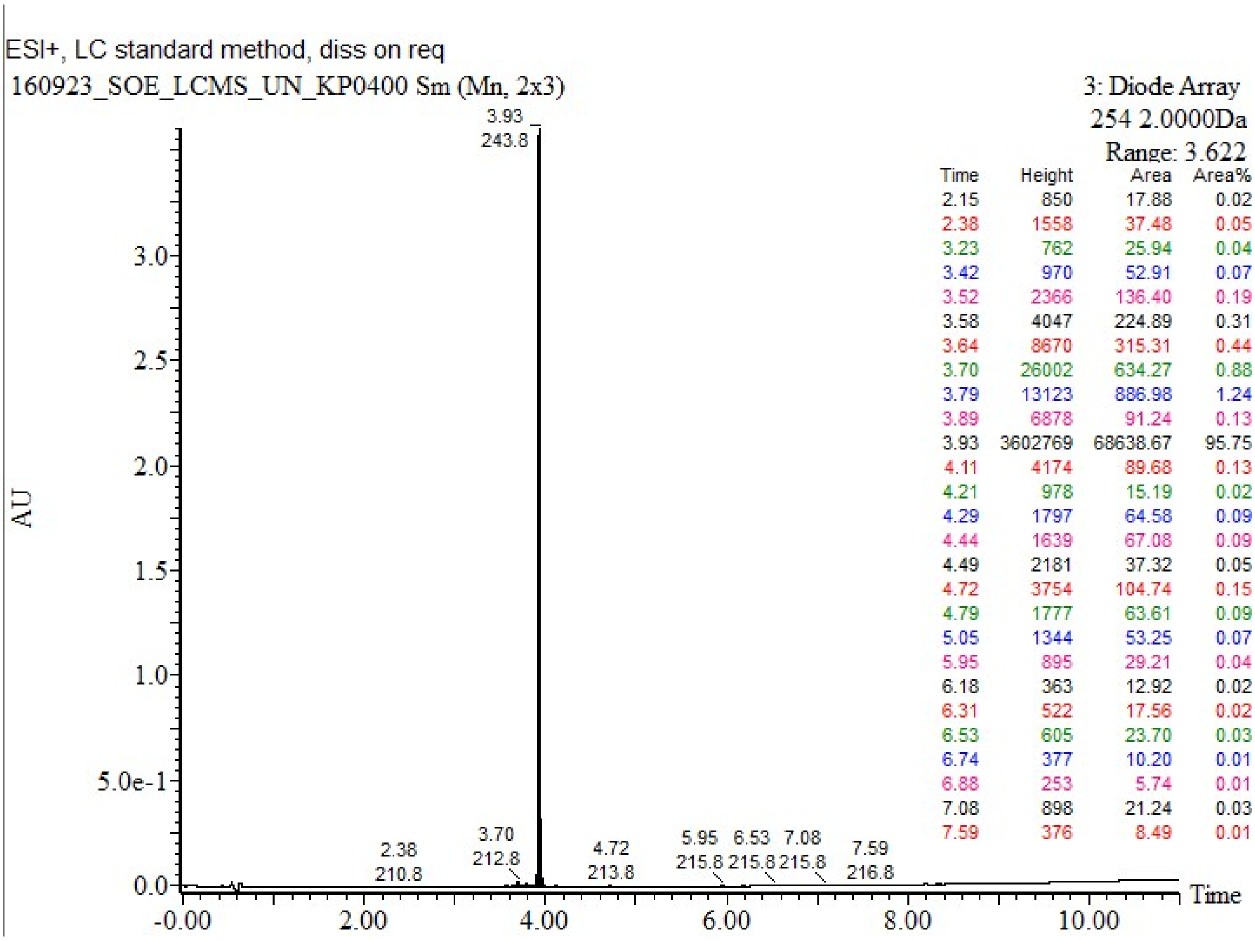

Time	Height	Area	Area%
2.15	850	17.88	0.02
2.38	1558	37.48	0.05
3.23	762	25.94	0.04
3.42	970	52.91	0.07
3.52	2366	136.40	0.19
3.58	4047	224.89	0.31
3.64	8670	315.31	0.44
3.70	26002	634.27	0.88
3.79	13123	886.98	1.24
3.89	6878	91.24	0.13
3.93	3602769	68638.67	95.75
4.11	4174	89.68	0.13
4.21	978	15.19	0.02
4.29	1797	64.58	0.09
4.44	1639	67.08	0.09
4.49	2181	37.32	0.05
4.72	3754	104.74	0.15
4.79	1777	63.61	0.09
5.05	1344	53.25	0.07
5.95	895	29.21	0.04
6.18	363	12.92	0.02
6.31	522	17.56	0.02
6.53	605	23.70	0.03
6.74	377	10.20	0.01
6.88	253	5.74	0.01
7.08	898	21.24	0.03
7.59	376	8.49	0.01

3,3’-Dideoxy-3-(fluorescein-5-yl-carbonylamino)-3’-[4-(thiazol-2-yl)-1*H*-1,2,3-triazol-1-yl]-1,1’-sulfanediyl-di-β-D-galactopyranoside 25

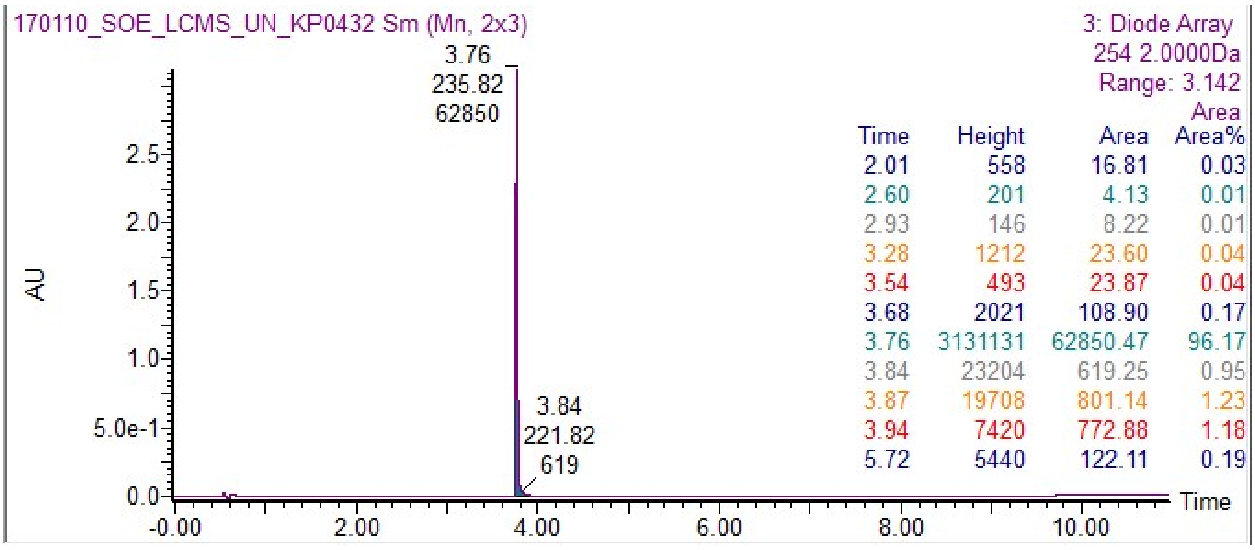

Time	Height	Area	Area Area%
2.01	558	16.81	0.03
2.60	201	4.13	0.01
2.93	146	8.22	0.01
3.28	1212	23.60	0.04
3.54	493	23.87	0.04
3.68	2021	108.90	0.17
3.76	3131131	62850.47	96.17
3.84	23204	619.25	0.95
3.87	19708	801.14	1.23
3.94	7420	772.88	1.18
5.72	5440	122.11	0.19

Figure S1. Fluorescence polarization titration of galectin-1 with fluorescent probe molecule **25**.

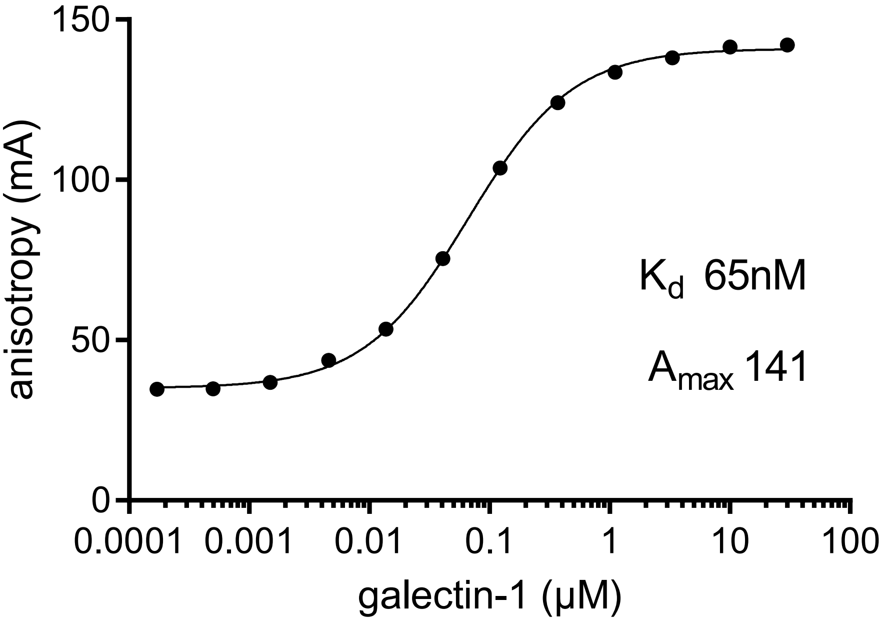

Figure S2. Dose response curves compounds **1**, **6-7**, **12-14** and **19** in a MTT assay.

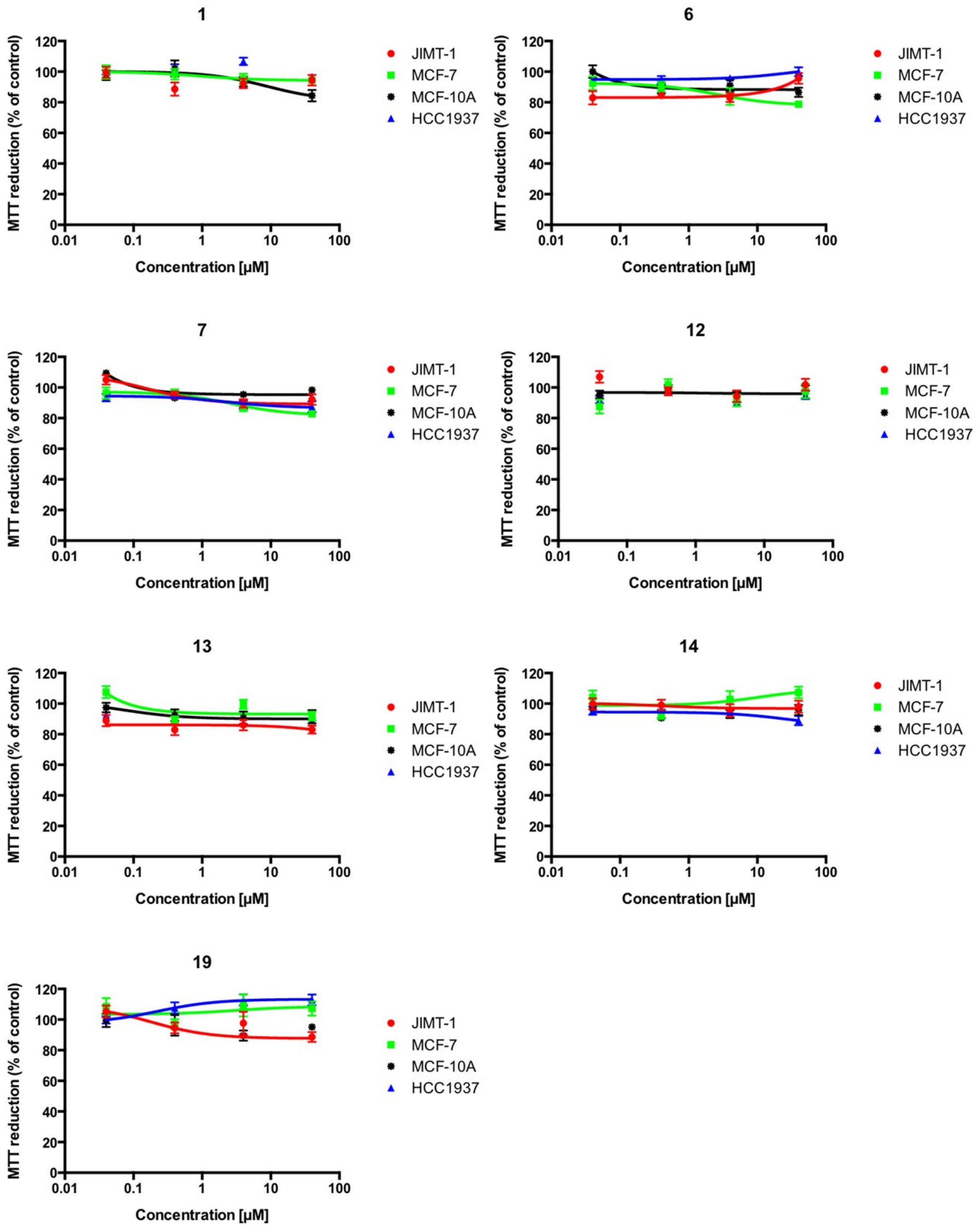

Table S1. Crystallographic data and refinement statistics for galectin-1 in complex with **1**.

Ligand	**1**
PDB code	6F83
Wavelength	1.5418
Resolution range (Å)	40.58–2.20 (2.279–2.20)
Space group	$P2_12_12_1$
Cell dimensions *a*, *b*, *c* (Å)	44.4, 58.7, 112.4
Total reflections	25 972 (1 310)
Unique reflections	14 260 (872)
Completeness (%)	91.71 (57.00)
Multiplicity	1.8 (1.5)
Wilson B-factor	27.85
R_{merge}	0.047 (0.407)
R_{meas}	0.066 (0.575)
R_{pim}	0.047 (0.407)
$I / \sigma I$	12.08 (1.80)
$CC_{1/2}$	0.998 (0.68)
CC*	0.999 (0.9)
Reflections used in refinement	14 253 (871)
Reflections used for *R*free	712 (38)
R_{work}	0.216 (0.289)
R_{free}	0.252 (0.324)
CC_{work}	0.964 (0.791)
CC_{free}	0.942 (0.650)
No. of non-hydrogen atoms	2 315
macromolecules	2 156
ligands	82
solvent	77
Protein residues	268
RMS bond length (Å)	0.012

RMS bond angle (°)	1.59
Ramachandran favored (%)	97.01
Ramachandran allowed (%)	2.56
Ramachandran outliers (%)	0.43
Rotamer outliers (%)	5.09
Clashscore	5.32
Average *B*-factors (Å^2)	16.0
macromolecules	13.8
ligands	55.0
solvent	35.3
Number of TLS groups	2

Statistics for the highest-resolution shell are shown in parentheses
